# Supplementary material for: Selective Functionalisation of 5‐Methylcytosine by Organic Photoredox Catalysis
Source: Angew Chem Weinheim Bergstr Ger. 2023 May 15;135(26):e202304756. doi: 10.1002/ange.202304756 (PMC10953388; doi:10.1002/ange.202304756)
Supplement: Supplementary file 1 — Supporting Information [file ANGE-135-0-s001.pdf]

Angewandte Chemie
Eine Zeitschrift der Gesellschaft Deutscher Chemiker

Supporting Information

Selective Functionalisation of 5-Methylcytosine by Organic Photoredox Catalysis

*M. M. Simpson, C. C. Lam, J. M. Goodman, S. Balasubramanian**

Supporting Information

This document includes:

Materials and Methods
Supplementary Text and Figures
Figs. S1 to S46
Tables S1 to S11
Scheme S1 to S4
Computational Studies

Table of Contents

SECTION 1. MATERIALS AND METHODS 3
SECTION 2. GENERAL PROCEDURES 6
SECTION 3. EXPERIMENTAL DATA 8
Section 3.1: Pyridination of DNA Nucleosides 8
NMR Spectra of 4-Pyr-5mdC: 8
Calculation of Oxidation Potentials for Nucleobases 10
LCMS traces of nucleoside pyridination reactions using 4-cyanopyridine and xanthone 15
LCMS traces of 5mdC pyridination reactions using different cyanopyridine derivatives 24
Section 3.2: Pyridination of Oligonucleotides 29
LCMS traces of oligonucleotide pyridination reactions using 4-cyanopyridine and xanthone 29
Oligonucleotide Product Analysis Using BioPharma Finder 4.1 Oligonucleotide Analysis 31
Section 3.2.1. 5mC 12mer Pyridination Product Analysis 32
Section 3.2.2. 5mC 10mer Pyridination Product Analysis 33
Section 3.2.3. m5C 10mer Pyridination Product Analysis 35
SECTION 4. HALOTAG-BASED ENRICHMENT STUDIES 36
Section 4.1. Synthesis of Chloroalkane-Cyanopyridine 36
Section 4.2. NMR Spectra of Synthesised Compounds 38
Section 4.3. Enrichment Studies of 67nt m5C RNA 42
Section 4.4. Halo-Trap Enrichment of m5C-Oligoribonucleotides 45
SECTION 5. COMPUTATIONAL STUDIES 47
Section 5.1: Computational methodologies 47
Section 5.2: The initial and refined mechanism 48
Section 5.3: Mechanistic revisions 49
Section 5.3.1: Step 2: Deprotonation of the cationic 5mdC radical 49
Section 5.3.2: Step 3: triplet vs doublet vs single pathway 53
Section 5.3.3: Step 4: H addition 54
Section 5.4: Variation in percentage yield 56
Section 5.4.1: Thermodynamic study 56
Section 5.4.2: Kinetic study 58
Section 5.5.: Key structures 61
REFERENCES 64

Section 1. Materials and Methods

Proton nuclear magnetic resonance (1H NMR) spectra were recorded at ambient temperature on a 400 MHz Bruker Avance III HD spectrometer (400 MHz). Chemical shifts (δ) were reported in ppm and quoted to the nearest 0.01 ppm relative to the residual solvent peaks and coupling constants (J) were quoted in Hertz (Hz). Coupling constants were quoted to the nearest 0.1 Hz and multiplicity reported according to the following convention: s = singlet, d = doublet, t = triplet, q = quartet, qnt = quintet, sxt = sextet, spt = septet, oct = octet, m = multiplet, br = broad and associated combinations, e.g., dd = doublet of doublets. Data were reported as follows: chemical shift (multiplicity, coupling constants, number of protons).

Carbon nuclear magnetic resonance (13C NMR) spectra were recorded at ambient temperature on a 400 MHz Bruker Avance III HD spectrometer (101 MHz). Chemical shifts (δ) were reported in ppm and quoted to the nearest 0.1 ppm relative to the residual solvent peaks.

High-resolution mass spectra (HRMS) of small molecules and proteins were conducted using Vion IMS QToF and analysed using Waters UNIFI software.

LCMS spectra were recorded on an Amazon X ESI-MS (Bruker) connected to an Ultimate 3000 LC (Dionex). Nucleosides were analysed using an isocratic gradient of 80% MeCN vs. an aqueous solution of pH 3.7 10 mM NH_4OAc on a Merck SeQuant ZIC-HILIC column (100 Å, 3.5 µm, 150 x 4.6 mm). Single stranded oligodeoxyribonucleotides were analysed using a gradient of 10-30% methanol vs. an aqueous solution of 10 mM triethylamine and 100 mM hexafluoro-2-propanol on a Waters Acquity Premier Oligonucleotide BEH C18 column (130 Å, 1.7 µm, 2.1 x 50 mm). Single stranded oligoribonucleotides were analysed using a gradient of 10-40% methanol vs. an aqueous solution of 7.5 mM triethylamine and 200 mM hexafluoro-2-propanol on a Waters Acquity Premier Oligonucleotide BEH C18 column (130 Å, 1.7 µm, 2.1 x 50 mm). Mass chromatograms shown are base peak chromatograms, UV absorption was recorded at 260 nm.

High-resolution mass spectra (HRMS) of oligonucleotides were recorded on an Orbitrap Exploris 120 (Thermo Scientific™) connected to Vanquish HPLC system (Thermo Scientific™). Oligodeoxyribonucleotides were analysed using a gradient of 2-25% methanol vs. an aqueous solution of 10 mM triethylamine and 100 mM hexafluoro-2-propanol at 0.5 mL/min on a Thermo Scientific™ DNAPac™ RP HPLC column (4.0 µm, 2.1x100mm). Oligoribonucleotides were analysed using a gradient of 5-30% methanol vs. an aqueous solution of 7.5 mM triethylamine and 200 mM hexafluoro-2-propanol at 0.5 ml/min on a Thermo Scientific™ DNAPac™ RP HPLC column (4.0 µm, 2.1x100mm). The column temperature was maintained at 50 °C. The elution was monitored at 260 nm (Vanquish VWD-C). The chromatographic eluent was directly injected into the ion source without prior splitting. Global parameters: ion source type, H-ESI; spray voltage, static; negative ion, 2.5 kV; gas mode, static; sheath gas flow rate, 30 arb; auxiliary gas flow rate, 12 arb; sweep gas flow rate, 5 arb; ion transfer tube temperature, 320 °C; vaporizer temperature, 300 °C. Ions were scanned by use of a negative polarity mode over a full-scan range of m/z 550-2000 with a resolution of 60000. ddMS^2 parameters: collision energy mode, stepped; collision energy type, normalized; HCD collision energies, 14,16,18%; orbitrap resolution, 30000. Results were analysed using Thermo BioPharma Finder 4.1.

Oligodeoxyribonucleotides and short oligoribonucleotides were custom synthesised and HPLC purified by ATDBio, Sigma-Aldrich or in-house and used without further purification after

dissolution into milliQ H2O. Oligoribonucleotides RNA-M and RNA-C were prepared using the MEGAScript® Kit following the manufacturers protocol and purified using NucAway™ Spin Columns.

ODN	Sequence
5mC 12mer	AGACCA[5mC]AACCA
5mC 10mer	AGACCTAC[5mC]A
5mC 54mer	CCATGTAC[5mC]TCCGATGCA[5mC]ACGTACC[5mC]GATGC TACCTGATTAGGC[5mC]ATTCTCAT
m5C RNA 10mer	agaccuac[m5c]a
RNA-M DNA Template	CATCCTTCTCACTACTCACTACCTCATGAATCTCTACGTCATA CCAATCAACAACAACTATACACCCTATAGTGAGTCGTATTA
RNA-C DNA template	CATGTCCGCTCTAGTCGCTAGGTGCTAATGATACTACGCAAA CCGAAGAACGAGAAGCAGACACCCTATAGTGAGTCGTATTA
Transcription Primer	TAATACGACTCACTATAGGG
RNA-M	ggguguauaguuguuguugauugguauga[m5c]guagagauu[m5c]augagguagug aguagugagaaggaug
RNA-C	gggugucugcuucucguucuucgguuugcguaguaucauuagcaccuagcgacuagagcg gacaug
RNA-M Forward Primer	CATCCTTCTCACTACTCACTACC
RNA-M Reverse Primer	GGTGTATAGTTGTTGTTGATTGG
RNA-C Forward Primer	CATGTCCGCTCTAGTCGCTAGG
RNA-C Reverse Primer	TGTCTGCTTCTCGTTCTTCGG

Reagents were obtained from Sigma-Aldrich, Acros, Alfa Aesar, TCI, or Santa Cruz Biotechnology and used without further purification. Enzyme solutions were obtained from Zymo and used directly.

Oligonucleotide reaction buffers were prepared by combining milliQ H_2O (34.5 mL), PBS (pH adjusted using dilute HCl, Gibco Life Technologies, 5 mL), MeCN (10 mL) and NaCl (5M, Sigma, 0.5 mL). The resulting solution was then degassed by at least five cycles of freeze-pump-thaw degassing.

Photoredox reactions were carried out using a HepatoChem PhotoRedOx Box equipped with a 365 nm EvoluChem LED. All reaction vials were placed in the two front and central vial positions of the 2 mL vial holder. Reactions were carried out under argon atmosphere unless otherwise stated. Reactions were monitored by LCMS.

Oligonucleotide reactions were concentrated using prewashed 3 kDa Amicon® Ultra-0.5 mL centrifugal size exclusion filters (Merck Millipore). Samples were centrifuged for 40 minutes at 14000 rpm at 4°C then rinsed with 450 µL milliQ and centrifuged for a further 50 minutes at 14000 rpm at 4°C. Concentrated oligonucleotide reactions were then filtered through a prewashed Mini Quick Spin Oligo Column (Roche). The obtained mixture was analysed directly by LCMS.

qPCR was performed using a CFX96 Real-TimeSystem (BioRad), and data was processed using CFX software manager 3.1 (BioRad). qPCR reactions (volume: 20 μL) contained RNA standard or sample mixtures (2.5 μL), the corresponding forward and reverse primers (0.3 μM each), and Luminaris Color HiGreen qPCR Master Mix (2X) (Thermo Scientific, 10 μL). Reactions were run according to the manufacturer's protocol. Standard curves were made to determine the amounts of target RNA in the analysed samples.

Section 2. General Procedures

General Procedure A: pyridination of 5mdC under argon

A 2 mL LCMS vial was charged with 5mdC (200 mM in DMSO, 125 µL) and 2'-deoxyinosine (internal standard, 100 mM in DMSO, 25 µL). Then the solvent was removed by GeneVac EZ2 Elite (high BP, max temp 50 °C). Then xanthone (100 mM in $CHCl_3$, 50 µL) and 4-cyanopyridine (1.2 M in $CHCl_3$, 125 µL) were added to the vial and the solvent was again removed by GeneVac (very low BP, max temp 50 °C). The vial was sparged with argon for 10 minutes then degassed 20% MeCN in milliQ H_2O (500 µL) was added under a continuous stream of argon. Sparging with argon was continued for a further 10 minutes. The mixture was sonicated in order to ensure all components were fully dissolved and a 0-minute sample was taken for LCMS analysis. After sparging with argon again for 10 minutes the vial was placed in a PhotoRedOx Box and irradiated for 20 hours. The obtained mixture was diluted in 1:1 MeCN:H_2O then analysed by LCMS.

Scheme S1: Step-by-step set up for the pyridination reaction

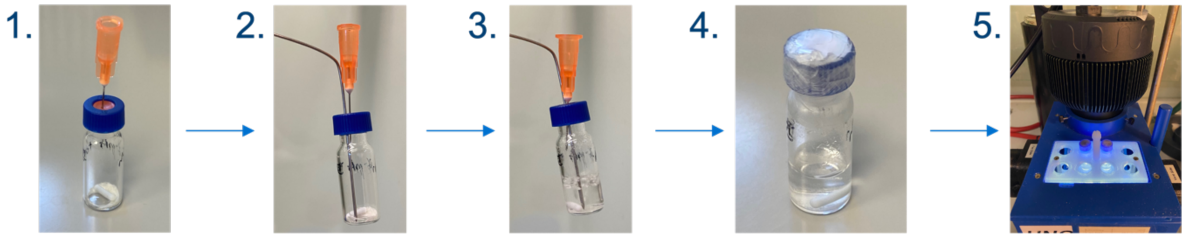

1) Prepare vial containing magnetic stirrer, xanthone and cyanopyridine. 2) Sparge with argon to remove air. 3) Add degassed solvent under a continuously flow of argon via syringe. Continue sparging. 4) Seal vial with parafilm. 5) Irradiate with 365nm light for required duration.

General Procedure B: pyridination of 5mdC under air

A 2 mL LCMS vial was charged with 5mdC (200 mM in DMSO, 125 µL) and 2'-deoxyinosine (internal standard, 100 mM in DMSO, 25 µL). Then the solvent was removed by GeneVac EZ2 Elite (high BP, max temp 50 °C). Then xanthone (100 mM in $CHCl_3$, 50 µL) and 4-cyanopyridine (1.2 M in $CHCl_3$, 125 µL) were added to the vial and the solvent was again removed by GeneVac (very low BP, max temp 50 °C). 20% MeCN in milliQ H_2O (500 µL) was added under atmospheric conditions. The mixture was sonicated in order to ensure all components were fully dissolved and a 0-minute sample was taken for LCMS analysis. The vial was sealed then placed in a PhotoRedOx Box and irradiated for 20 hours. The obtained mixture was diluted in 1:1 MeCN:H_2O then analysed by LCMS.

General Procedure C: pyridination of 5mC oligonucleotides with 4-cyanopyridine

A 2 mL LCMS vial was charged with xanthone (100 mM in $CHCl_3$, 100 µL) and 4-cyanopyridine (100 mM in $CHCl_3$, 25 µL) then the solvent was removed by GeneVac (very low BP, max temp 50 °C). Degassed pH 7.1 oligonucleotide reaction buffer (500 µL) was added under a continuous stream of argon and sparging with argon was continued for a further 10 minutes. Oligonucleotide substrate (50 µM in milliQ H_2O, 10 µL) was then added and sparging with argon was continued for a further 10 minutes. The vial was placed in a PhotoRedOx Box and irradiated for 2 hours at 4 °C. The reaction was concentrated using

prewashed 3 kDa Amicon® Ultra-0.5 mL centrifugal size exclusion filters (Merck Millipore) then filtered through a prewashed Mini Quick Spin Oligo Column (Roche). The obtained mixture was analysed directly by LCMS.

General Procedure D: pyridination of m5C-containing RNA oligonucleotides

A 2 mL LCMS vial was charged with xanthone (100 mM in $CHCl_3$, 100 µL) and HaloLig-CP (100 mM in $CHCl_3$, 25 µL) then the solvent was removed by GeneVac (very low BP, max temp 50 °C). Degassed pH 7.1 oligonucleotide reaction buffer (500 µL) was added under a continuous stream of argon and sparging with argon was continued for a further 10 minutes. A 1:1 mixture of RNA-M and RNA-C (1 µM each in milliQ H_2O, 10 µL) then heparin (1 µg/µL, 6 µL) were added and sparging with argon was continued for a further 10 minutes. The vial was placed in a PhotoRedOx Box and irradiated for 2 hours at 4 °C. The reaction was purified using RNA Clean & Concentrator-5 (Zymo).

General Procedure E: DNA degradase plus digestion of 54mer oligodeoxyribonucleotide pyridination product

5mC 54mer pyridination reaction product was purified using ssDNA/RNA Clean & Concentrator (Zymo) then digested to constituent mononucleosides using 5 units of DNA Degradase Plus TM (Zymo) with 10x Degradase buffer at 37°C for 6 hours in 50 µL reaction volume. The digestion was purified through a prewashed 10 KDa Amicon® Ultra-0.5 mL centrifugal size exclusion filter (Merck Millipore). The filtrate was lyophilised then resuspended in milliQ and analysed by LCMS.

Section 3. Experimental Data

Section 3.1: Pyridination of DNA Nucleosides

Nucleoside reactions were carried out in duplicate according to general procedure A. 5mdC was replaced with other DNA mononucleosides (dissolved in either DMSO or 1:1 DMSO:milliQ H_2O) as appropriate.

Scheme S2: Pyridination of 5mdC using 4-cyanopyridine and xanthone

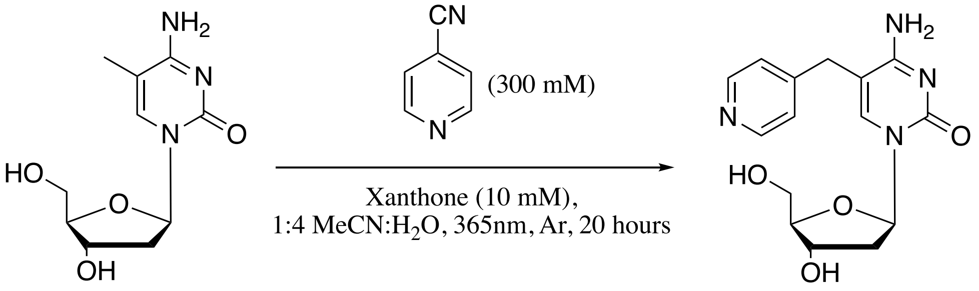

NMR Spectra of 4-Pyr-5mdC:

^{1}H NMR (400 MHz, DMSO-d_6) δ 8.47 (d, J = 5.0 Hz, 2H), 7.73 (s, 1H), 7.23 (d, J = 5.0 Hz, 2H), 6.15 (t, J = 6.6 Hz, 1H), 5.20 (d, J = 4.2 Hz, 1H), 4.97 (d, J = 5.4 Hz, 1H), 4.19 (dd, J = 6.3, 3.3 Hz, 1H), 4.11 (q, J = 5.0 Hz, 1H), 3.76 (d, J = 3.7 Hz, 1H), 3.67 (s, 2H), 3.50 (qd, J = 11.6, 5.8 Hz, 2H), 3.18 (d, J = 3.1 Hz, 2H), 2.13 (ddd, J = 13.2, 6.1, 3.4 Hz, 1H), 1.96 (dt, J = 13.2, 6.6 Hz, 1H).

^{13}C NMR (101 MHz, DMSO-d_6) δ 164.90, 155.25, 149.97, 148.69, 140.69, 124.21, 103.56, 87.69, 85.46, 70.77, 61.74, 49.07, 40.80, 32.00.

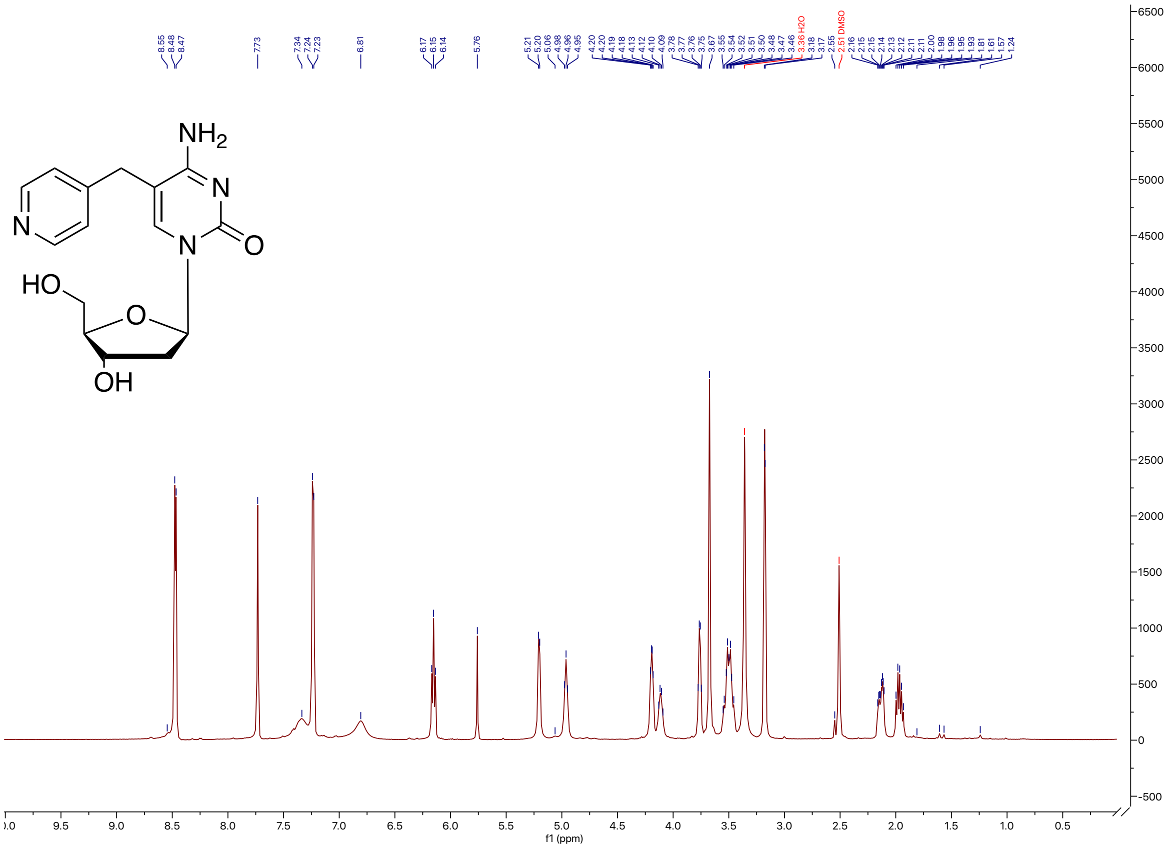

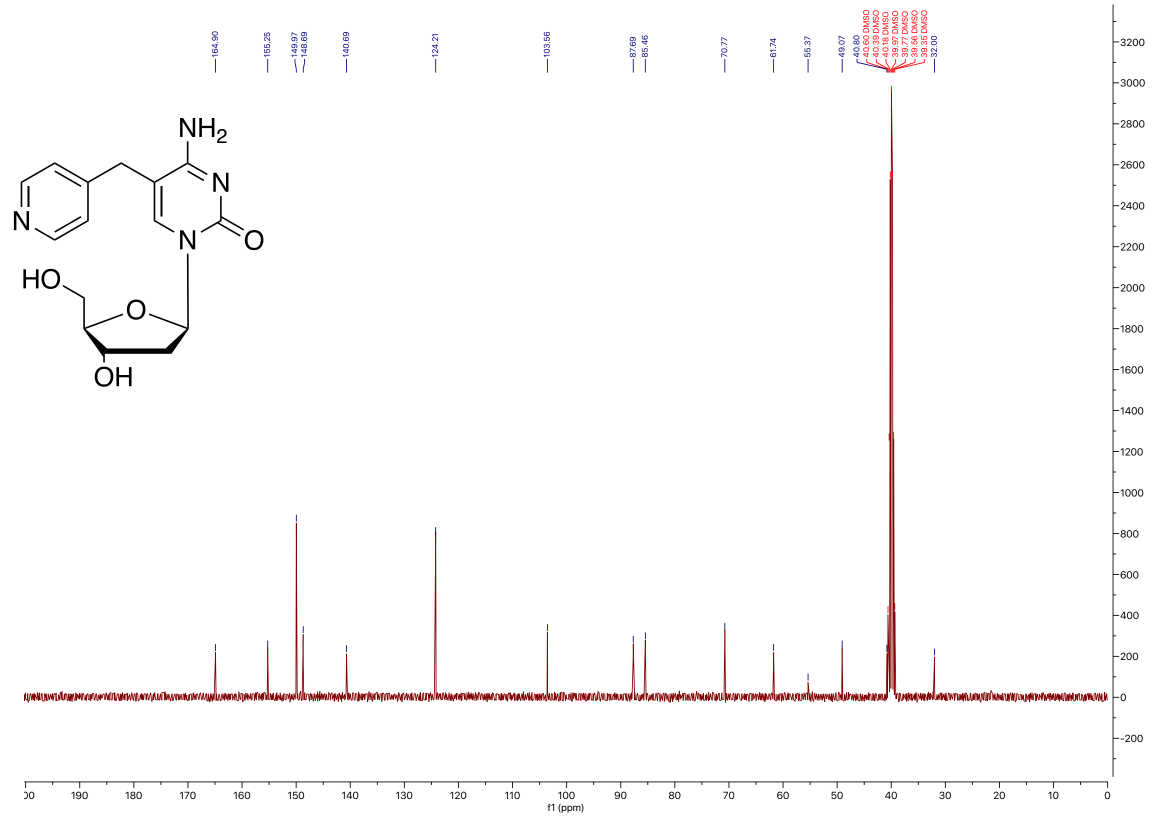

Figure S1: ^{1}H NMR (top) and ^{13}C NMR (bottom) spectra of 4-Pyr-5mdC.

Calculation of Oxidation Potentials for Nucleobases

Table S1: Calculated oxidation potentials for nucleobases

Base	Calculated Ionization Potential, IP (eV)[1]	Oxidation Potential, E^{o} (V vs NHE)[a]
Guanine (N9-H)	8.13	1.52
Adenine (N9-H)	8.49	1.82
Cytosine (N1-H)	8.79	2.07
Thymine (N1-H)	9.14	2.36
5-methylcytosine (N1-H)	8.50	1.83

[a]Calculated from ionization potential values in the previous column using the equation $E = 0.827\mathrm{IP} - 5.20$.[2]

Table S2: LCMS data of nucleoside pyridination reactions using 4-cyanopyridine and xanthone

Nucleoside	Replicate	Time/h	dI	dNucleoside	dN/dI	Pyr	Pyr/dI
5mdC	1	0	63.884	342.268	5.358	-	-
5mdC	2	0	68.663	398.949	5.810	-	-
5mdC_O_2_N	1	0	49.790	338.118	6.791	-	-
5mdC_O_2_N	2	0	48.013	320.425	6.674	-	-
dT	1	0	48.684	512.440	10.526	-	-
dT	2	0	64.419	619.318	9.614	-	-
dC	1	0	74.518	562.879	7.554	-	-
dC	2	0	68.356	544.058	7.959	-	-
dG	1	0	51.817	841.105	16.232	-	-
dG	2	0	50.653	811.157	16.014	-	-
dA	1	0	50.941	849.197	16.670	-	-
dA	2	0	52.697	917.178	17.405	-	-
5mdC_dark	1	0	58.575	373.658	6.379	-	-
5mdC_dark	2	0	59.020	409.674	6.941	-	-
dT_O2	1	0	53.196	568.402	10.685	-	-
dT_O2	2	0	57.666	637.746	11.059	-	-
dG_O2	1	0	55.679	946.401	16.997	-	-
dG_O2	2	0	47.975	747.802	15.587	-	-
dA_O2	1	0	131.060	2708.920	20.669	-	-
dA_O2	2	0	46.595	909.297	19.515	-	-
dC_O2	1	0	54.730	495.863	9.060	-	-
dC_O2	2	0	48.006	470.548	9.802	-	-
5hmdC	1	0	49.826	358.639	7.198	-	-
5hmdC	2	0	60.916	409.436	6.721	-	-
5hmdC_O2	1	0	41.459	331.059	7.985	-	-
5hmdC_O2	2	0	45.823	341.829	7.460	-	-
5mdC	1	20	64.916	34.147	0.526	147.208	2.268
5mdC	2	20	62.603	16.832	0.269	150.765	2.408
5mdC_O2	1	20	59.614	92.431	1.550	156.992	2.633
5mdC_O2	2	20	53.277	52.062	0.977	168.137	3.156
dT	1	20	59.878	435.980	7.281	21.567	0.360
dT	2	20	75.880	465.850	6.139	15.941	0.210
dC	1	20	64.327	481.816	7.490	0.000	0.000
dC	2	20	59.921	468.588	7.820	0.000	0.000
dG	1	20	56.836	900.440	15.843	0.000	0.000
dG	2	20	55.452	848.071	15.294	0.000	0.000
dA	1	20	49.112	796.582	16.220	0.000	0.000
dA	2	20	58.759	928.500	15.802	0.000	0.000
5mdC_dark	1	20	66.013	419.684	6.358	0.000	0.000
5mdC_dark	2	20	61.078	422.676	6.920	0.000	0.000

dT_O2	1	20	56.549	149.106	2.637	24.019	0.425
dT_O2	2	20	52.446	191.326	3.648	20.565	0.392
dG_O2	1	20	52.326	440.979	8.428	0.000	0.000
dG_O2	2	20	53.753	543.335	10.108	0.000	0.000
dA_O2	1	20	74.197	723.807	9.755	0.000	0.000
dA_O2	2	20	63.615	676.434	10.633	0.000	0.000
dC_O2	1	20	40.923	349.371	8.537	0.000	0.000
dC_O2	2	20	43.471	347.493	7.994	0.000	0.000
5hmdC	1	20	52.930	119.980	2.267	12.330	0.233
5hmdC	2	20	60.845	147.464	2.424	10.182	0.167
5hmdC_O2	1	20	42.107	4.072	0.097	11.058	0.263
5hmdC_O2	2	20	40.670	1.819	0.045	10.173	0.250

Table S3: LCMS analysis of nucleoside pyridination reactions using 4-cyanopyridine and xanthone

Nucleoside	Time/h	Average dN/dI	% Remaining	Average Pyr/dI	% Pyr
5mdC	0	5.584	-	-	-
5mdC_O2	0	6.732	-	-	-
dT	0	10.070	-	-	-
dC	0	7.756	-	-	-
dG	0	16.123	-	-	-
dA	0	17.037	-	-	-
5mdC_dark	0	6.660	-	-	-
dT_O2	0	10.872	-	-	-
dG_O2	0	16.292	-	-	-
dA_O2	0	20.092	-	-	-
dC_O2	0	9.431	-	-	-
5hmdC	0	6.960	-	-	-
5hmdC_O2	0	7.722	-	-	-
5mdC	20	0.397	7.118	2.338	41.869
5mdC_O2	20	1.264	18.773	2.895	42.997
dT	20	6.710	66.637	0.285	2.832
dC	20	7.655	98.694	0.000	0.000
dG	20	15.568	96.559	0.000	0.000
dA	20	16.011	93.974	0.000	0.000
5mdC_dark	20	6.639	99.681	0.000	0.000
dT_O2	20	3.142	28.903	0.408	3.757
dG_O2	20	9.268	56.884	0.000	0.000
dA_O2	20	10.194	50.737	0.000	0.000
dC_O2	20	8.265	87.641	0.000	0.000
5hmdC	20	2.345	33.697	0.200	2.876
5hmdC_O2	20	0.071	0.916	0.256	3.320

Table S4A: LCMS data of 5mdC reactions with xanthone

	Replicate	Time/h	dI	5mdC	5hmdC	5fdC
Argon	1	0	125.85	913.96		
Argon	2	0	167.27	1207.91		
O_2	1	0	193.30	1409.03		
O_2	2	0	184.75	1327.58		
Argon	1	20	168.81	721.87	75.90	26.89
Argon	2	20	188.36	986.63	49.33	7.35
O_2	1	20	211.25	605.80	146.23	291.97
O_2	2	20	146.84	287.09	102.43	187.41

Table S4B: LCMS analysis of 5mdC reactions with xanthone

	Time/h	Average 5mdC/dI* $E260_{5mdC}$	% 5mdC	Average 5hmdC/dI *$E260_{5hmdC}$	% 5hmdC	Average 5fdC/dI* $E260_{5fdC}$	% 5fdC
Ar	0	1.270					
O_2	0	1.270					
Ar	20	0.835	65.69%	0.041	3.22%	0.009	0.69%
O_2	20	0.423	33.30%	0.080	6.29%	0.118	9.26%

E260 values were used from Glenn Research (https://www.glenresearch.com/media/productattach/e/x/extinctiontablefluorescencedata.pdf). $E260_{5mdC}$=5.7, $E260_{5hmC}$=8.7, $E260_{5fC}$=11.3.

LCMS traces of nucleoside pyridination reactions using 4-cyanopyridine and xanthone

This is a collection of LC traces of the 4-cyanopyridine nucleoside pyridination reaction described in table S2. For figures S2-14: the first and second traces correspond to the base peak MS chromatogram and UV_{260} chromatogram of the first replicate respectively, the third and fourth traces correspond to the base peak MS chromatogram and UV_{260} chromatogram of the second replicate respectively.

5-methyl-2'-deoxycytidine and 4-cyanopyridine under argon

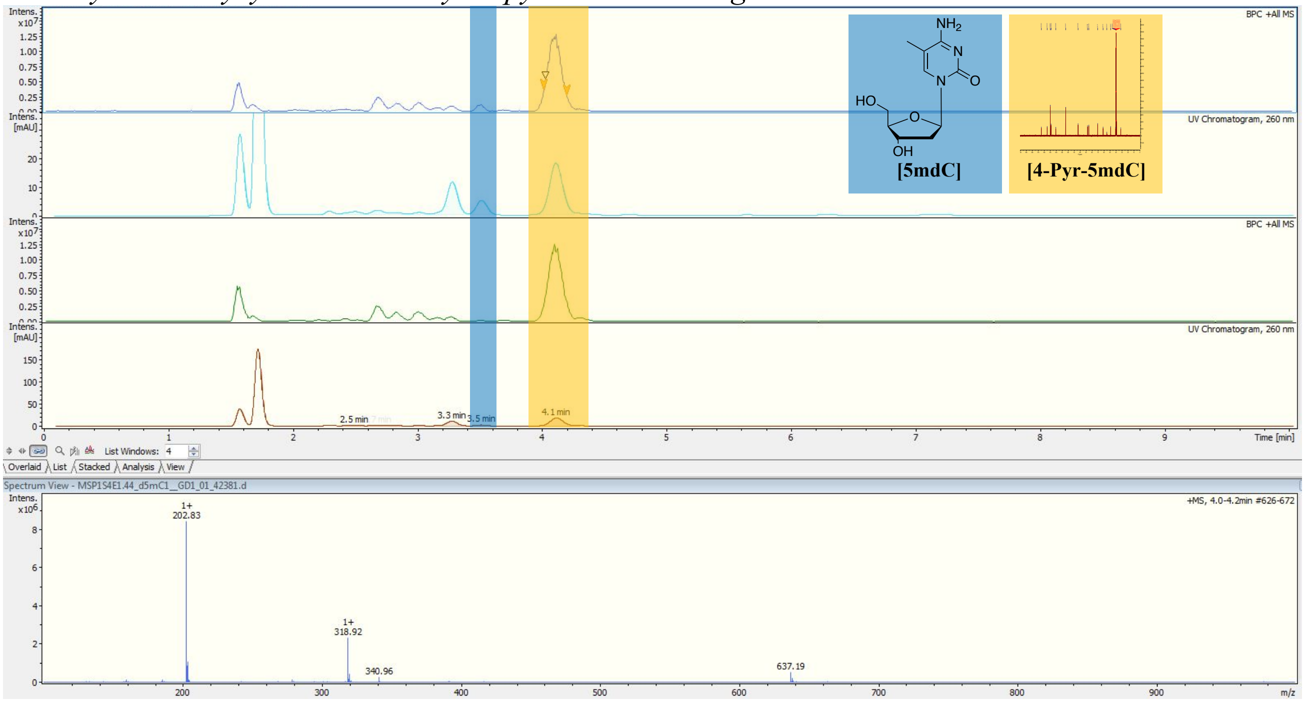

Figure S2: LCMS traces of the nucleoside reaction mixtures after pyridination reaction conditions following general procedure A on 5mdC. The MS shows the extracted mass spectrum of the 5mdC pyridination product. From left to right: small peaks corresponding to pre-HCN elimination product (m/z increase of +104, 2.7-3.0 min), dI (3.3 min), 5mdC (3.5 min), 4-Pyr-5mdC (4.1 min).

Thymidine and 4-cyanopyridine under argon

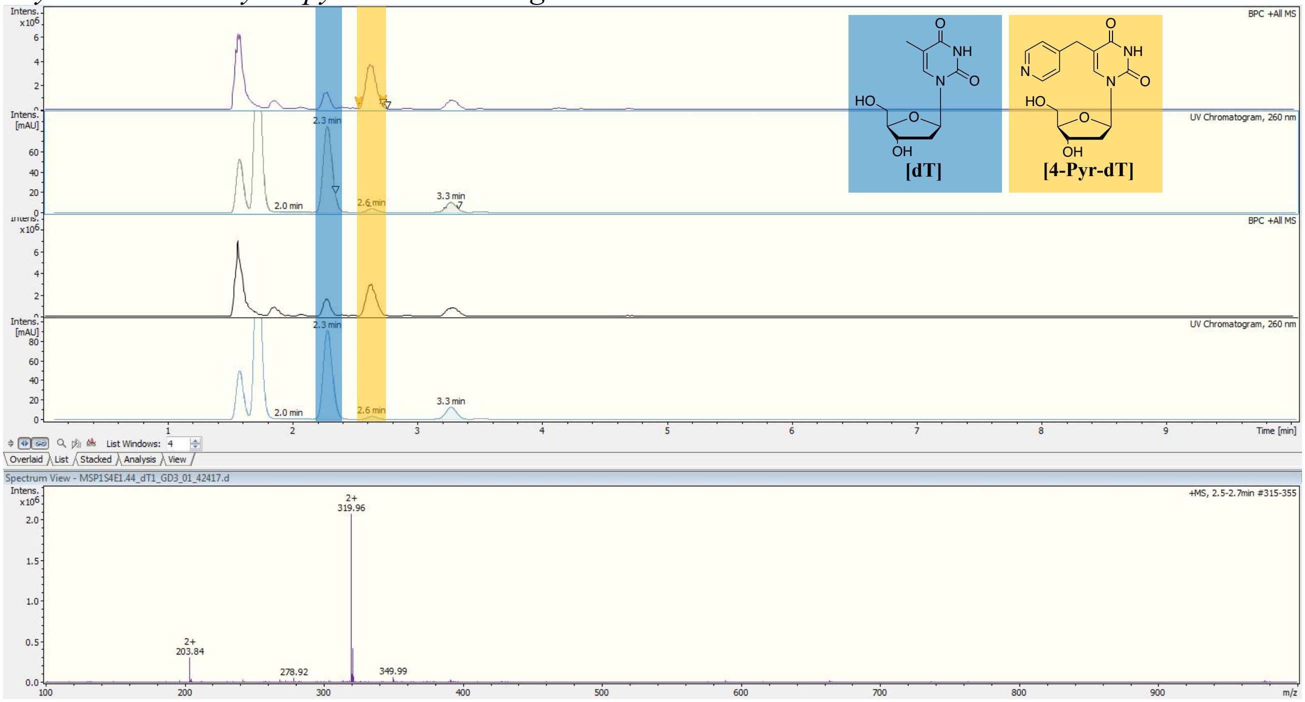

Figure S3: LCMS traces of the nucleoside reaction mixtures after pyridination reaction conditions following general procedure A on dT. The MS shows the extracted mass spectrum of the dT pyridination product. From left to right: Minisci product (m/z increase of +102, 2.0 min), dT (2.3 min), 4-Pyr-dT (2.6 min), dI (3.3 min).

2'-deoxycytidine and 4-cyanopyridine under argon

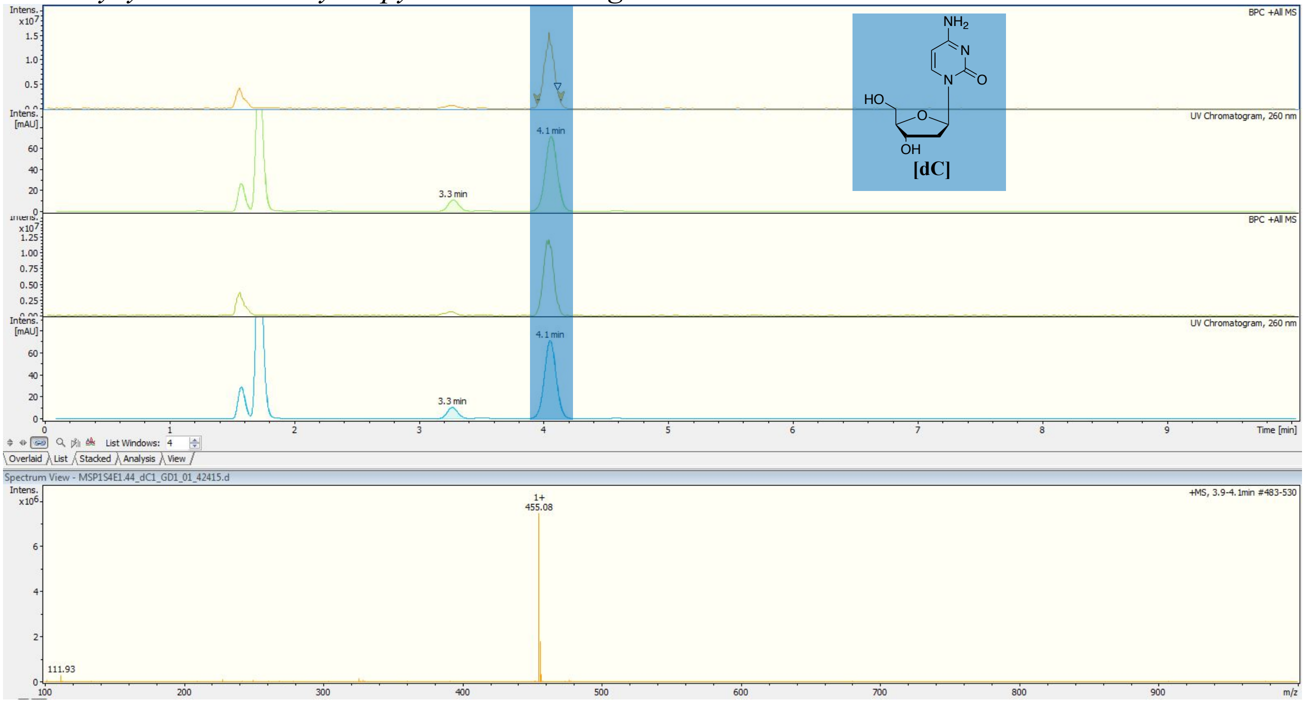

Figure S4: LCMS traces of the nucleoside reaction mixtures after pyridination reaction conditions following general procedure A on dC. The MS shows the extracted mass spectrum of dC. From left to right: dI (3.3 min), dC (4.1 min).

2'-deoxyguanosine and 4-cyanopyridine under argon

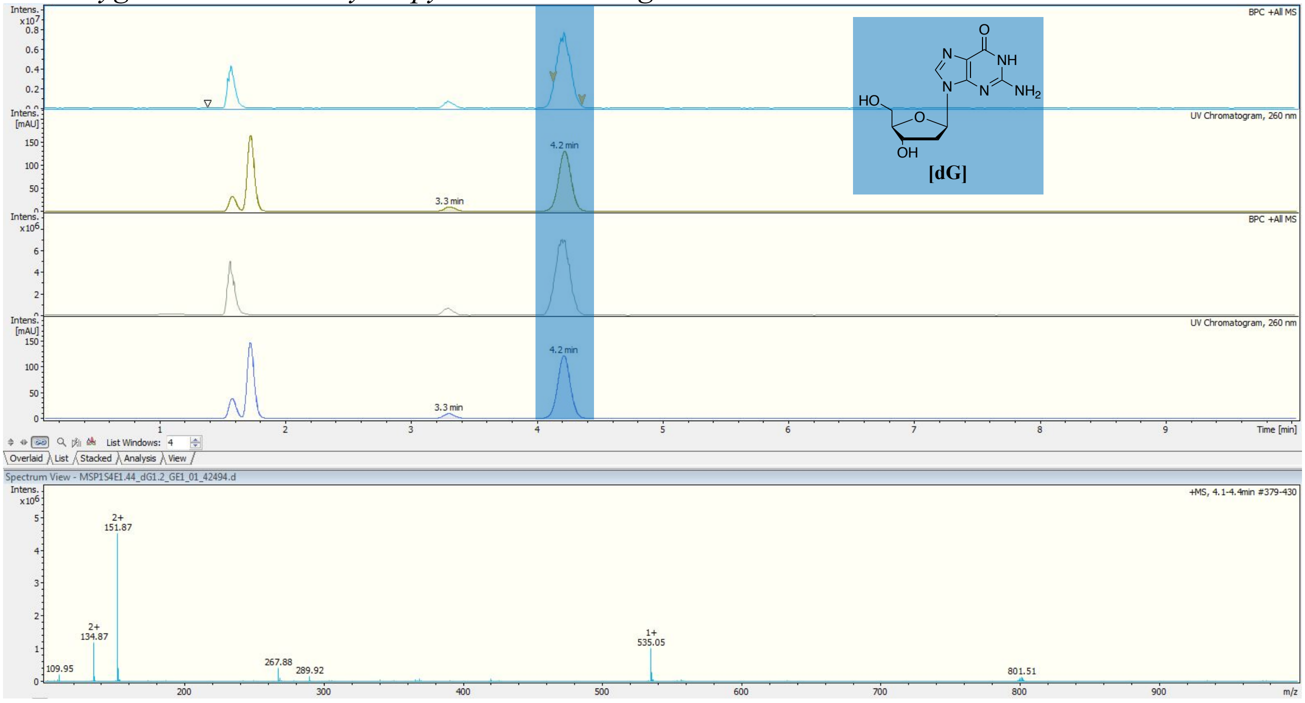

Figure S5: LCMS traces of the nucleoside reaction mixtures after pyridination reaction conditions following general procedure A on dG. The MS shows the extracted mass spectrum of dG. From left to right: dI (3.3 min), dG (4.2 min).

2'-deoxyadenosine and 4-cyanopyridine under argon

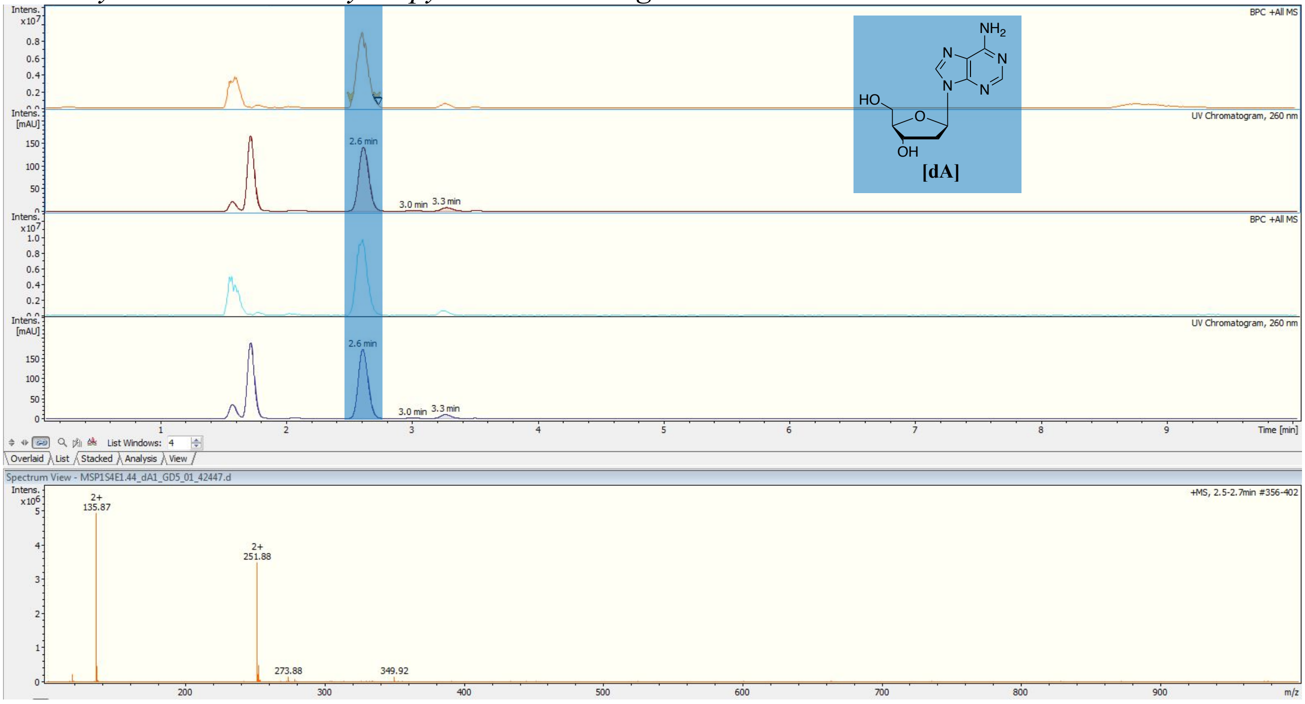

Figure S6: LCMS traces of the nucleoside reaction mixtures after pyridination reaction conditions following general procedure A on dA. The MS shows the extracted mass spectrum of dA. From left to right: dA (2.6 min), A base (3.0 min), dI (3.3 min).

5-hydroxymethyl-2'-deoxycytidine and 4-cyanopyridine under argon

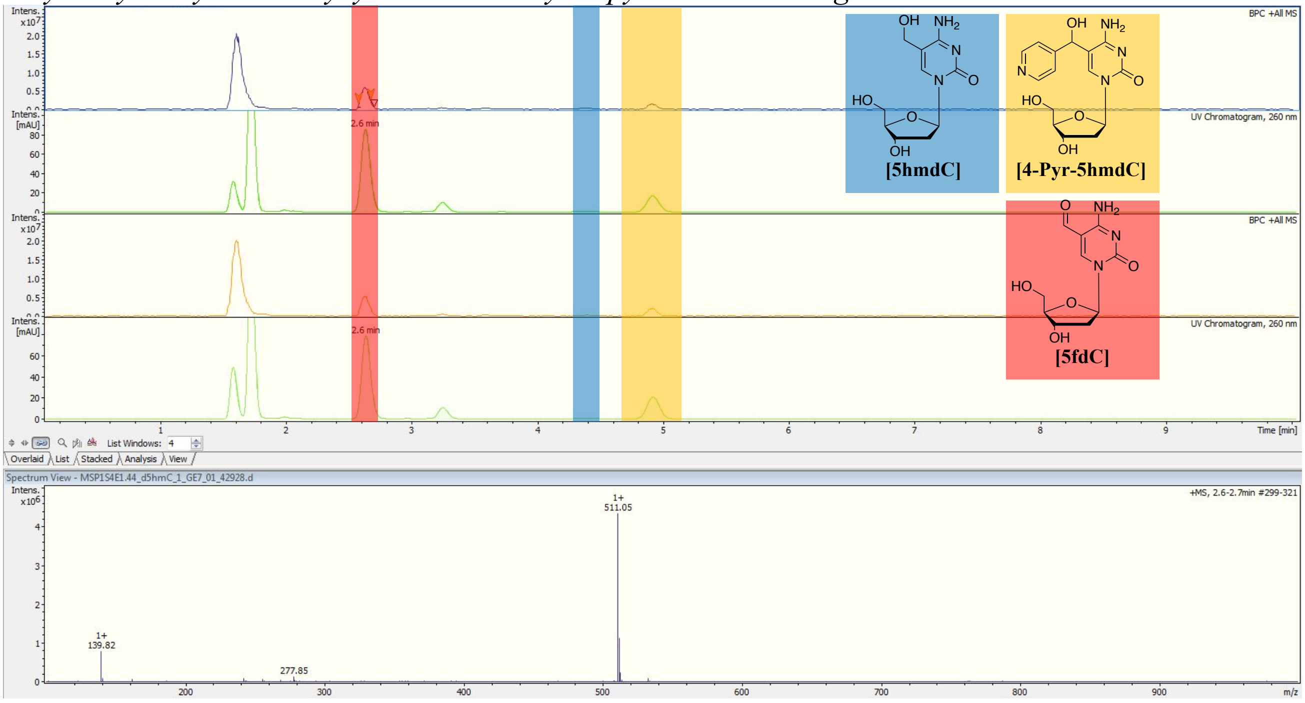

Figure S7: LCMS traces of the nucleoside reaction mixtures after pyridination reaction conditions following general procedure A on 5hmdC. The MS shows the extracted mass spectrum of the 5fdC. From left to right: 5fdC (2.6 min), dI (3.3 min), 4-Pyr-5hmdC (4.4 min), 5hmdC (4.9 min).

5-methyl-2'-deoxycytidine and 4-cyanopyridine under air

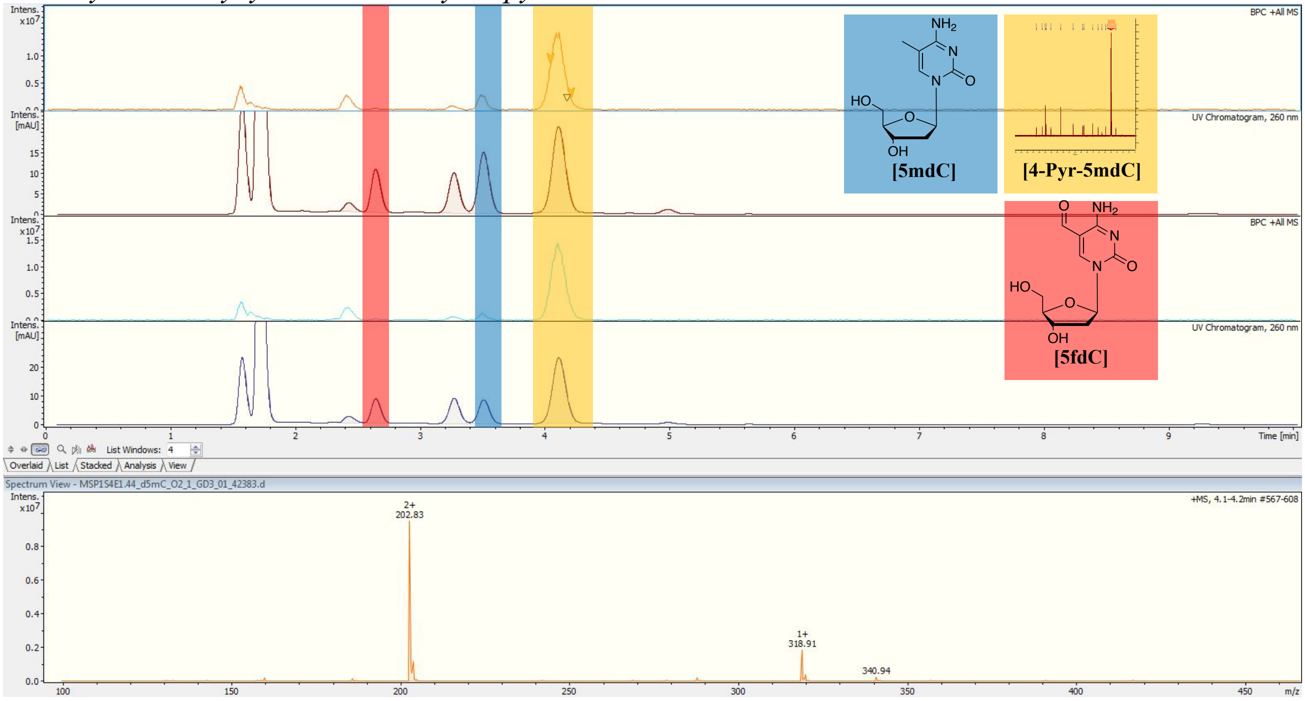

Figure S8: LCMS traces of the nucleoside reaction mixtures after pyridination reaction conditions following general procedure B on 5mdC. The MS shows the extracted mass spectrum of the 5mdC pyridination product. From left to right: Minisci product (m/z increase of +102, 2.4 min), 5fdC (2.6) min), dI (3.3 min), 5mdC (3.5 min), 4-Pyr-5mdC (4.1 min).

Thymidine and 4-cyanopyridine under air

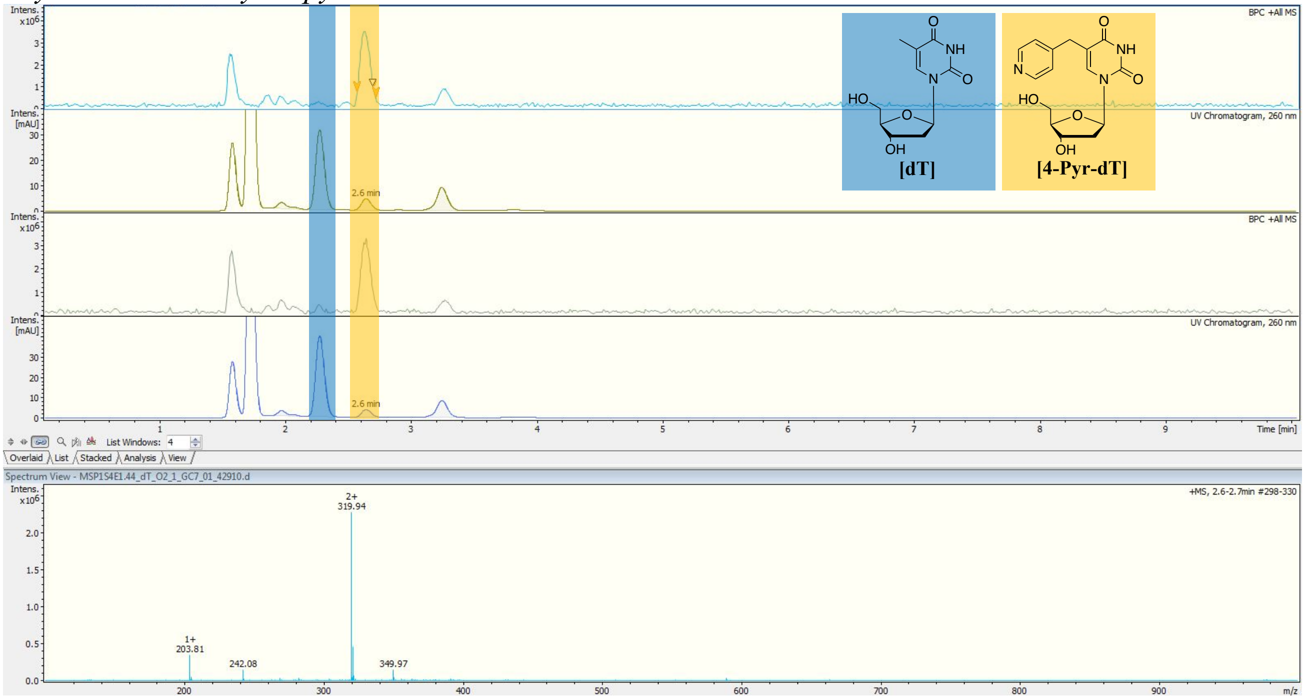

Figure S9: LCMS traces of the nucleoside reaction mixtures after pyridination reaction conditions following general procedure B on dT. The MS shows the extracted mass spectrum of the dT pyridination product. From left to right: Minisci product (m/z increase of +102, 2.0 min), dT (2.3 min), 4-Pyr-5T (2.6 min), dI (3.3 min).

2'-deoxycytidine and 4-cyanopyridine under air

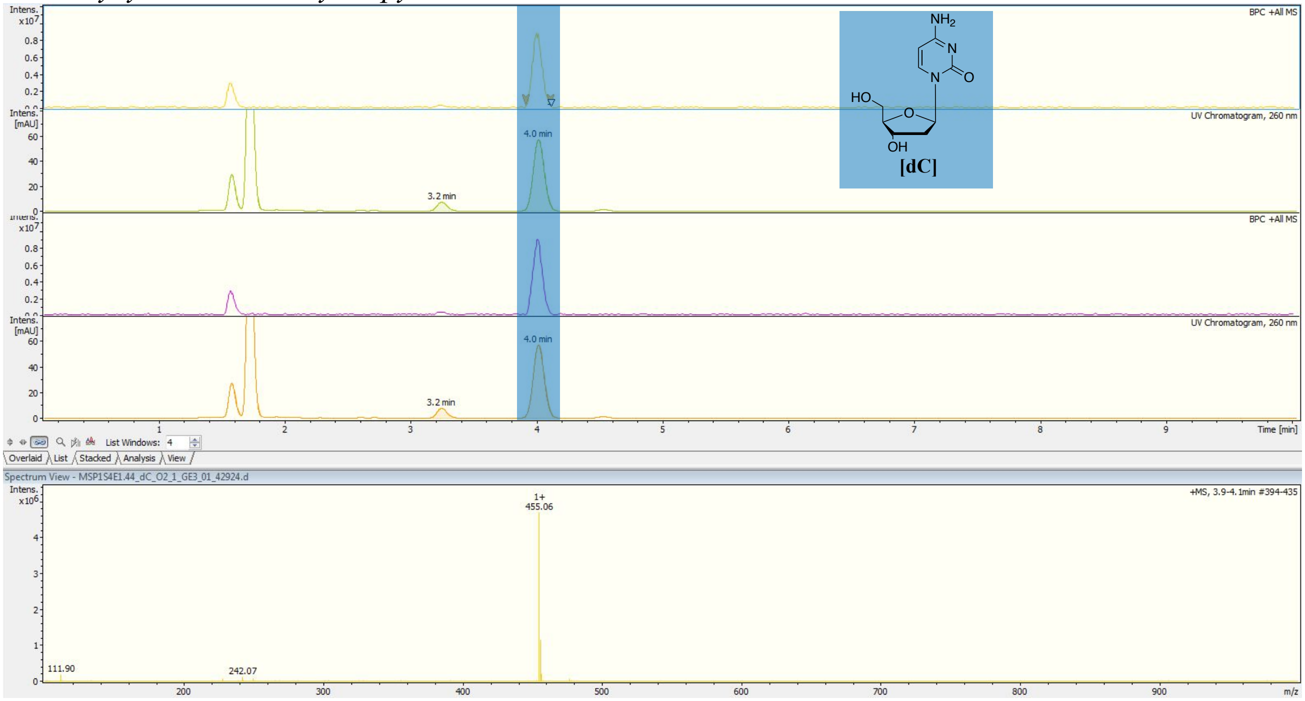

Figure S10: LCMS traces of the nucleoside reaction mixtures after pyridination reaction conditions following general procedure B on dC. The MS shows the extracted mass spectrum of dC. From left to right: dI (3.3 min), dC (4.1 min), C base (4.5 min).

2’-deoxyguanosine and 4-cyanopyridine under air

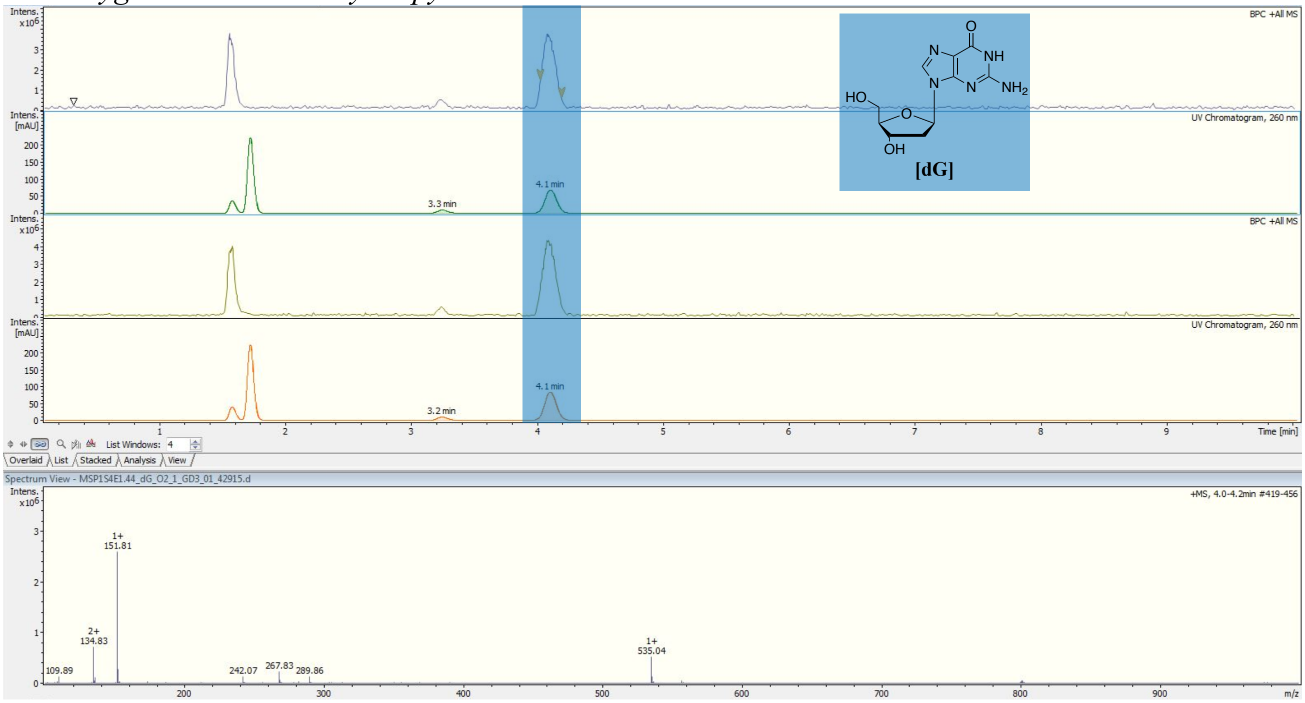

Figure S11: LCMS traces of the nucleoside reaction mixtures after pyridination reaction conditions following general procedure B on dG. The MS shows the extracted mass spectrum of dG. From left to right: dI (3.3 min), dG (4.2 min).

2’-deoxyadenosine and 4-cyanopyridine under air

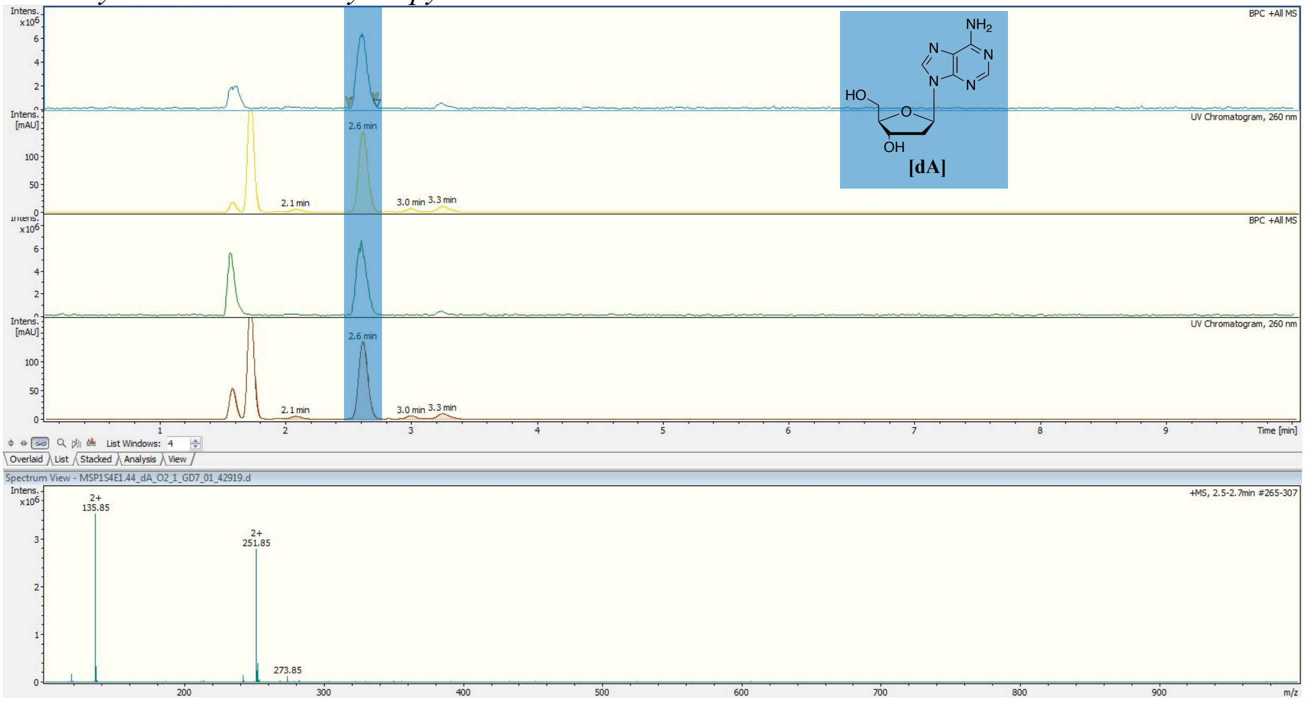

Figure S12: LCMS traces of the nucleoside reaction mixtures after pyridination reaction conditions following general procedure B on dA. The MS shows the extracted mass spectrum of dA. From left to right: unknown product m/z=280 (2.1 min), dA (2.6 min), A base (3.0 min), dI (3.3 min).

5-hydroxymethyl-2'-deoxycytidine and 4-cyanopyridine under air

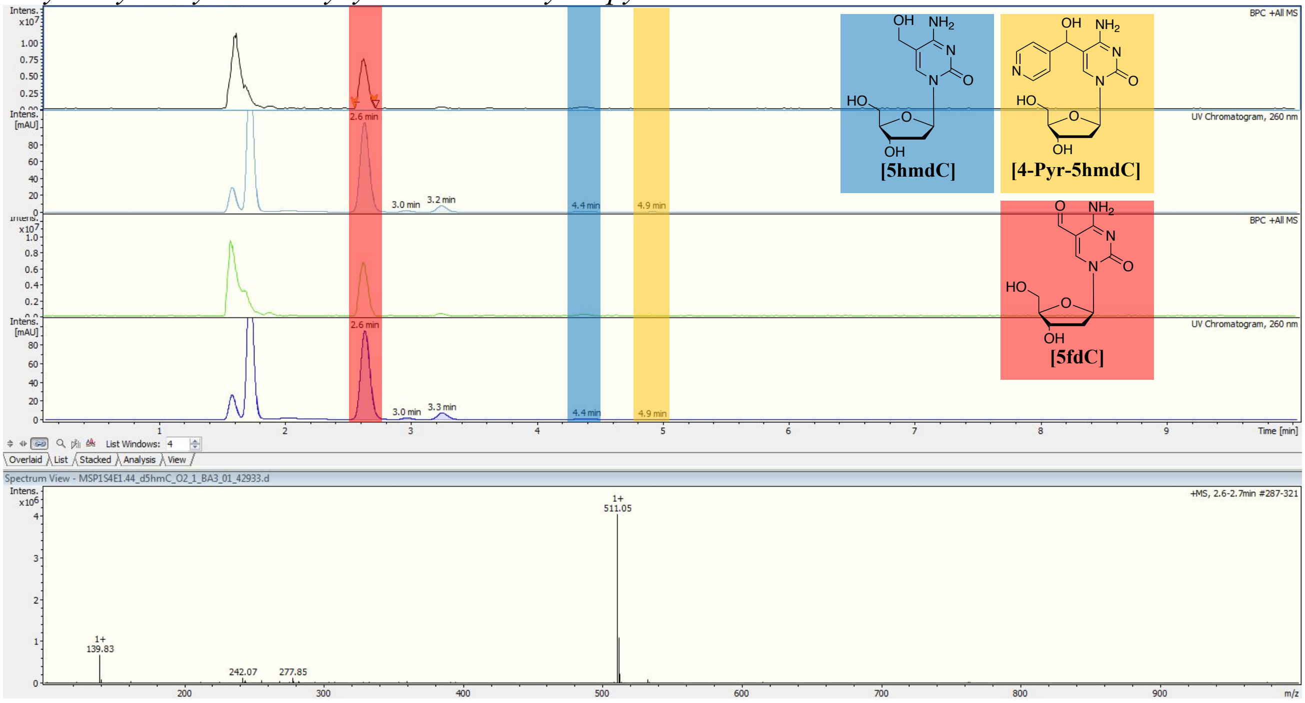

Figure S13: LCMS traces of the nucleoside reaction mixtures after pyridination reaction conditions following general procedure B on 5hmdC. The MS shows the extracted mass spectrum of the 5fdC. From left to right: 5fdC (2.6 min), 5fC base (3.0 min), dI (3.3 min), 4-Pyr-5hmdC (4.4 min), 5hmdC (4.9 min).

*5-methyl-2'-deoxycytidine and 4-cyanopyridine under argon **dark***

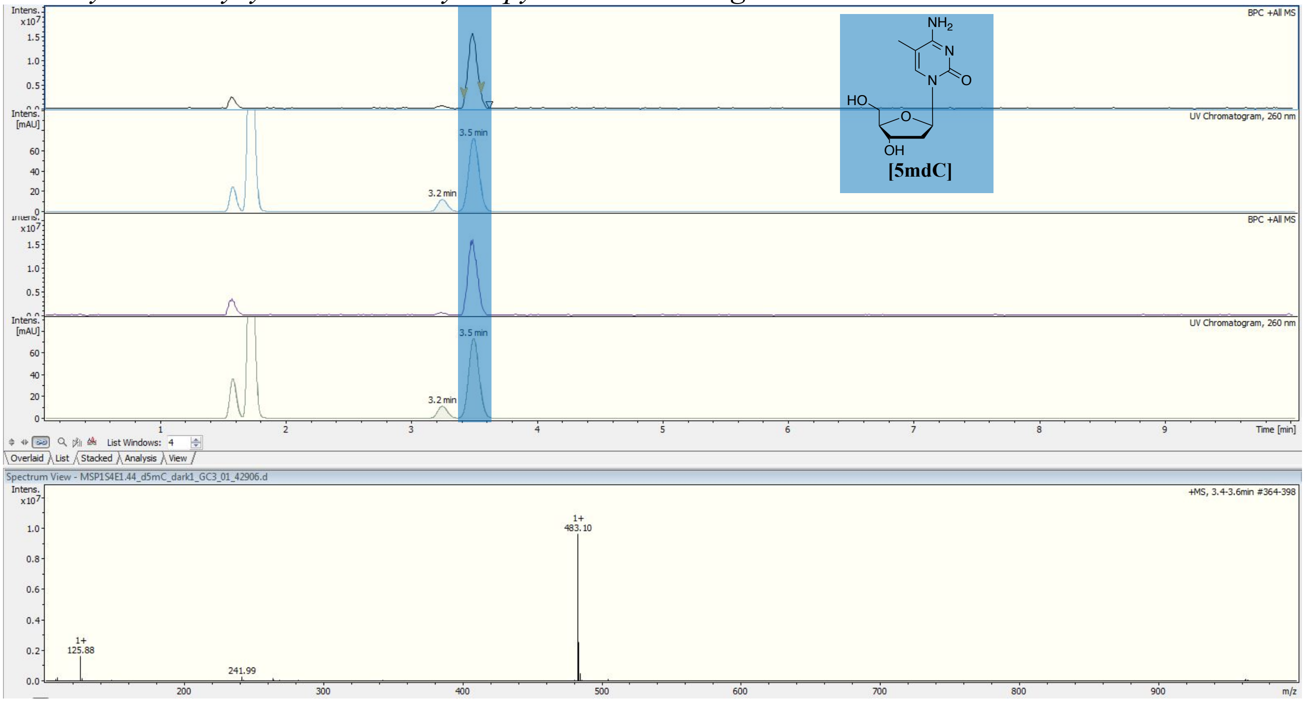

Figure S14: LCMS traces of the nucleoside reaction mixtures after pyridination reaction conditions following general procedure A on 5mdC except the reaction vial was wrapped in foil. The MS shows the extracted mass spectrum of the 5mdC. From left to right: dI (3.3 min), 5mdC (3.5 min).

Table S5: LCMS data of 5mdC pyridination reactions using different cyanopyridine derivatives

Cyanopyridine	Replicate	Time/h	dI	5mdC	dN/dI	Pyr	Pyr/dI
3-H	1	0	63.884	342.268	5.358	-	-
3-H	2	0	68.663	398.949	5.810	-	-
3-Me	1	0	63.954	446.322	6.979	-	-
3-Me	2	0	107.610	730.570	6.789	-	-
3-F	1	0	179.740	1088.940	6.058	-	-
3-F	2	0	74.743	508.439	6.802	-	-
3-OH	1	0	137.900	925.520	6.712	-	-
3-OH	2	0	87.607	590.328	6.738	-	-
3-Cl	1	0	72.563	468.030	6.450	-	-
3-Cl	2	0	82.411	547.081	6.638	-	-
3-NH2	1	0	61.529	463.774	7.537	-	-
3-NH2	2	0	41.928	302.686	7.219	-	-
3-CN	1	0	82.163	592.823	7.215	-	-
3-CN	2	0	69.952	503.780	7.202	-	-
3-Br	1	0	46.094	323.734	7.023	-	-
3-Br	2	0	41.200	323.344	7.848	-	-
3-H	1	20	64.916	34.147	0.526	147.208	2.268
3-H	2	20	62.603	16.832	0.269	150.765	2.408
3-Me	1	20	60.172	11.544	0.192	235.636	3.916
3-Me	2	20	60.245	3.542	0.059	241.176	4.003
3-F	1	20	97.468	352.355	3.615	146.900	1.507
3-F	2	20	82.721	342.308	4.138	127.536	1.542
3-OH	1	20	62.189	39.431	0.634	111.959	1.800
3-OH	2	20	60.669	34.360	0.566	112.780	1.859
3-Cl	1	20	81.854	349.216	4.266	117.583	1.436
3-Cl	2	20	67.355	334.077	4.960	76.309	1.133
3-NH2	1	20	72.972	509.021	6.976	0.000	0.000
3-NH2	2	20	62.780	443.046	7.057	0.000	0.000
3-CN	1	20	83.635	491.209	5.873	25.304	0.303
3-CN	2	20	80.333	476.330	5.929	25.665	0.319
3-Br	1	20	73.475	357.052	4.860	68.590	0.934
3-Br	2	20	45.382	226.388	4.989	56.422	1.243

Table S6: LCMS analysis of 5mdC pyridination reactions using different cyanopyridine derivatives

Cyanopyridine	Time/h	Average dN/dI	% Remaining	Average Pyr/dI	% Pyr
3-H	0	5.584	-	-	-
3-Me	0	6.884	-	-	-
3-F	0	6.430	-	-	-
3-OH	0	6.725	-	-	-
3-Cl	0	6.544	-	-	-
3-NH2	0	7.378	-	-	-
3-CN	0	7.209	-	-	-
3-Br	0	7.436	-	-	-
3-H	20	0.397	7.118	2.338	41.869
3-Me	20	0.125	1.820	3.960	57.520
3-F	20	3.877	60.285	1.524	23.707
3-OH	20	0.600	8.925	1.830	27.206
3-Cl	20	4.613	70.492	1.285	19.631
3-NH2	20	7.016	95.094	0.000	0.000
3-CN	20	5.901	81.867	0.311	4.315
3-Br	20	4.924	66.221	1.088	14.637

LCMS traces of 5mdC pyridination reactions using different cyanopyridine derivatives

This is a collection of LC traces of 5mdC pyridination reactions using different cyanopyridine derivatives as described in table S5. For figures S15-20: the first and second traces correspond to the base peak MS chromatogram and UV_{260} chromatogram of the first replicate respectively, the third and fourth traces correspond to the base peak MS chromatogram and UV_{260} chromatogram of the second replicate respectively.

3-methyl-4-cyanopyridine

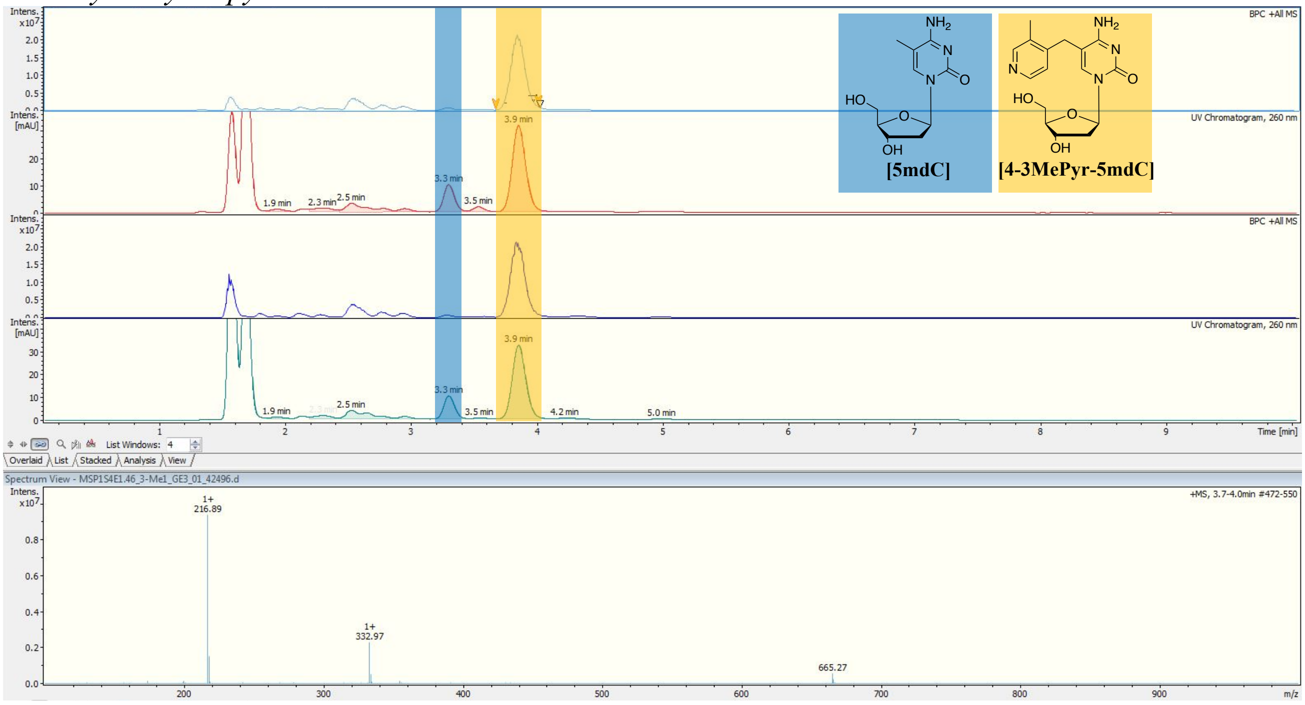

Figure S15: LCMS traces of the 5mdC reaction mixtures after pyridination reaction conditions following general procedure A with 3-methyl-4-cyanopyridine. The MS shows the extracted mass spectrum of the 3-methyl-4-cyanopyridine pyridination product. From left to right: unknown product of m/z 385 (1.9 min), unknown product of m/z 387 (2.3 min), small peaks corresponding to pre-HCN elimination product (m/z increase of +118, 2.5-3.0 min), dI (3.3 min), 5mdC (3.5 min), pyridination product (3.9 min).

3-fluoro-4-cyanopyridine

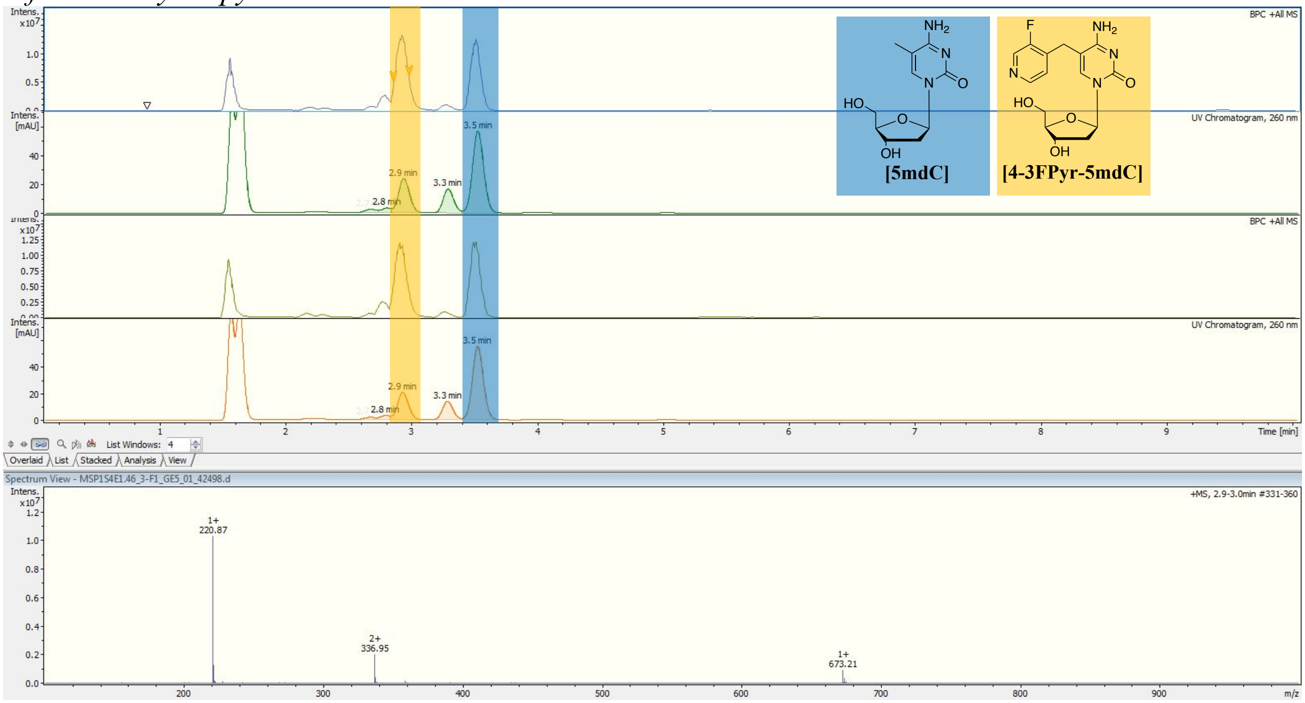

Figure S16: LCMS traces of the 5mdC reaction mixtures after pyridination reaction conditions following general procedure A with 3-fluoro-4-cyanopyridine. The MS shows the extracted mass spectrum of the 3-fluoro-4-cyanopyridine pyridination product. From left to right: small peak corresponding to pre-HCN elimination product (m/z increase of +122, 2.6 min), unknown product of m/z 344 (2.8 min), pyridination product (2.9 min), dI (3.3 min), 5mdC (3.5 min).

3-chloro-4-cyanopyridine

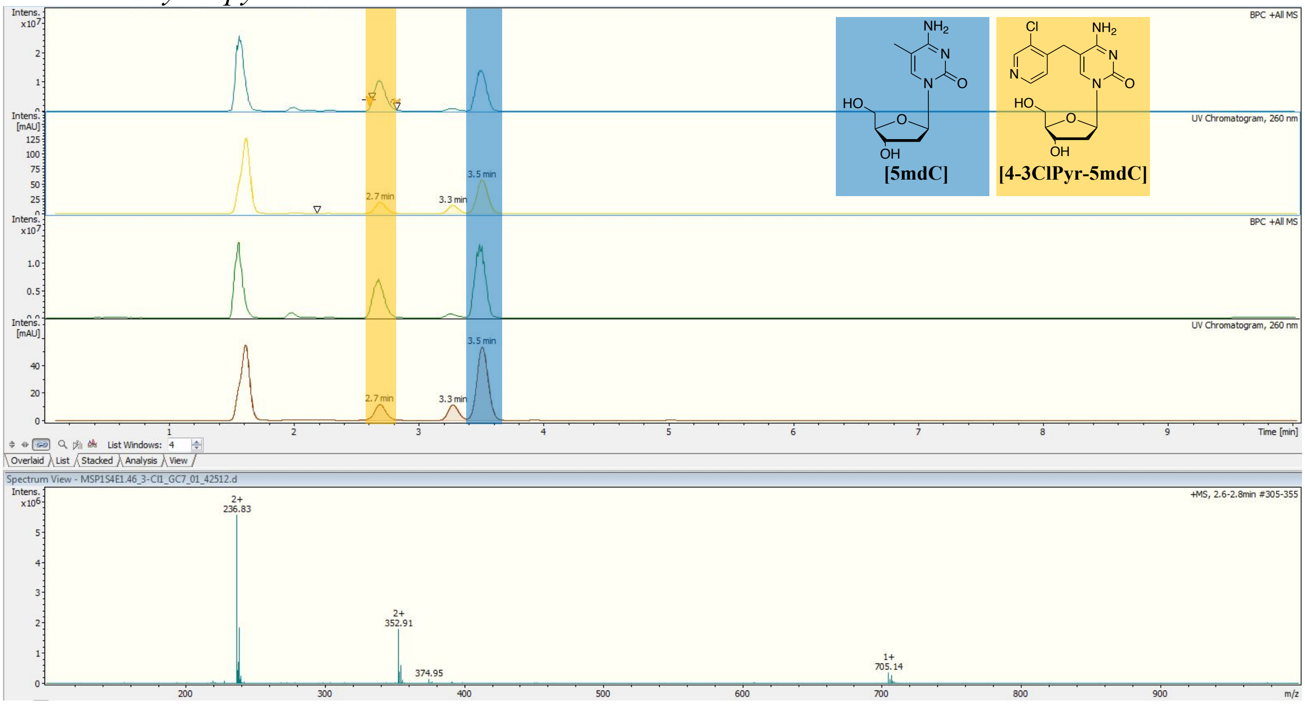

Figure S17: LCMS traces of the 5mdC reaction mixtures after pyridination reaction conditions following general procedure A with 3-chloro-4-cyanopyridine. The MS shows the extracted mass spectrum of the 3-chloro-4-cyanopyridine pyridination product. From left to right: pyridination product (2.7 min), dI (3.3 min), 5mdC (3.5 min).

3-bromo-4-cyanopyridine

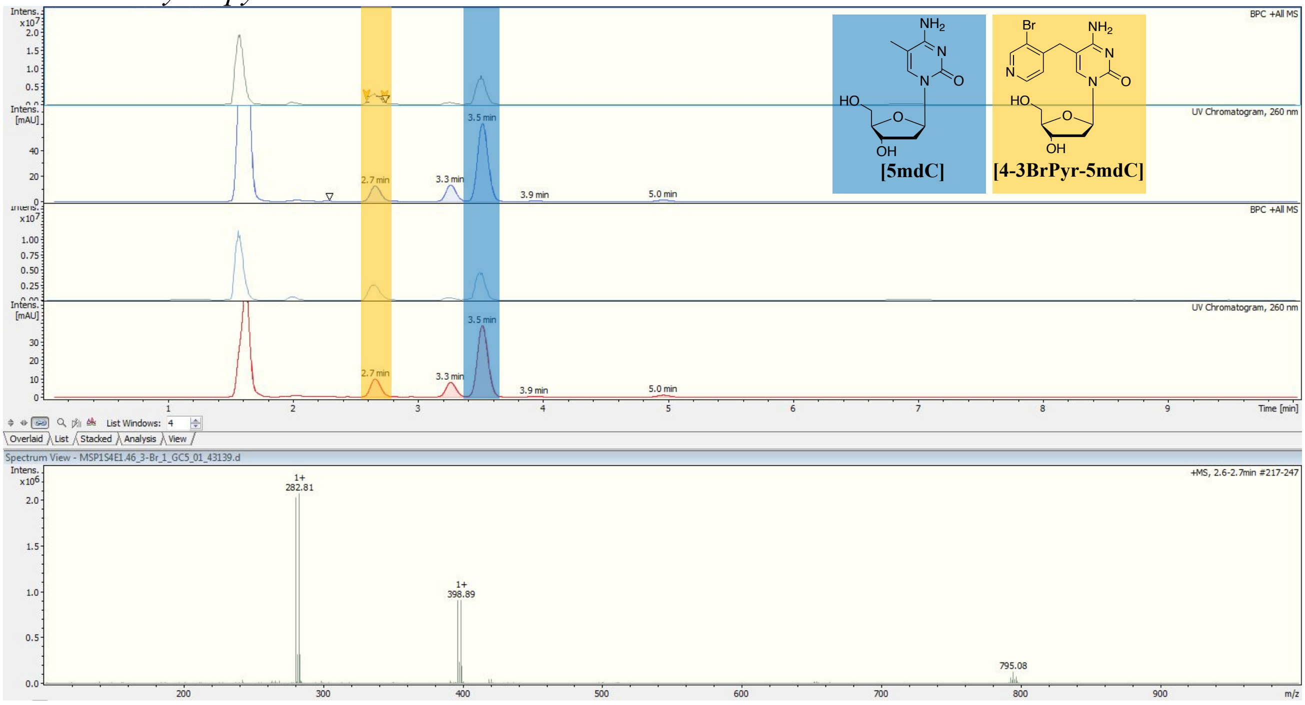

Figure S18: LCMS traces of the 5mdC reaction mixtures after pyridination reaction conditions following general procedure A with 3-bromo-4-cyanopyridine. The MS shows the extracted mass spectrum of the 3-bromo-4-cyanopyridine pyridination product. From left to right: pyridination product (2.7 min), dI (3.3 min), 5mdC (3.5 min), 5mC base (3.9 min), 5hmdC (5.0 min).

3-cyano-4-cyanopyridine

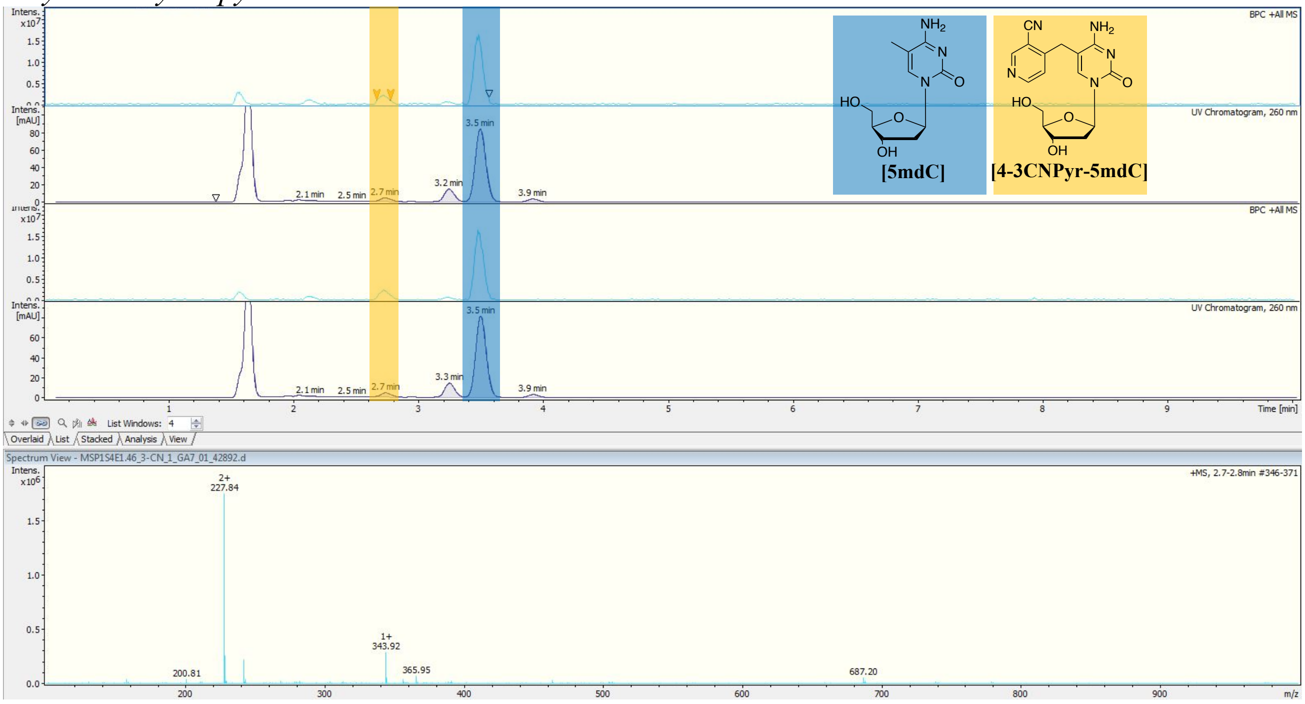

Figure S19: LCMS traces of the 5mdC reaction mixtures after pyridination reaction conditions following general procedure A with 3-cyano-4-cyanopyridine. The MS shows the extracted mass spectrum of the 3-cyano-4-cyanopyridine pyridination product. From left to right: Minisci reaction product (2.1 min), pre-HCN elimination product (2.5 min), pyridination product (2.7 min), dI (3.2/3.3 min), 5mdC (3.5 min), 5mC base (3.9 min).

3-hydroxy-4-cyanopyridine

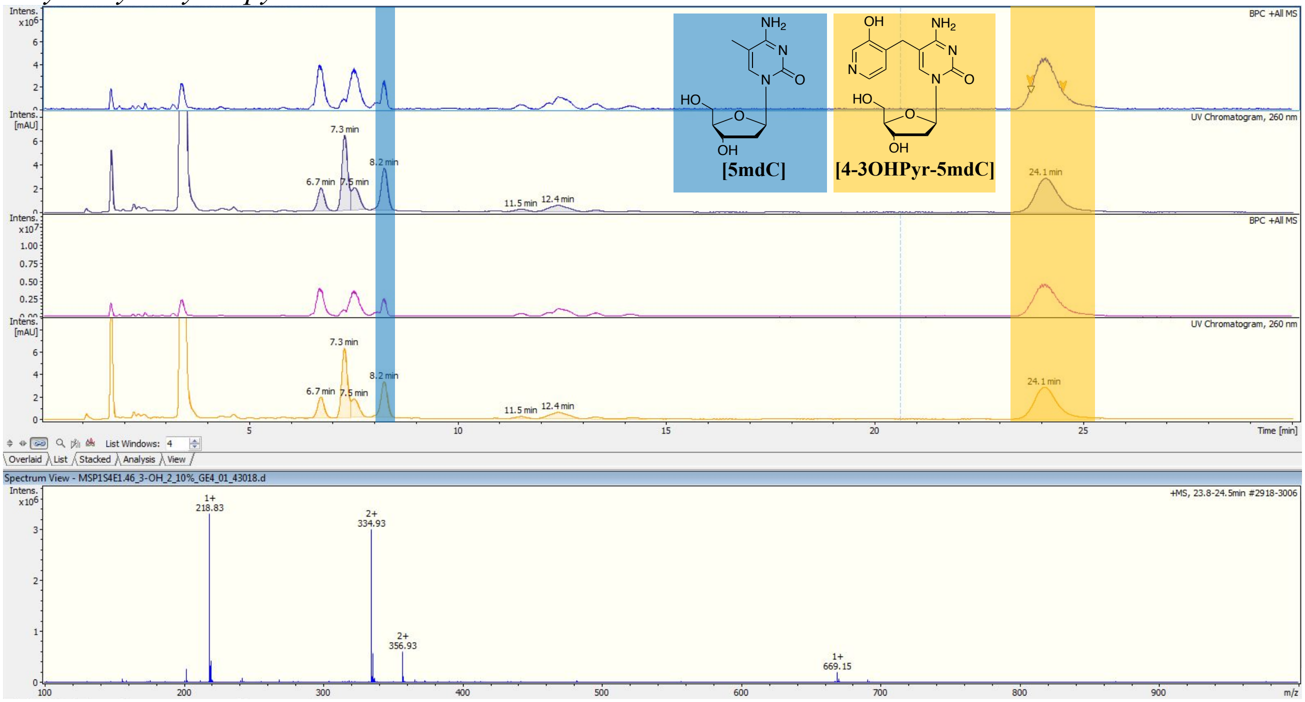

Figure S20: LCMS traces of the 5mdC reaction mixtures after pyridination reaction conditions following general procedure A with 3-hydroxy-4-cyanopyridine. An isocratic gradient of 90% MeCN vs. an aqueous solution of pH 3.7 10 mM NH_4OAc was used to achieve better separation. The MS shows the extracted mass spectrum of the 3-hydroxy-4-cyanopyridine pyridination product. From left to right: unknown product of m/z 389 (6.7 min), dI (7.3 min), Minisci reaction product (7.5 min), 5mdC (8.2 min), unknown product of m/z 482 (11.5 min), unknown product of m/z 389 (3 peaks, 12.4-14.2 min), pyridination product (24.1 min).

3-amino-4-cyanopyridine

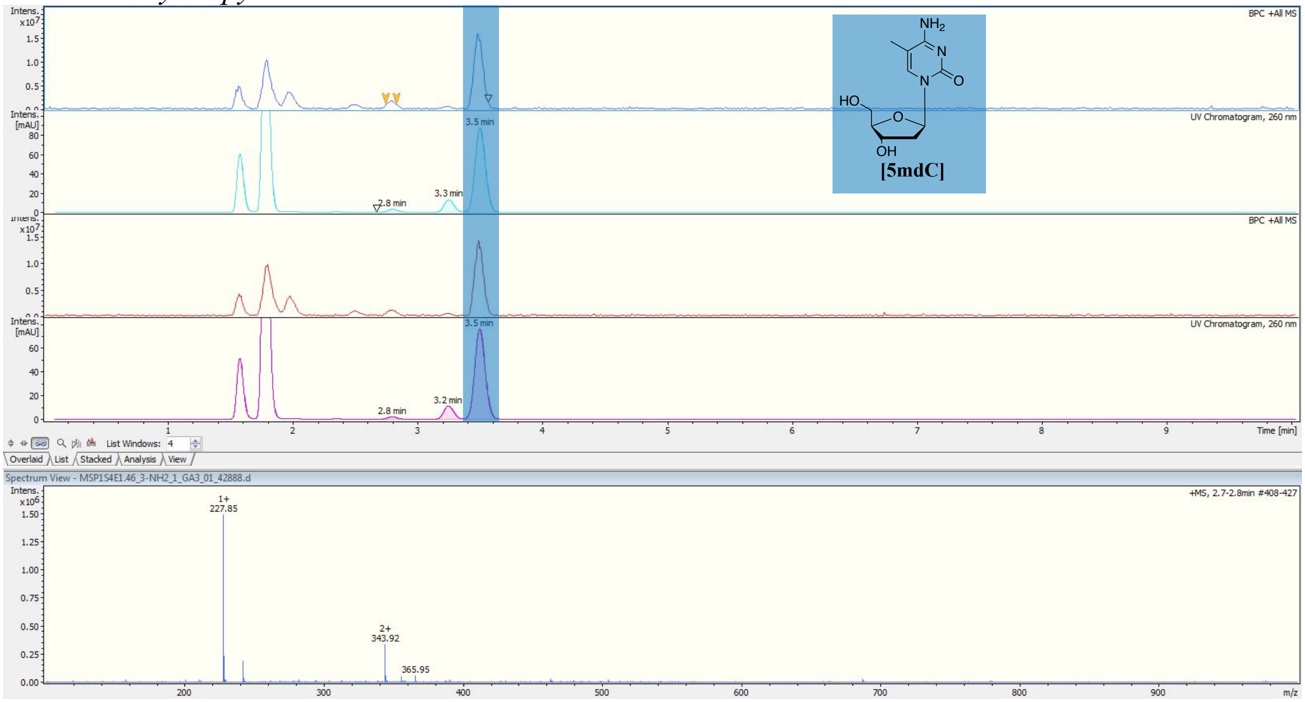

Figure S21: LCMS traces of the 5mdC reaction mixtures after pyridination reaction conditions following general procedure A with 3-amino-4-cyanopyridine. The MS shows the extracted mass spectrum of the unknown product of m/z 344. From left to right: unknown reaction product of m/z 344 (2.8 min), dI (3.2 min), 5mdC (3.5 min).

Section 3.2: Pyridination of Oligonucleotides

Oligonucleotide pyridination reactions were carried out according to general procedure C.

LCMS traces of oligonucleotide pyridination reactions using 4-cyanopyridine and xanthone

This is a collection of LC traces of oligonucleotide products following the pyridination reaction conditions. These LCMS spectra were recorded on an Amazon X ESI-MS (Bruker) connected to an Ultimate 3000 LC (Dionex). For figures S21-S23 the first and second traces correspond to the base peak MS chromatogram and UV_{260} chromatogram of the oligonucleotide pyridination reaction products. The MS shows the extracted mass spectrum of the methylcytosine pyridination product peak.

5mC 12mer

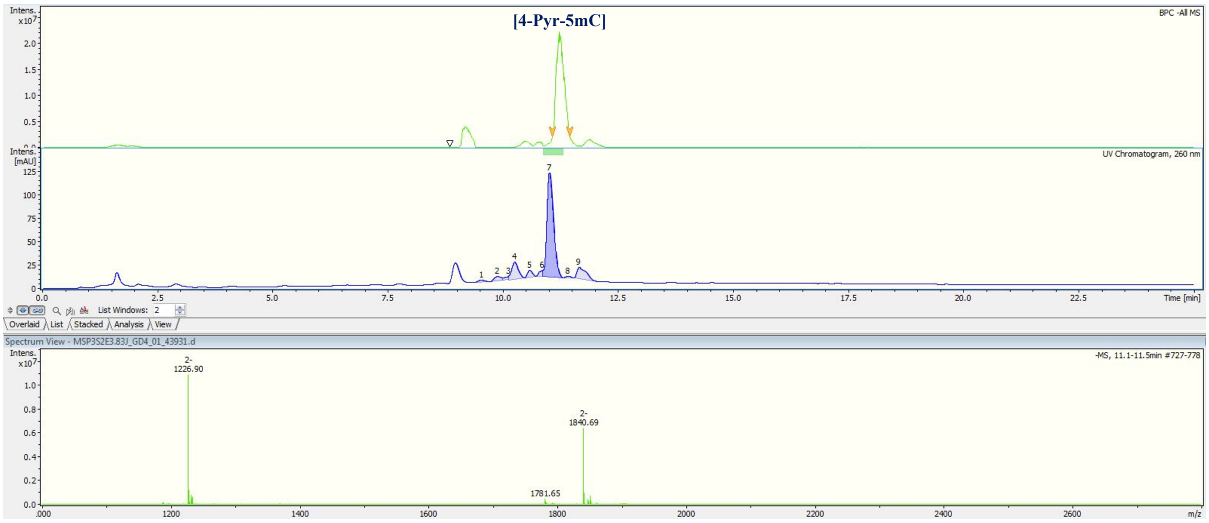

Figure S22: LCMS traces of the 5mC 12mer pyridination products.

5mC 10mer

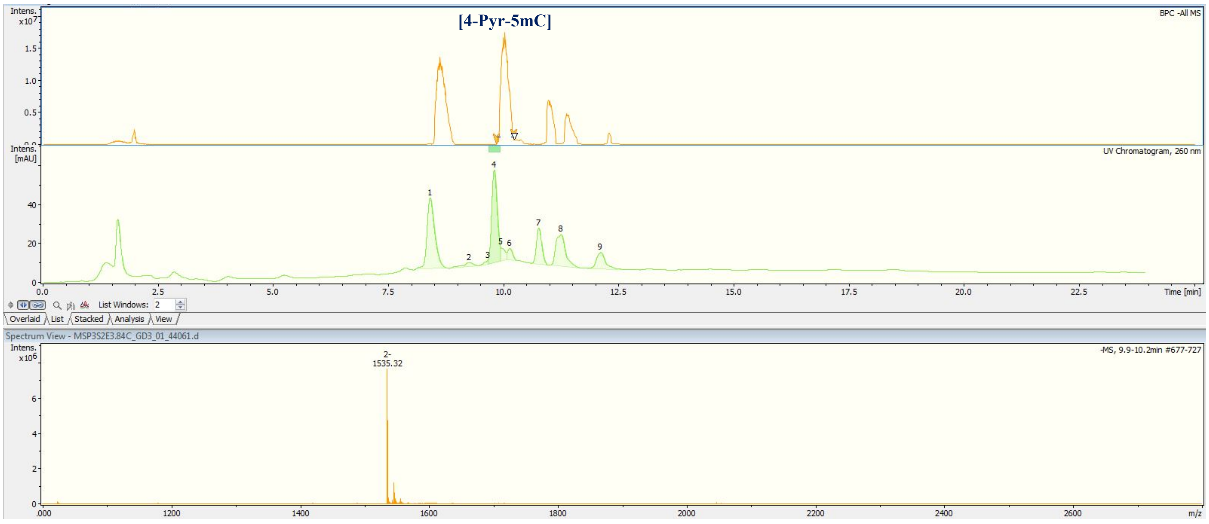

Figure S23: LCMS traces of the 5mC 10mer pyridination products.

m5C 10mer

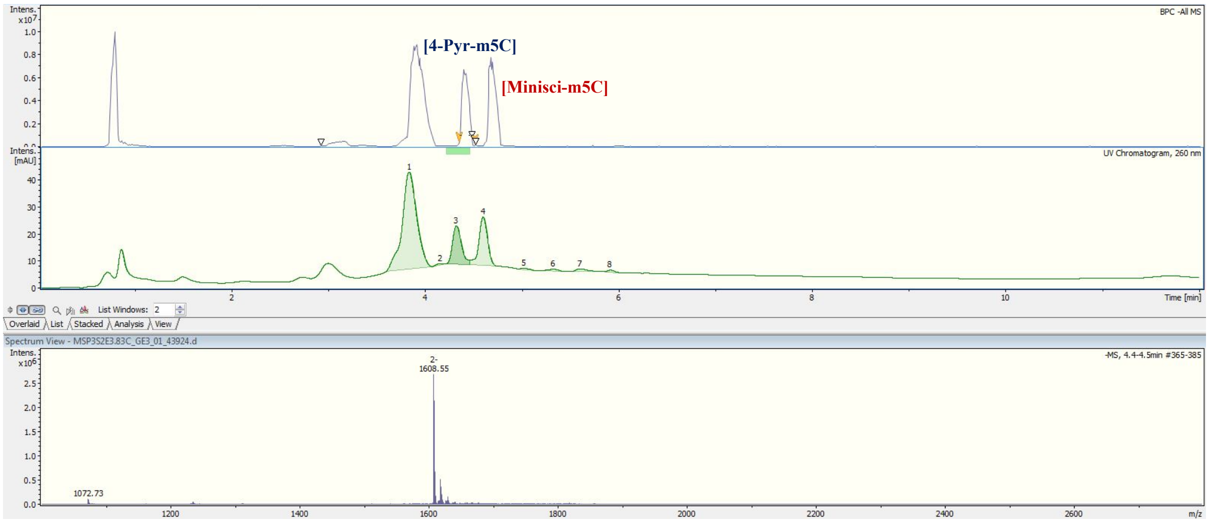

Figure S24: LCMS traces of the m5C 10mer pyridination products.

54mer Degradase Single Nucleoside Product Analysis

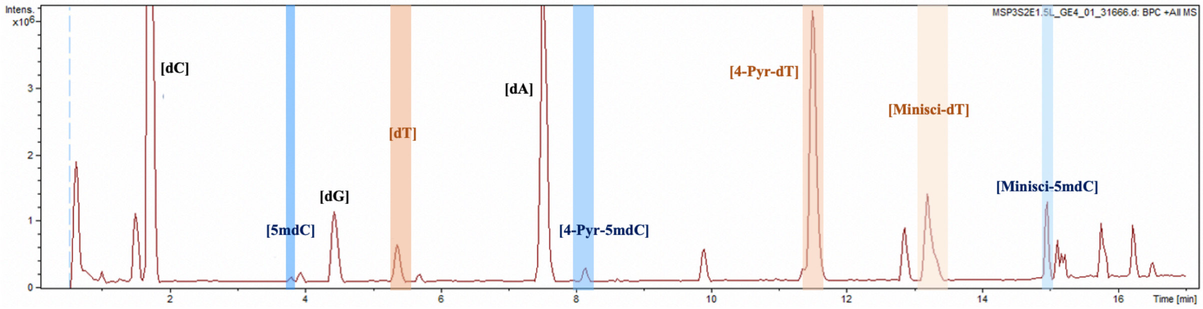

Figure S25: Base peak MS chromatogram of the single nucleoside reaction products following treatment of the 54mer oligonucleotide pyridination products with DNA degradase plus. Nucleoside peaks were identified by a characteristic -116 m/z corresponding to loss of deoxyribose. The following nucleoside pyridination product peaks were identified: 319.29 (4-Pyr-5mdC, 5mdC+77), 320.31 (4-Pyr-dT, dT+77), 345.30 (Minisci-dT, dT+102), 344.43 (Minisci-5mdC, 5mdC+102). All unlabelled peaks do not correspond to nucleoside peaks and are believed to be either column contaminants or DNA degradase plus components. As a short ssDNA 54mer is not a preferred substrate for DNA degradase, results are not quantitative.

Table S7: List of base modifications used for BioPharma Finder 4.1 oligonucleotide analysis

Base	Letter Code	Molecular Formula	Chemical Structure
Adenine	A	$C_5H_5N_5$	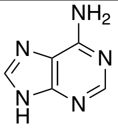
Guanine	G	$C_5H_5N_5O$	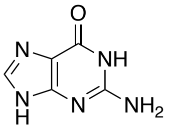
Cytosine	C	$C_4H_5N_3O$	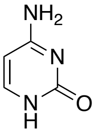
Thymine	T	$C_5H_6N_2O_2$	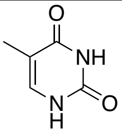
Uracil	U	$C_4H_4N_2O_2$	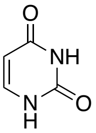
Methylcytosine	M	$C_5H_7N_3O$	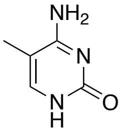
Formylcytosine	F	$C_5H_5N_3O_2$	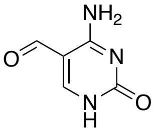
4-Pyr-5mC	V	$C_{10}H_{10}N_4O$	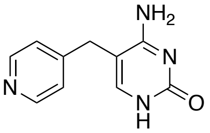
Minisci-5mC	B	$C_{11}H_9N_5O$	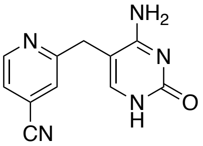
4-Pyr-T	Y	$C_{10}H_9N_3O_2$	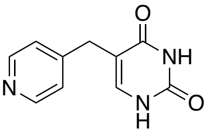
Minisci-T	W	$C_{11}H_8N_4O_2$	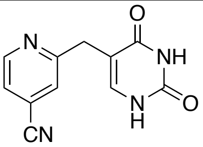

Section 3.2.1. 5mC 12mer Pyridination Product Analysis

Table S8: 5mC 12mer Oligonucleotide Product Summary

Oligonucleotide	Description	Sequence
1	Starting oligonucleotide	AGACCAMAACCA
2	Pyridination at 5mC	AGACCAVAACCA
3	Minisci addition at 5mC	AGACCABAACCA
4	Oxidation of 5mC to 5fC	AGACCAFAACCA

Oligonucleotide	Number of MS Peaks	MS Peak Area	Sequence Coverage	Abundance (mol)
1:1	2	8.7%	100.0%	11.68%
1:1*	2	8.7%	0.0%	11.68%
2:2	4	48.4%	100.0%	64.82%
2:2*	4	48.4%	0.0%	64.82%
3:3	2	11.0%	100.0%	14.75%
3:3*	2	11.0%	0.0%	14.75%
4:4	2	6.5%	100.0%	8.752%
4:4*	2	6.5%	0.0%	8.752%
Unidentified	7	25.4%		

Minimum Recovery = 1%
Minimum Recovery of Overlapping Oligonucleotides = 0%
Minimum Confidence = 0.5
Maximum Mass = 10000

Color code for oligonucleotide recovery

>50.0%	>20.0%	>10.0%	>5.0%	>2.0%	>1.0%	>0.5%	>0.2%	>0.1%	>0.0%
good			fair			poor			

1

1 2 3 4 5 6 7 8 9 10 11 12
Ad- pGd- pAd- pCd- pCd- pAd- pMd- pAd- pAd- pCd- pCd- pAd
8.8

2

1 2 3 4 5 6 7 8 9 10 11 12
Ad- pGd- pAd- pCd- pCd- pAd- pVd- pAd- pAd- pCd- pCd- pAd
9.5

3

1 2 3 4 5 6 7 8 9 10 11 12
Ad- pGd- pAd- pCd- pCd- pAd- pBd- pAd- pAd- pCd- pCd- pAd
10.1

4

1 2 3 4 5 6 7 8 9 10 11 12
Ad- pGd- pAd- pCd- pCd- pAd- pFd- pAd- pAd- pCd- pCd- pAd
8.9

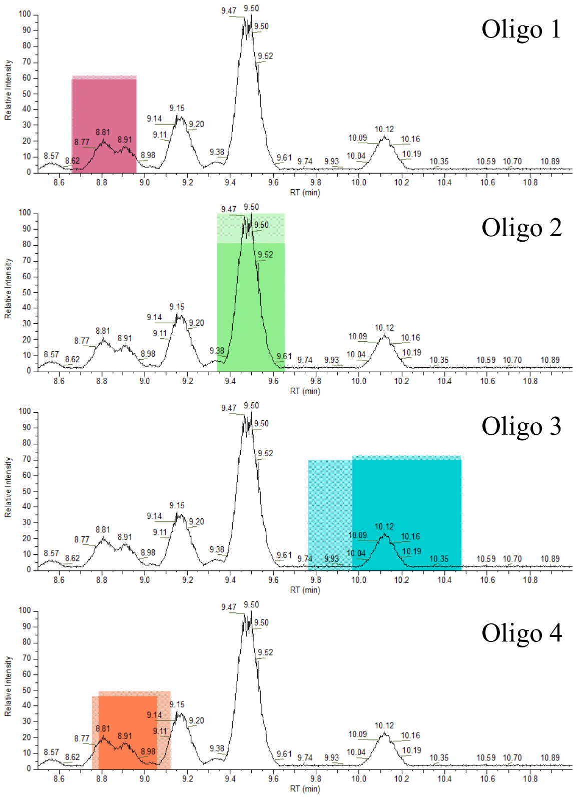

Figure S26: Oligonucleotide sequence coverage map and highlighted chromatogram of 5mC 12mer oligonucleotide pyridination products. High-resolution mass spectra (HRMS) of oligonucleotides were recorded on an Orbitrap Exploris 120 (Thermo Scientific™) connected to Vanquish HPLC system (Thermo Scientific™).

Section 3.2.2. 5mC 10mer Pyridination Product Analysis

Table S9: 5mC 10mer Oligonucleotide Product Summary

Oligonucleotide	Description	Sequence
1	Starting oligonucleotide	AGACCTACMA
2	Pyridination at 5mC	AGACCTACVA
3	Pyridination at T	AGACCYACMA
4	Pyridination at T and 5mC	AGACCYACVA
5	Minisci addition at T	AGACCWACMA
6	Minisci addition at 5mC	AGACCTACBA
7	Minisci addition at T and 5mC	AGACCWACBA
8	Pyridination at T, Minisci addition at 5mC	AGACCYACBA
9	Minisci addition at T, pyridination at 5mC	AGACCWACVA
10	Oxidation of 5mC to 5fC	AGACCTACFA

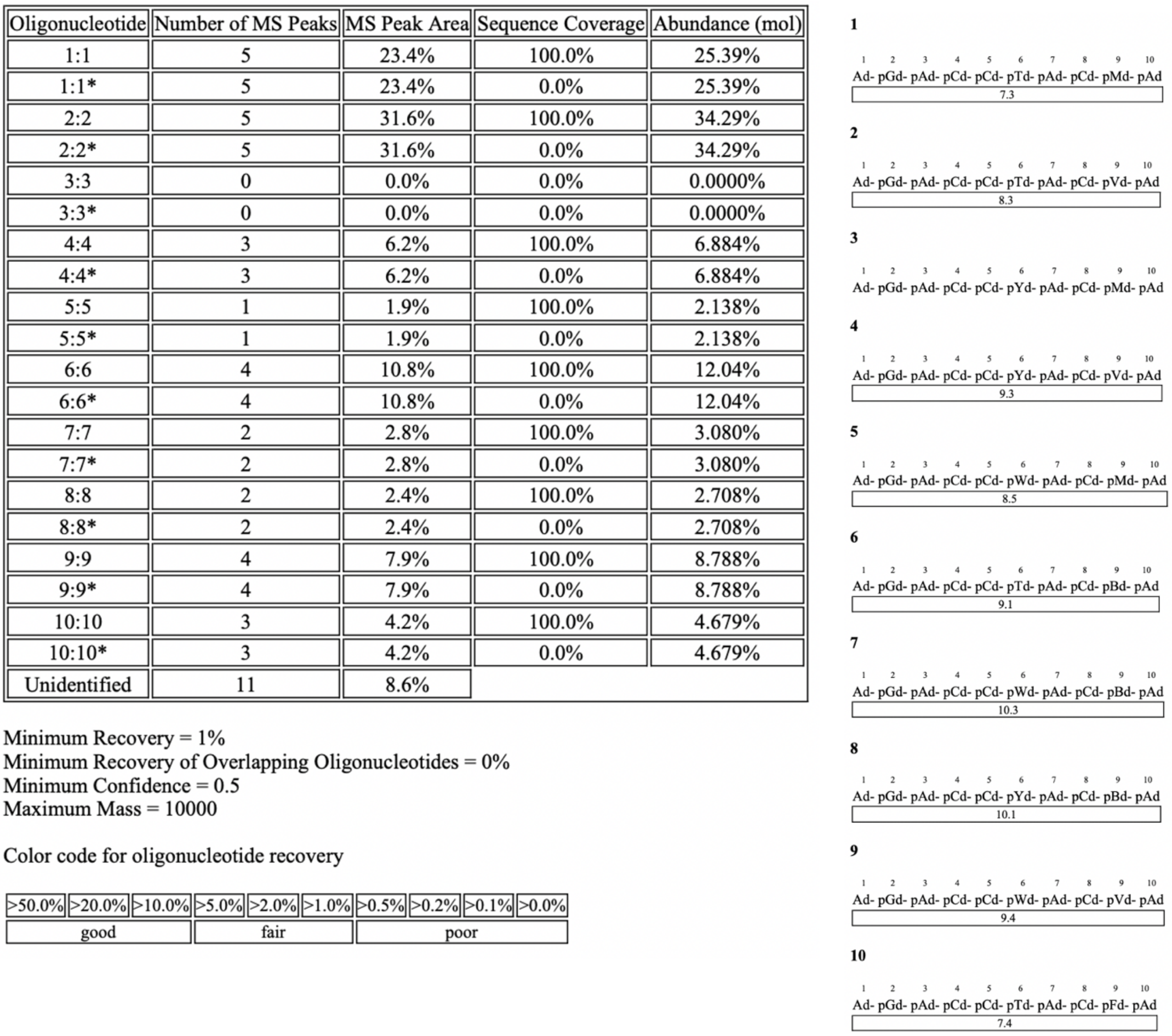

Oligonucleotide	Number of MS Peaks	MS Peak Area	Sequence Coverage	Abundance (mol)
1:1	5	23.4%	100.0%	25.39%
1:1*	5	23.4%	0.0%	25.39%
2:2	5	31.6%	100.0%	34.29%
2:2*	5	31.6%	0.0%	34.29%
3:3	0	0.0%	0.0%	0.0000%
3:3*	0	0.0%	0.0%	0.0000%
4:4	3	6.2%	100.0%	6.884%
4:4*	3	6.2%	0.0%	6.884%
5:5	1	1.9%	100.0%	2.138%
5:5*	1	1.9%	0.0%	2.138%
6:6	4	10.8%	100.0%	12.04%
6:6*	4	10.8%	0.0%	12.04%
7:7	2	2.8%	100.0%	3.080%
7:7*	2	2.8%	0.0%	3.080%
8:8	2	2.4%	100.0%	2.708%
8:8*	2	2.4%	0.0%	2.708%
9:9	4	7.9%	100.0%	8.788%
9:9*	4	7.9%	0.0%	8.788%
10:10	3	4.2%	100.0%	4.679%
10:10*	3	4.2%	0.0%	4.679%
Unidentified	11	8.6%		

Figure S27: Oligonucleotide sequence coverage map of 5mC 10mer oligonucleotide pyridination products.

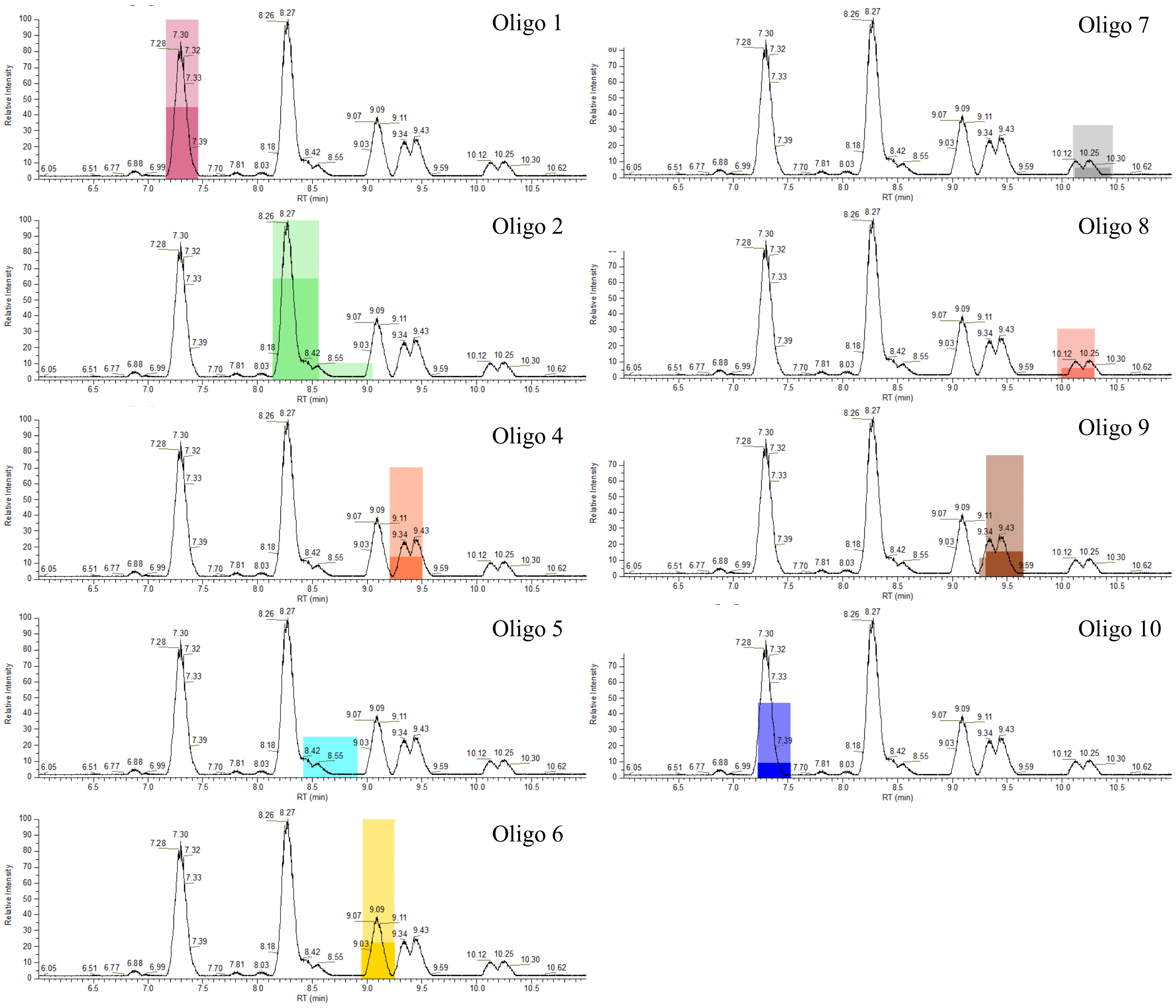

Figure S28: Highlighted chromatogram of 5mC 10mer oligonucleotide pyridination products. High-resolution mass spectra (HRMS) of oligonucleotides were recorded on an Orbitrap Exploris 120 (Thermo Scientific™) connected to Vanquish HPLC system (Thermo Scientific™).

Section 3.2.3. m5C 10mer Pyridination Product Analysis

Table S10: 5mC 12mer Oligonucleotide Product Summary

Oligonucleotide	Description	Sequence
1	Starting oligonucleotide	AGACCUACMA
2	Pyridination at 5mC	AGACCUACVA
3	Minisci addition at 5mC	AGACCUACBA
4	Oxidation of 5mC to 5fC	AGACCUACFA

Oligonucleotide	Number of MS Peaks	MS Peak Area	Sequence Coverage	Abundance (mol)
1:1	4	35.5%	100.0%	35.51%
1:1*	4	35.5%	0.0%	35.51%
2:2	4	18.1%	100.0%	18.13%
2:2*	4	18.1%	0.0%	18.13%
3:3	4	19.2%	100.0%	19.17%
3:3*	4	19.2%	0.0%	19.17%
4:4	4	27.2%	100.0%	27.20%
4:4*	4	27.2%	0.0%	27.20%
Unidentified	0	0.0%		

Minimum Recovery = 1%
Minimum Recovery of Overlapping Oligonucleotides = 0%
Minimum Confidence = 0.5
Maximum Mass = 10000

Color code for oligonucleotide recovery

>50.0%	>20.0%	>10.0%	>5.0%	>2.0%	>1.0%	>0.5%	>0.2%	>0.1%	>0.0%
good			fair			poor			

1

1 2 3 4 5 6 7 8 9 10
A - pG - pA - pC - pC - pU - pA - pC - pM - pA
5.6

2

1 2 3 4 5 6 7 8 9 10
A - pG - pA - pC - pC - pU - pA - pC - pV - pA
7.1

3

1 2 3 4 5 6 7 8 9 10
A - pG - pA - pC - pC - pU - pA - pC - pB - pA
7.8

4

1 2 3 4 5 6 7 8 9 10
A - pG - pA - pC - pC - pU - pA - pC - pF - pA
5.7

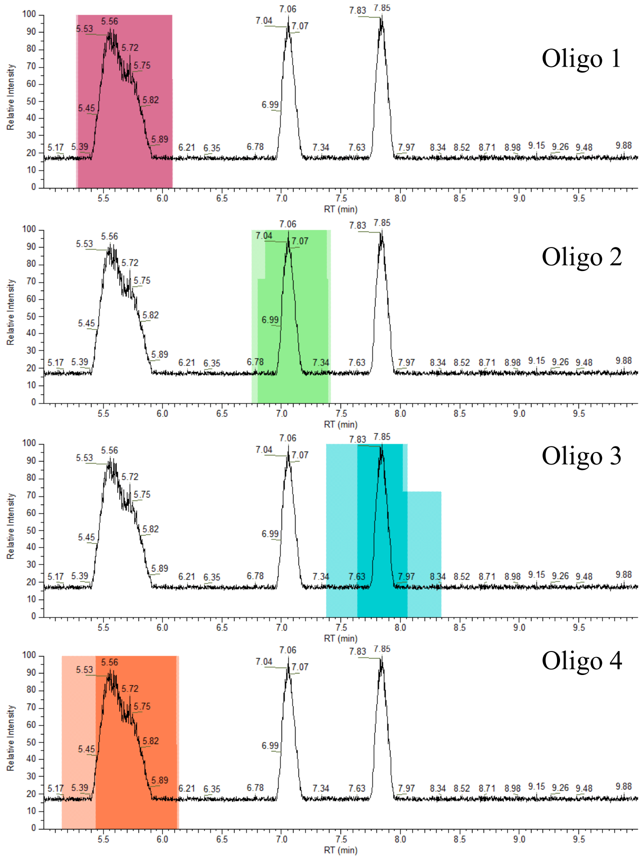

Figure S29: Oligonucleotide sequence coverage map and highlighted chromatogram of m5C 10mer oligonucleotide pyridination products. High-resolution mass spectra (HRMS) of oligonucleotides were recorded on an Orbitrap Exploris 120 (Thermo Scientific™) connected to Vanquish HPLC system (Thermo Scientific™).

Section 4. HaloTag-Based Enrichment Studies

Section 4.1. Synthesis of Chloroalkane-Cyanopyridine

2-(2-((6-chlorohexyl)oxy)ethoxy)ethan-1-amine

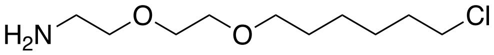

A 60% dispersion of NaH in mineral oil (1.80 g, 45 mmol, 1.5 equiv.) was dissolved in dry heptane (10 mL) under argon. The flask was swirled then the heptane was carefully decanted. The process was repeated 3 more times, then the remaining solid was dried under high vacuum for 30 minutes. The dry sodium hydride was suspended in dry DMF (30 mL) under argon. The flask was cooled to 0°C then 2-(2-aminoethoxy)ethanol (3.0 mL, 30 mmol, 1,0 equiv.) was added slowly via syringe. The reaction mixture was stirred at 0°C for 45 minutes. 1-chloro-6-iodohexane (4.5 mL, 39 mmol, 1.0 equiv.) in dry DMF (20 mL) was slowly added to the reaction. The reaction mixture was stirred for a further 2.5 hours at 0°C. The suspension was filtered through celite then DMF was removed via GeneVac (high bp, 90 mins, max temp 40°C). The residue was dissolved in DCM (50 mL) then washed with saturated sodium bicarbonate (50 mL) and water (2 x 50 mL). The organic layer was dried over $MgSO_4$, filtered and the solvent was removed in vacuo (max temp 30°C). The oil was cooled in a bath of dry ice and isopropanol and left under high vacuum for 3 hours. The 2-[2-(6-chloro-hexyloxy)-ethoxy]-ethylamine was dissolved in DCM (10 ml) and the reaction flask cooled in an ice bath. Diethyl ether saturated with hydrogen chloride gas (25 ml) was added and the reaction stirred for 2 hours at 0 °C. The solvent was removed in vacuo to give a yellow oil (~1.7g). The product was purified by flash column chromatography (0-10% 1% ether.HCl-methanol/1% ether.HCl-DCM) to give 2-(2-((6-chlorohexyl)oxy)ethoxy)ethan-1-amine.HCl as a yellow solid (1.94 g, 7.47 mmol, 25 %).

^{1}H NMR (400 MHz, Chloroform-*d*) δ 3.84 (t, *J* = 5.1 Hz, 1H), 3.74 – 3.66 (m, 1H), 3.66 – 3.60 (m, 1H), 3.56 (t, *J* = 6.7 Hz, 1H), 3.50 (t, *J* = 6.8 Hz, 1H), 3.25 (t, *J* = 5.1 Hz, 1H), 1.86 – 1.75 (m, 1H), 1.62 (q, *J* = 7.1 Hz, 1H), 1.54 – 1.43 (m, 1H), 1.39 (dddd, *J* = 14.3, 9.0, 6.3, 3.4 Hz, 1H).
^{13}C NMR (101 MHz, Chloroform-*d*) δ 71.33, 70.41, 69.92, 66.73, 45.09, 39.75, 32.51, 29.34, 26.67, 25.36.
HMRS-ESI (m/z): found $[M+H]^+$ 224.1421, C10H23ClNO2 requires 224.14173

***tert*-butyl (28-chloro-15-oxo-3,6,9,12,19,22-hexaoxa-16-azaoctacosyl)carbamate**

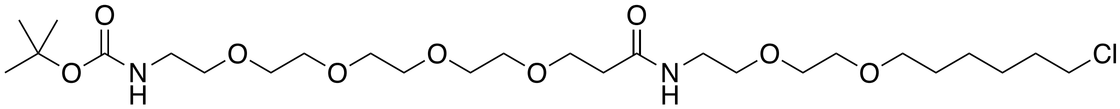

To a solution of 2,2-dimethyl-4-oxo-3,8,11,14,17-pentaoxa-5-azaicosan-20-oic acid (428 mg, 1.17 mmol, 1.0 equiv.) and 2-(2-((6-chlorohexyl)oxy)ethoxy)ethan-1-amine.HCl (458 mg, 1.76 mmol, 1.5 equiv.) in dry DCM (20 mL) was added DIPEA (918 µL, 5.27 mmol, 4.5 equiv.) and the mixture was stirred at room temperature for 15 minutes. HATU (669 mg, 1.76 mmol, 1.5 equiv.) was then added and the mixture was stirred at room temperature for 1 hour. The reaction mixture was diluted with DCM (150 mL) and washed with aqueous potassium carbonate (3 x 60 mL), aqueous saturated NH_4Cl (2 x 60 mL) and brine (60 mL), dried over $MgSO_4$, filtered, and the solvent was removed in vacuo. The residue was purified by flash column chromatography (0-15% MeOH/DCM) to yield *tert*-butyl (28-chloro-15-oxo-3,6,9,12,19,22-hexaoxa-16-azaoctacosyl)carbamate as a colourless oil (542.4 mg, 0.95 mmol, 81 %).

^{1}H NMR (400 MHz, Chloroform-*d*) δ 6.65 (s, 1H), 5.30 (s, 1H), 5.14 (t, *J* = 5.8 Hz, 1H), 3.74 (t, *J* = 5.9 Hz, 2H), 3.67 – 3.60 (m, 12H), 3.59 (ddd, *J* = 14.9, 4.4, 2.0 Hz, 5H), 3.58 – 3.40 (m, 10H), 3.31 (q, *J* = 5.4 Hz, 2H), 2.48 (t, *J* = 5.9 Hz, 2H), 2.24 (s, 1H), 1.83 – 1.72 (m, 2H), 1.60 (p, *J* = 6.8 Hz, 2H), 1.46 (s, 1H), 1.43 – 1.31 (m, 2H).
^{13}C NMR (101 MHz, Chloroform-*d*) δ 171.50, 79.19, 71.24, 70.48, 70.46, 70.39, 70.30, 70.25, 70.19, 70.11, 70.01, 69.77, 67.26, 45.03, 39.14, 36.90, 32.51, 29.44, 28.42, 26.67, 25.40.
HMRS-ESI (m/z): found $[M+H]^+$ 571.3354, C26H52ClN2O9 requires 571.33613.

1-amino-*N*-(2-(2-((6-chlorohexyl)oxy)ethoxy)ethyl)-3,6,9,12-tetraoxapentadecan-15-amide

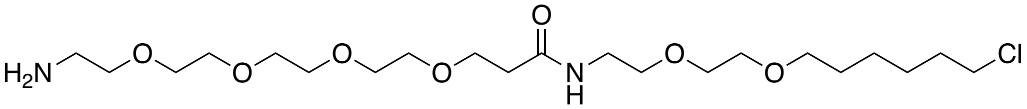

To a solution of *tert*-butyl (28-chloro-15-oxo-3,6,9,12,19,22-hexaoxa-16-azaoctacosyl)carbamate (420 mg, 0.75 mmol) in DCM (8 mL), TFA (2 mL) was added, and the mixture was stirred at room temperature for 1 hour. The solvent was removed in vacuo then the residue was redissolved in $NaHCO_3$ (5 mL) then extracted with DCM (3 x 10 mL). The combined organic layers were washed with brine (15 mL), dried over $MgSO_4$, filtered, and the solvent was removed in vacuo to give 1-amino-*N*-(2-(2-((6-chlorohexyl)oxy)ethoxy)ethyl)-3,6,9,12-tetraoxapentadecan-15-amide as a colourless oil (308.5 mg, 0.68 mmol, 90 %).

^{1}H NMR (400 MHz, Chloroform-*d*) δ 8.14 (s, 2H), 7.37 (d, *J* = 6.7 Hz, 1H), 3.94 (s, 1H), 3.84 – 3.78 (m, 1H), 3.75 (t, *J* = 5.4 Hz, 1H), 3.73 – 3.51 (m, 11H), 3.51 – 3.39 (m, 3H), 3.21 (s, 1H), 2.53 (t, *J* = 5.6 Hz, 1H), 1.78 (p, *J* = 6.8 Hz, 1H), 1.61 (p, *J* = 6.9 Hz, 1H), 1.51 – 1.30 (m, 3H).

^{13}C NMR (101 MHz, Chloroform-*d*) δ 71.19, 70.03, 69.97, 69.93, 69.86, 69.80, 69.60, 67.31, 67.19, 45.05, 39.55, 39.13, 36.04, 32.48, 29.29, 26.64, 25.30.

HMRS-ESI (m/z): found $[M+H]^+$ 471.2838, C21H44ClN2O7 requires 471.2837.

HaloLig-CP
[*N*-(28-chloro-15-oxo-3,6,9,12,19,22-hexaoxa-16-azaoctacosyl)-4-cyanopicolinamide]

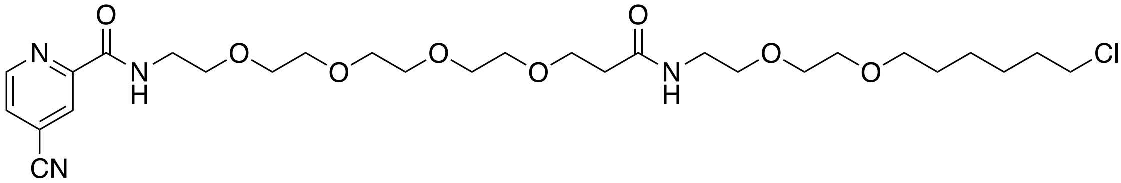

Lithium 4-cyanopyridine-2-carboxylate (129 mg, 0.84 mmol, 1.5 equiv.), DIPEA (146 µL, 0.84 mmol, 1.5 equiv.) and HATU (319 mg, 0.84 mmol, 1.5 equiv.) were dissolved in dry DMF (6 mL). A solution of 1-amino-*N*-(2-(2-((6-chlorohexyl)oxy)ethoxy)ethyl)-3,6,9,12-tetraoxapentadecan-15-amide (264 mg, 0.56 mmol, 1.0 equiv.) and DIPEA (292 µL, 2.51 mmol, 3.0 equiv.) dissolved in dry DMF (5 mL) was slowly added to the reaction mixture. The reaction mixture was stirred at room temperature for 20 hours. The mixture was diluted with DCM (120 mL) and washed with aqueous potassium carbonate (4 x 50 mL), aqueous saturated NH_4Cl (4 x 50 mL) and brine (50 mL), dried over $MgSO_4$, filtered, and the solvent was removed in vacuo. The residue was purified by flash column chromatography (0-20% MeOH/DCM) to give HaloLig-CP as a yellow oil (253 mg, 0.42 mmol, 75 %).

^{1}H NMR (400 MHz, Chloroform-*d*) δ 8.75 (dd, *J* = 4.9, 0.9 Hz, 1H), 8.39 (t, *J* = 1.2 Hz, 1H), 8.29 (s, 1H), 7.67 (dd, *J* = 5.0, 1.6 Hz, 1H), 6.60 (t, *J* = 5.7 Hz, 1H), 3.76 – 3.48 (m, 25H), 3.44 (dd, *J* = 7.5, 5.8 Hz, 4H), 2.45 (t, *J* = 6.0 Hz, 2H), 1.81 – 1.70 (m, 2H), 1.58 (p, *J* = 6.9 Hz, 2H), 1.50 – 1.29 (m, 3H), 1.39 (s, 1H).

^{13}C NMR (101 MHz, Chloroform-*d*) δ 171.37, 162.54, 149.19, 127.50, 124.09, 115.97, 77.31, 71.22, 70.53, 70.49, 70.46, 70.34, 70.30, 70.24, 70.22, 70.00, 69.75, 69.72, 67.24, 45.03, 39.41, 39.13, 36.91, 32.49, 29.43, 26.65, 25.39.

HMRS-ESI (m/z): found $[M+H]^+$ 601.3003, C28H46ClN4O8 requires 601.30042.

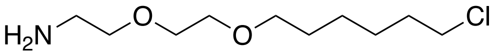
H2N
O
O
Cl

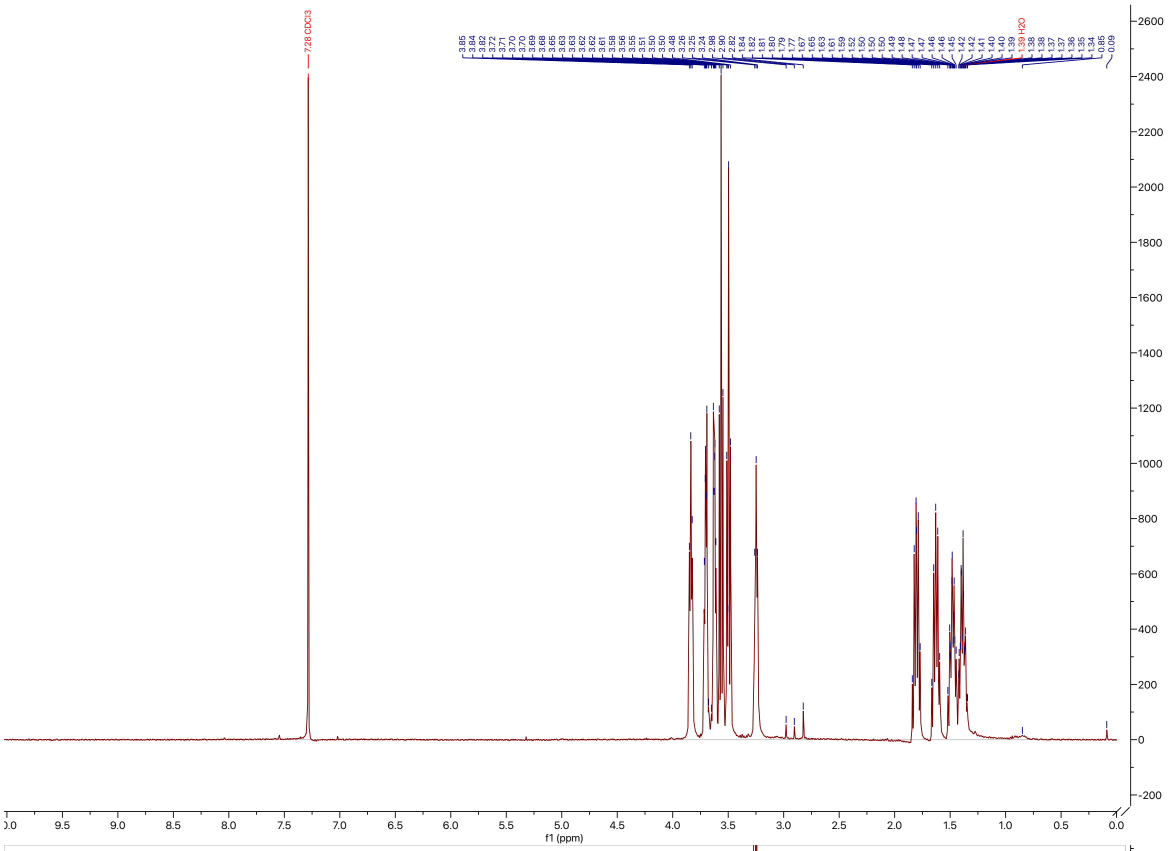
7.28 CDCl3
1.39 H2O
f1 (ppm)

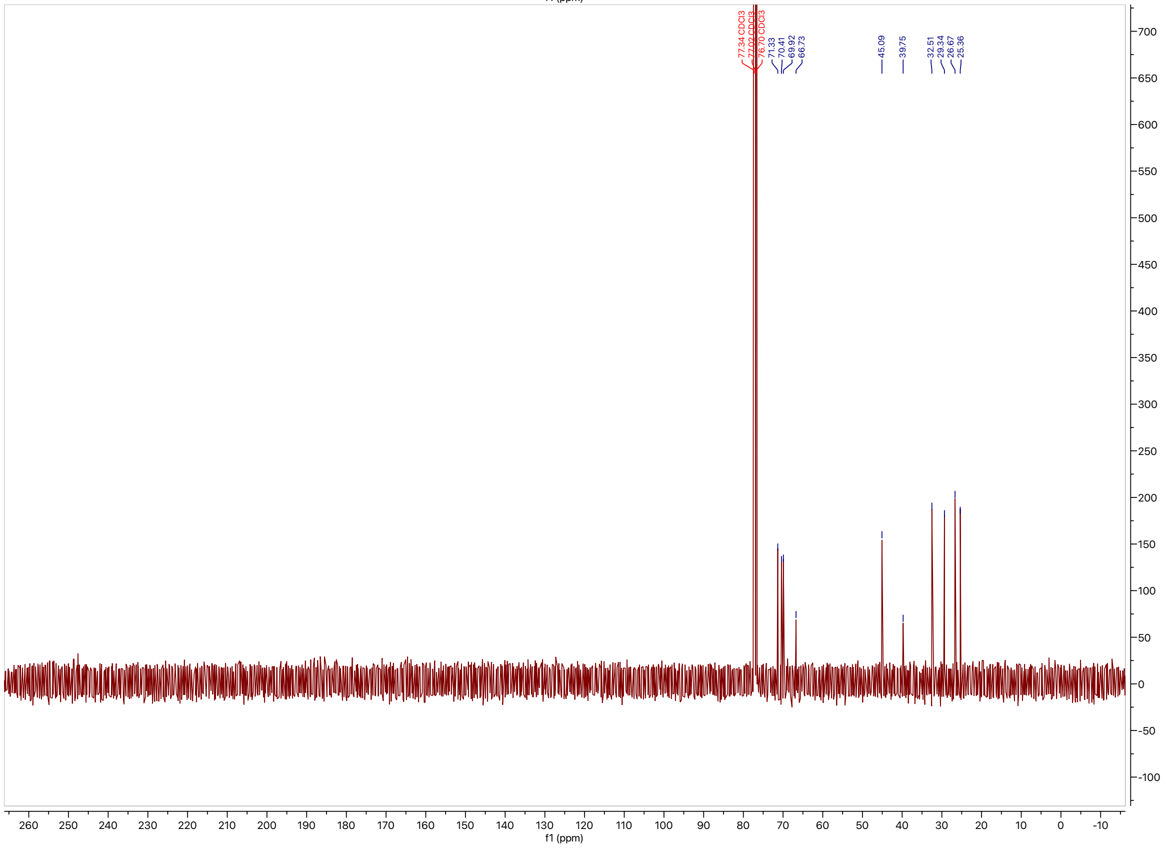
77.34 CDCl3
77.02 CDCl3
76.70 CDCl3
71.33
70.41
69.92
66.73
45.09
39.75
32.51
29.34
26.67
25.36
f1 (ppm)

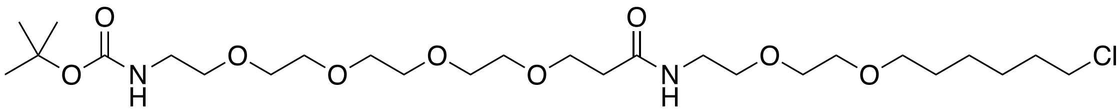

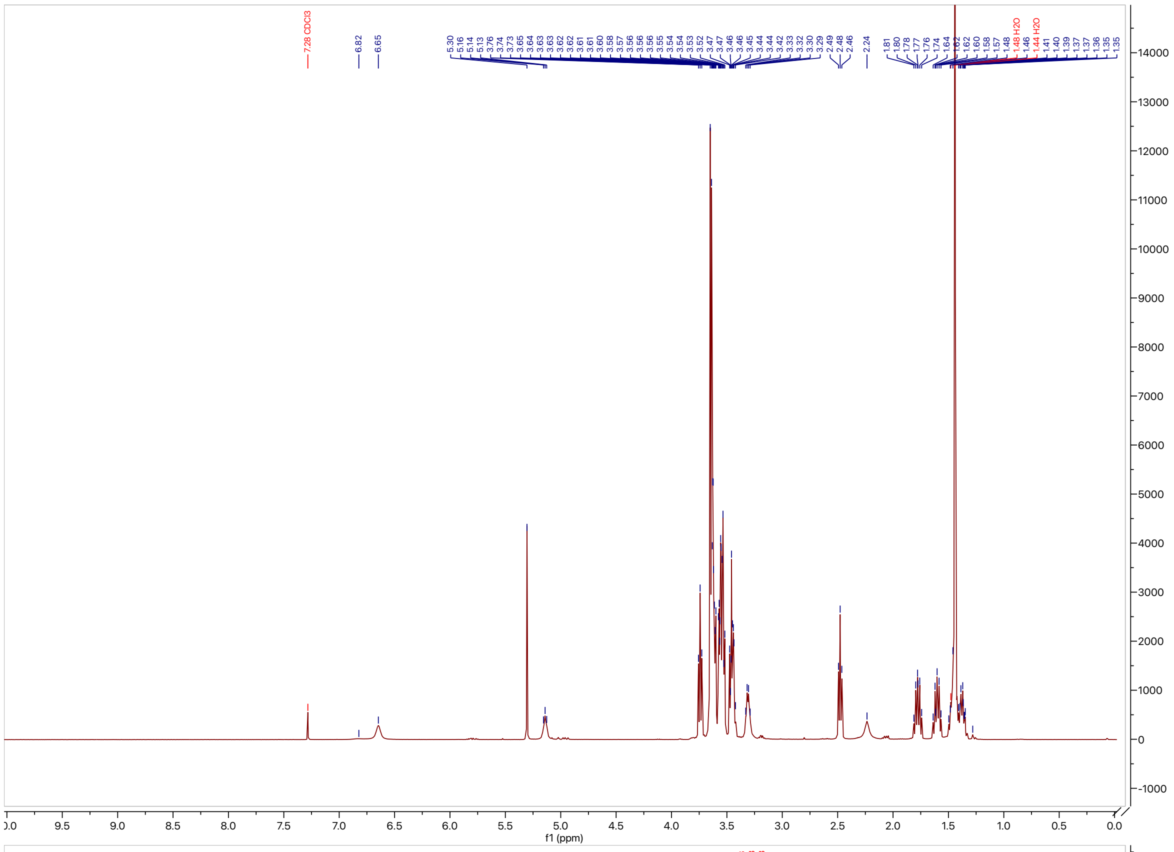

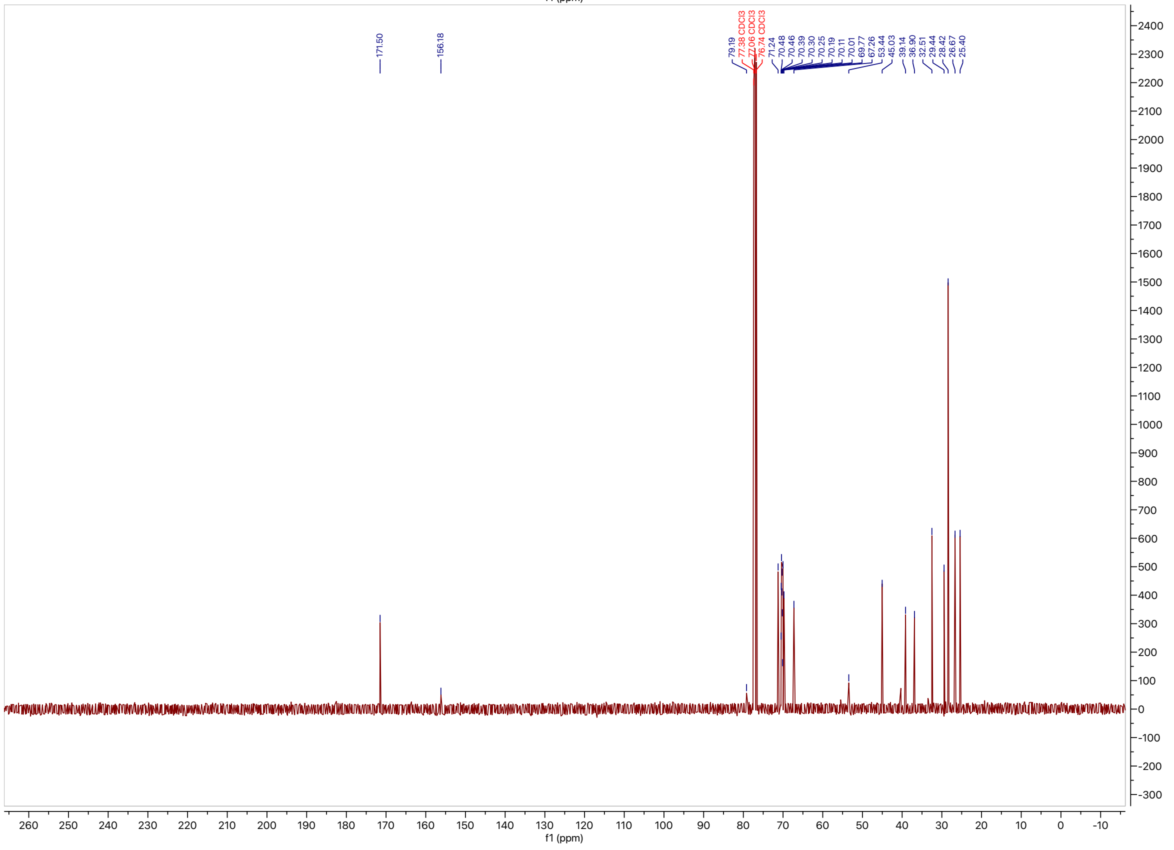

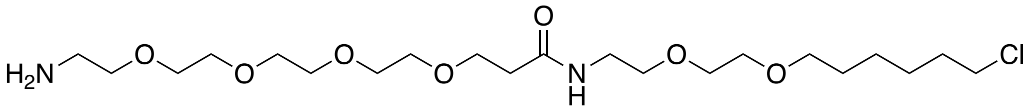
H2N
O
N
H
Cl

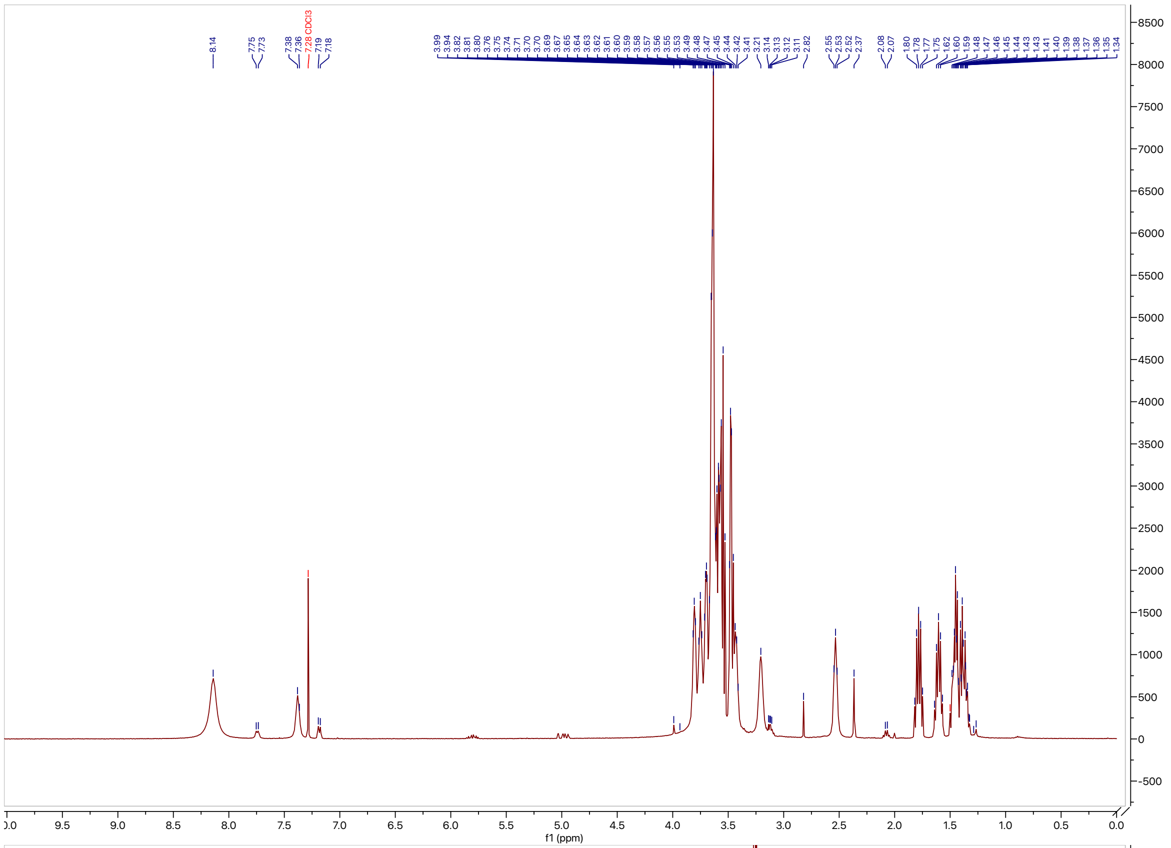
7.28 CDCl3
f1 (ppm)

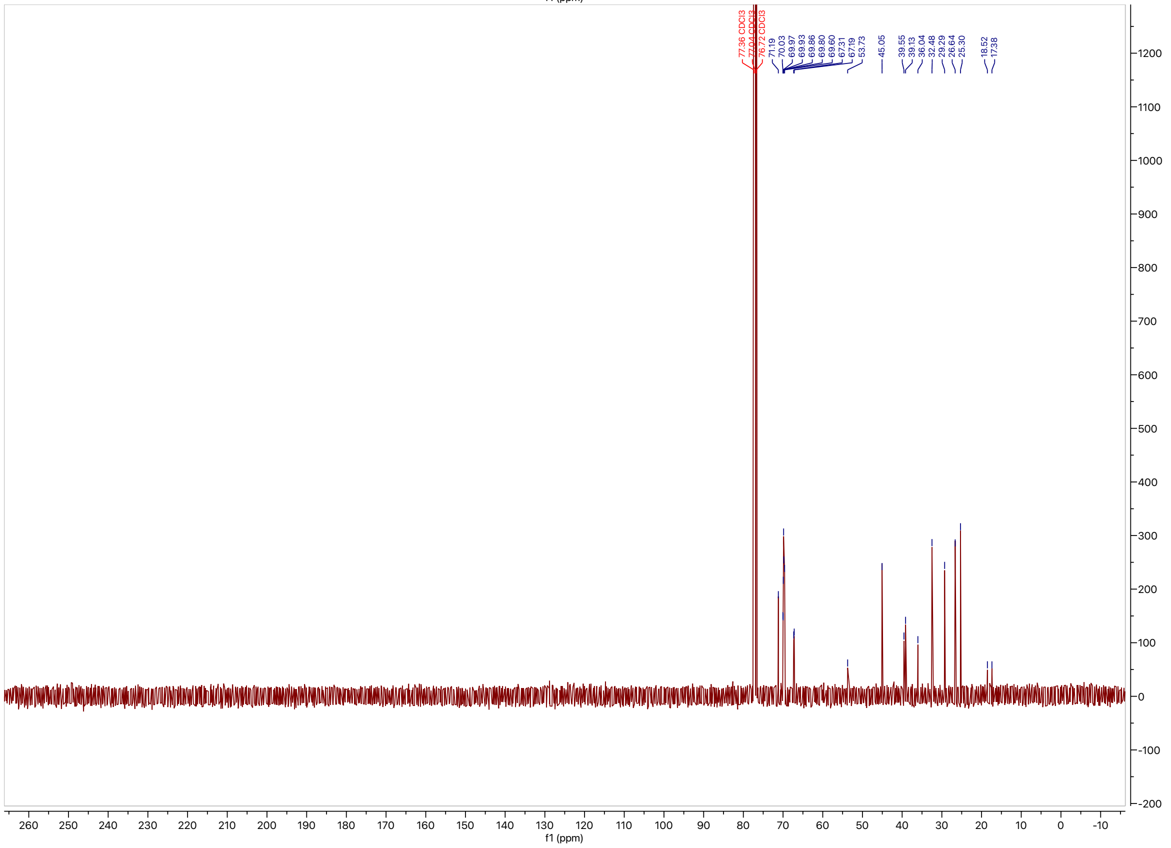
77.36 CDCl3
77.04 CDCl3
76.72 CDCl3
f1 (ppm)

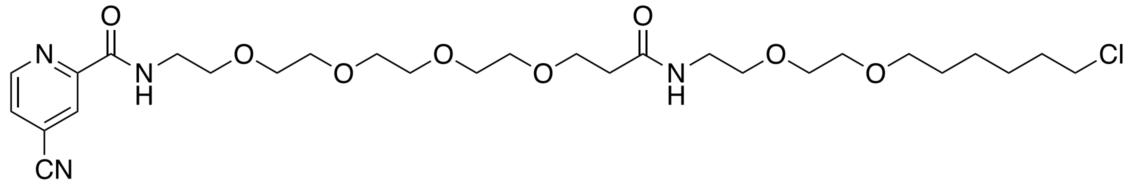

N
O
N
H
O
O
O
O
O
N
H
O
O
Cl
CN

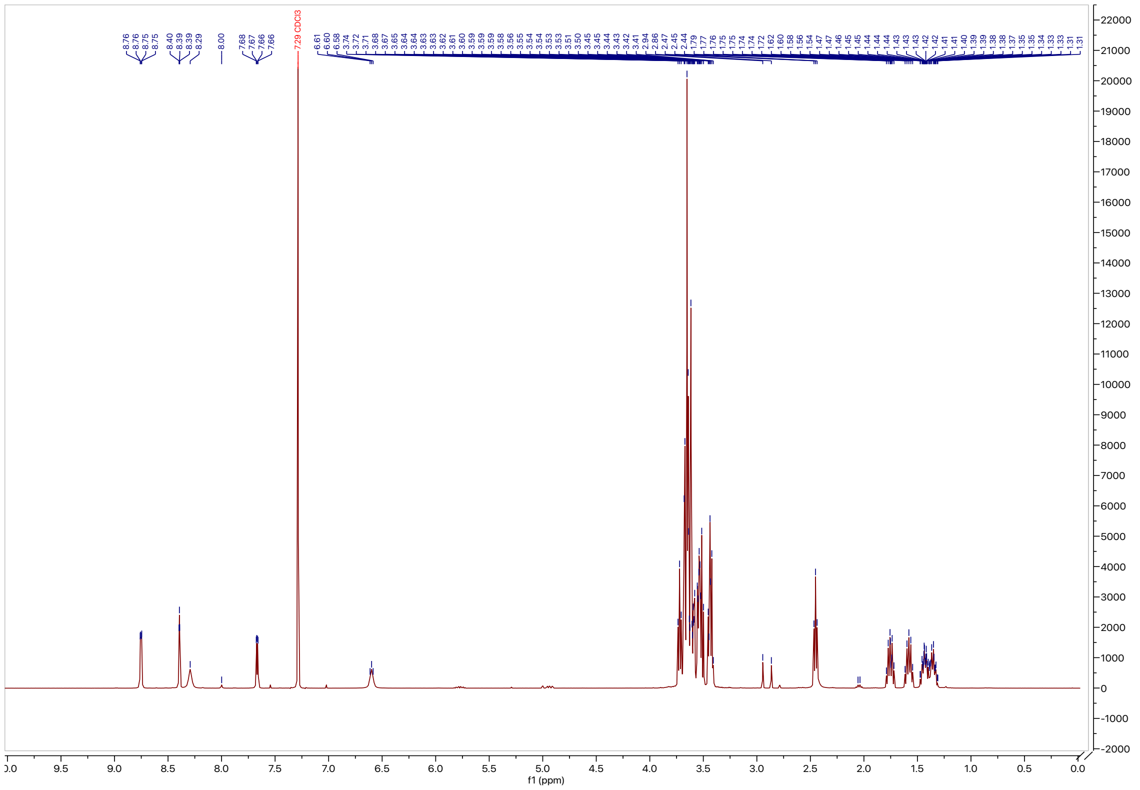

7.29 CDCl3
f1 (ppm)

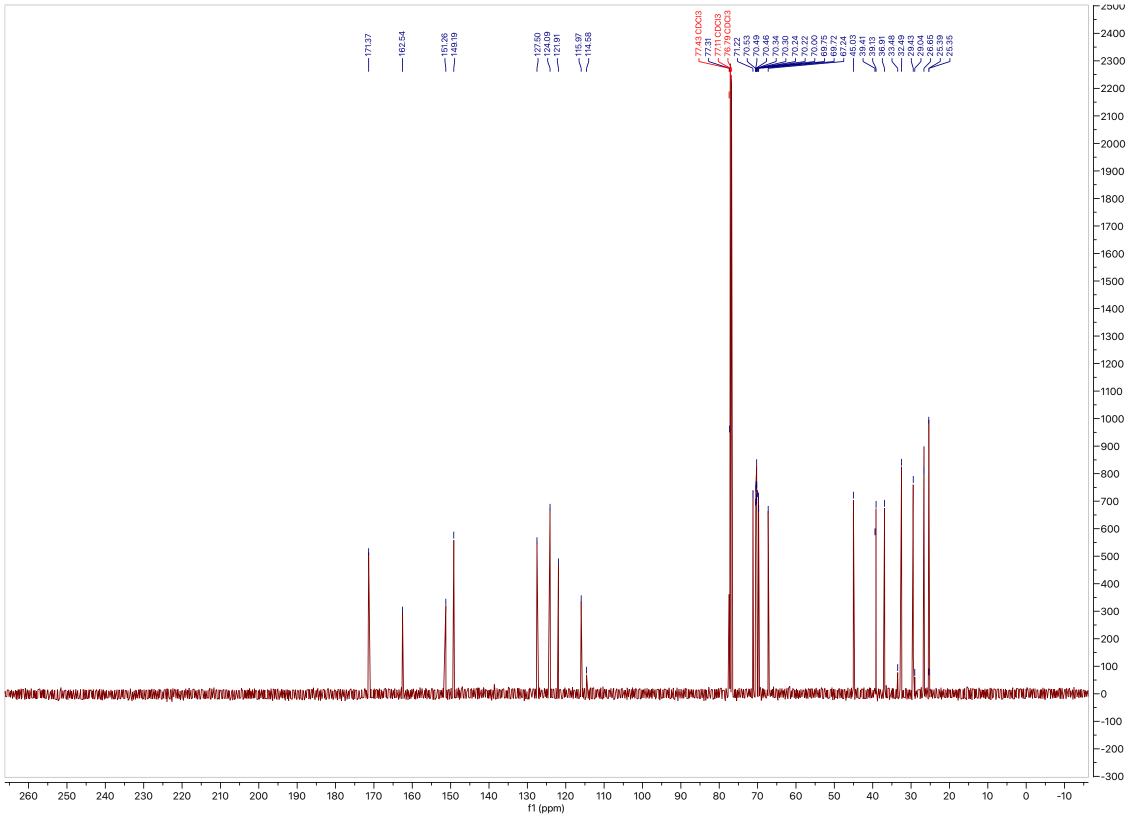

171.37
162.54
151.26
149.19
127.50
124.09
121.91
115.97
114.58
77.43 CDCl3
77.11 CDCl3
76.79 CDCl3
45.03
f1 (ppm)

Section 4.3. Enrichment Studies of 67nt m5C RNA

Buffer Solutions

**Buffer**	**Composition**
10x HaloTag Buffer	500mM HEPES (pH 7.5), 1500mM NaCl
Neutralisation Buffer	1M Tris (pH 12.0)
20x Proteinase K Buffer	1M Tris (pH 7.5), 100 mM $CaCl_2$

Nucleoside Selectivity of m5C vs m6A

HaloLig-CP LCMS spectra were recorded on an Acquity QDa (Waters) connected to an Acquity eLambda PDA detector (Waters). Nucleosides were analysed using an isocratic gradient of 80% MeCN vs. an aqueous solution of pH 3.7 10 mM NH_4OAc on a Merck SeQuant ZIC-HILIC column (100 Å, 3.5 µm, 150 x 4.6 mm).

Table S11: LCMS analysis of nucleoside pyridination reactions using HaloLig-CP and xanthone

Nucleoside	Time/h	Average dN/dI	% Remaining	Average Pyr/dI	% Pyr
m5C	0	7.482	-	-	-
m6A	0	19.769	-	-	-
m5C	20	3.083	41.21	2.002	26.77
m6A	20	14.036	71.00	0.000	0.00

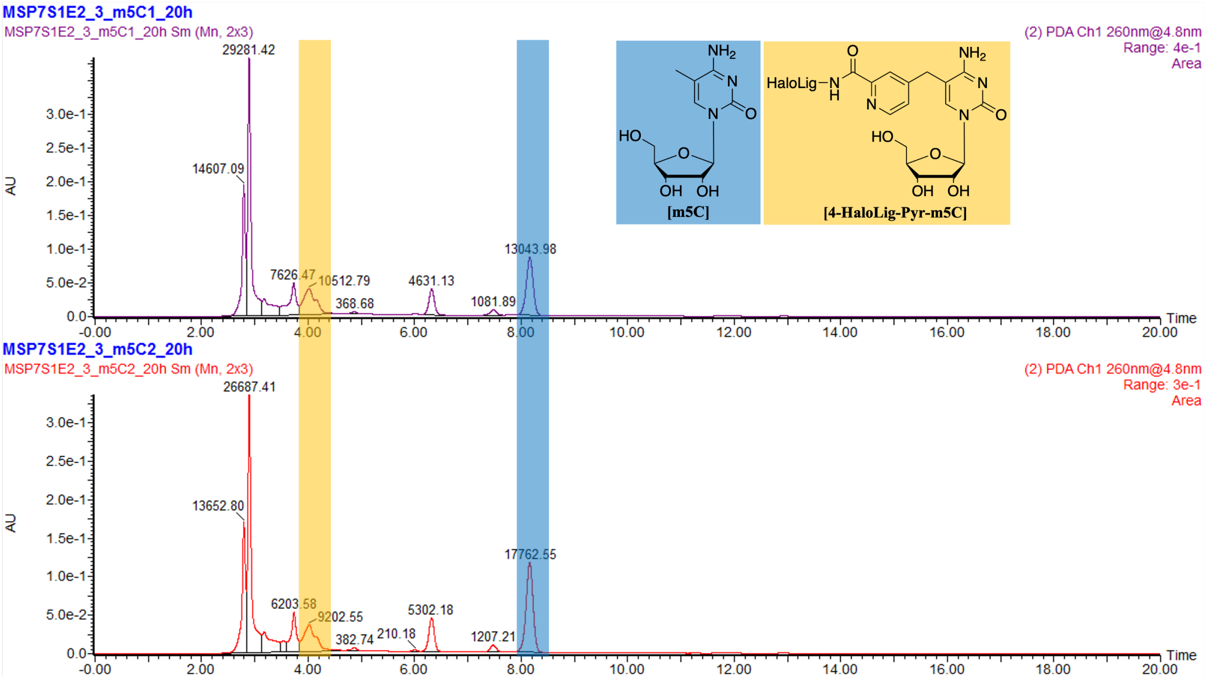

Figure S30: LCMS traces of the m5C reaction mixtures after pyridination reaction conditions following general procedure A with HaloLig-CP. From left to right: 4-HaloLig-Pyr-m5C (4.1 min), dI (6.3 min), m5C (8.2 min).

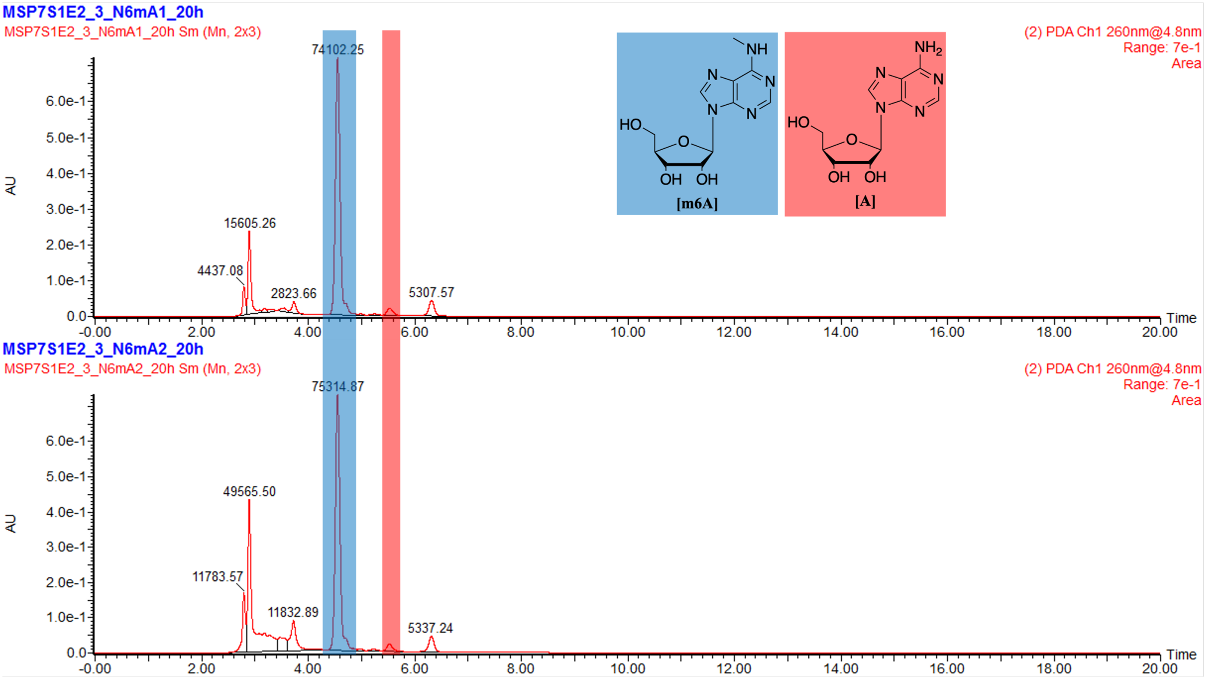

Figure S31: LCMS traces of the m6A reaction mixtures after pyridination reaction conditions following general procedure A with HaloLig-CP. From left to right: m6A (4.5 min), A (5.6 min), dI (6.3 min).

HaloTag Conjugation with a Short DNA Oligonucleotide

5mC 12mer was functionalised with a chloroalkane using HaloLig-CP according to General Procedure C (Figure S32). The products from multiple reactions were combined and purified by reverse-phase HPLC to isolate the chloroalkane-functionalised products, which will be referred to as HaloLig-12mer.

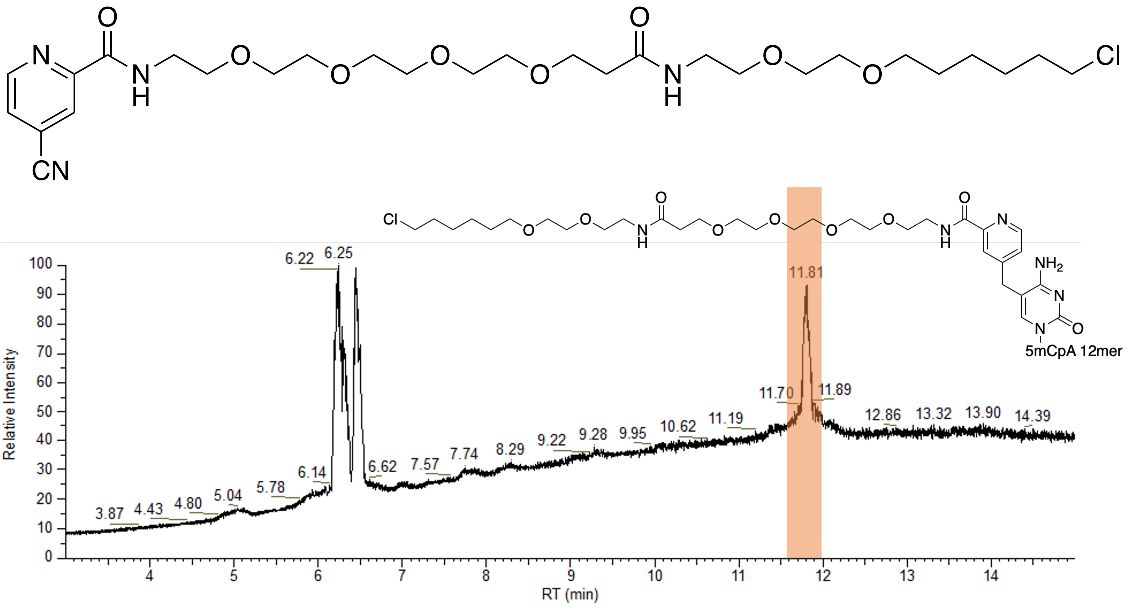

Figure S32: (Top) HaloLig-CP. (Bottom) High-resolution mass spectra (HRMS) of HaloLig-CP pyridination reaction products.

A side product was consistently observed which corresponded to a decrease in original oligonucleotide mass of 2 Daltons. This decrease in mass may be the result of intrastrand tandem lesions. The 5mC site in the 5mCpA 12mer test oligonucleotide is adjacent to two adenine residues. Generation of excess 5mC radicals under xanthone irradiation could lead to the formation of lesions such as Ade[6N,5-Me]5mC (Figure S33)[3]. The formation of these -2 Dalton adducts were most common on 5mCpA 12mer and increased when the concentration of cyanopyridine in the reaction was reduced. The -2 Dalton adduct was not observed on the m5C 10mer oligonucleotide. Furthermore, when oxygen was included in oligonucleotide reactions, the -2 Dalton adduct was not formed.

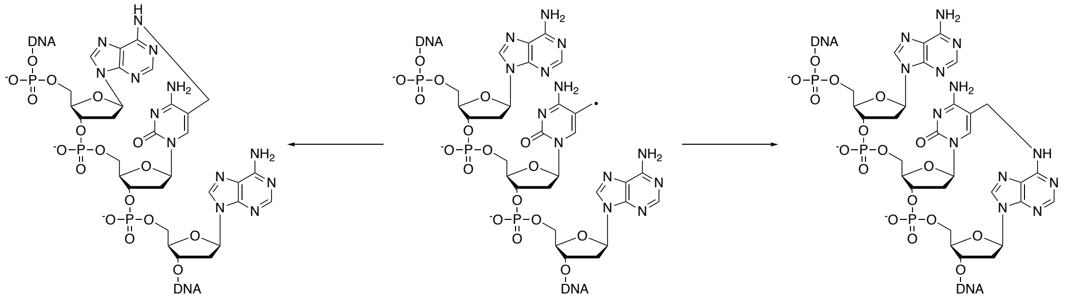

Figure S33: Plausible formation of intrastrand tandem lesions due to 5mC methyl radical formation withing 5mCpA 12mer.

HaloTag Conjugation of HaloLig-12mer:

• HaloLig-12mer	14 µL
• HaloTag-GST	4 µL
• 10x HaloTag Buffer	2 µL

The reaction was incubated on a shaker for 20 hours at 20 °C and 600 rpm. The reaction was then diluted to 70 µL with milliQ and purified using Zeba™ Spin Desalting Columns, 40K MWCO, 0.5 mL. The resulting sample was analysed by protein HRMS (Figure S34).

Expected product masses:
Unreacted HaloTag-GST + oligo mass – Cl =
C-CN substitution: 60512 + 4180 – 35 = 64657
Minisci C-H substitution: 60512 + 4205 – 35 = 64682

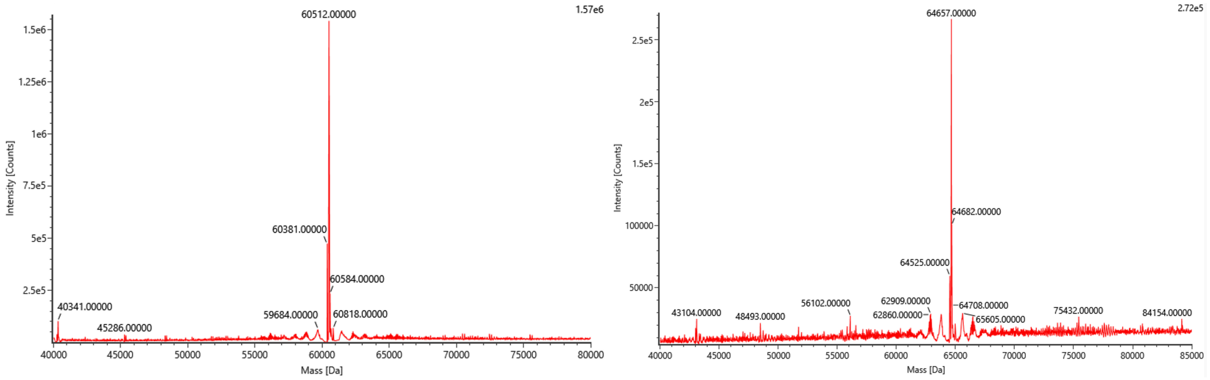

Figure S34: Protein HRMS of HaloTag-GST conjugation with HaloLig-12mer oligonucleotide showing unreacted HaloTag-GST (left) and conjugated HaloTag-GST (right).

Shorter chloroalkane-functionalised cyanopyridines were also tested and found to be suitable substrates for the pyridination reaction (Figure S35). However, oligonucleotides functionalised

with these shorter cyanopyridines were found to be poor substrates for HaloTag. A possible reason for the poor reactivity is that the oligonucleotide adduct may interfere with HaloTag binding. The surface of the HaloTag protein is negatively charged therefore binding to an oligonucleotide substrate could be significantly influenced by electrostatic interactions[4].

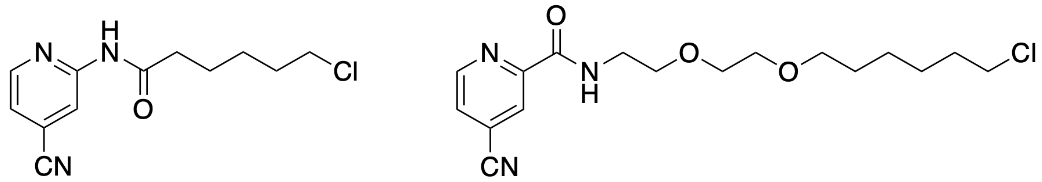

Figure S35: Structure of other cyanopyridines tested for HaloTag reactivity with shorter chloroalkanes.

Section 4.4. Halo-Trap Enrichment of m5C-Oligoribonucleotides

1. HaloTag Conjugation to RNA

m5C-containing RNA was functionalised using HaloLig-CP according to General Procedure D.

- Chloroalkane-functionalised RNA 17 µL
- HaloTag-GST 1 µL
- 10x HaloTag Buffer 2 µL

The reaction was incubated on a shaker for 20 hours at 20 °C and 600 rpm. Two HaloTag conjugation reactions were combined to a total volume of 40 µL.

2. HaloTrap Reaction:

Procedure and components are adapted from ChromoTek Halo-Trap Magnetic Agarose Kit.

- To 40 µL HaloTag reaction, add 160 µL Lysis buffer and 200 µL Dilution buffer (final volume 400 µL)
- Transfer 20 µL bead slurry to 1.5 mL tube (max binding capacity = 10 µg HaloTag)
- Add 400 µL ice-cold dilution buffer, separate until the supernatant is clear and remove the supernatant
- Add 400 µL sample to beads
- Rotate end-over-end for 3 hours at 4 °C
- Separate beads, discard supernatant, and resuspend in 400 µL wash buffer A
- Separate beads, discard supernatant, and resuspend in 400 µL wash buffer A
- Separate beads, discard supernatant, and resuspend in 400 µL wash buffer A
- Transfer beads to a new tube
- Separate beads and discard the supernatant
- Add 41 µL acidic elution buffer to beads and pipette up and down constantly for 60 seconds at room temperature
- Separate beads until supernatant is clear and transfer supernatant to a new tube
- Neutralize supernatant with 4 µL Neutralisation Buffer
- Add 41 µL Acidic elution buffer to beads and pipette up and down constantly for 60 seconds at room temperature
- Separate beads until supernatant is clear and transfer supernatant to a new tube

- Neutralize supernatant with 4 µL Neutralisation Buffer
- Final volume of neutralised supernatant 90 µL

3. Proteinase K

- Neutralised sample 90 µL
- 20x Proteinase K Buffer 5 µL
- Proteinase K (Thermo) 5 µL

The reaction was incubated at 30 °C for 30 minutes then the product was purified using RNA Clean and Concentrator-5 (Zymo) and eluted in 12 µL nuclease-free water.

4. cDNA Synthesis and qPCR

cDNA synthesis was performed using Thermo Scientific Maxima H Minus First Strand cDNA Synthesis Kit according to the manufacturer's instructions.

qPCR was carried out using Thermo Scientific Luminaris Color HiGreen qPCR Master Mix.

Section 5. Computational Studies

Section 5.1: Computational methodologies

Density functional theory (DFT) calculations were performed with Gaussian 16 (Revision B.01).[5] Structural optimizations and frequency calculations were conducted with the B3LYP-D3 functional and the 6-31G(d) basis set.[6–9] Optimized structures were verified through frequency analyses. All the geometries were confirmed to correspond to a minimum or a first-order saddle point on the potential surface. Singlet-point energy values of optimized structures were calculated at the ωB97X-D/6-311++G(d,p) level of theory.[10] 3D images of the optimized structures were generated with CYLview20.[11] Data analyses are conducted using Python packages, which includes NumPy (1.19.2), Pandas (1.1.3) and Plotly,[12] via Jupyter notebook. GoodVibes[13] python scripts were used in the data processing.

Conformational searching was required to provide initial estimations on dimer conformations and geometries of rotationally flexible molecules. Conformational searches were conducted in MacroModel (v11.7) with Maestro (release 2019-01)[14] using the Merck Molecular Force Field (MMFF)[15] with the mixed torsional / low-mode sampling method. A setting of 1000 as the maximum number of steps and a maximum number of steps per rotatable bond was used. Conformers within an energy window of 5.02 kcal mol^{-1} (21 kJ mol^{-1}) were saved and reoptimized with DFT calculations.

In this project, up to 100 conformers may be obtained from a conformational searching calculation. Reoptimizing all the conformers would be very computationally expensive and time-consuming. Hence, if a conformational searching output file contained more than 30 conformers, a Python script was used to select structures for reoptimisations (GitHub: https://github.com/Goodman-lab/CSearch_alignmol). The Python script's input was the conformational searching output files in xyz and sdf format, which contained coordinates of conformers in order of their relative energy based on the MMFF force field. The Python script selected every x-th structure and the structural outliers from the output file then returned the selected structure in gjf format. x was an integer. The value of x was chosen to keep the sample size of the selected conformers under 30 (i.e., x = 2-5 in the case of this project). The structural outliers were picked out with the AlignMol function from RDkit.[16] The AlignMol function compared the similarity between the two chemical structures and returned the root-mean-square deviation (RMSD) value. A large RMSD value implied that the two structures are distinctively different. The Python script produced an array of RMSD values for each conformer, which recorded the outcome of pairwise structural similarity comparisons of the conformer with other conformers in the output file. A conformer was identified as a 'structural outlier' when it met any one of the two criteria below:

1. None of the RMSD values in the array was less than 1.0.
2. An average value was computed for the RMSD values in the array. Conformers with an average RMSD value ranked within the top 5% were considered as the structural outliers.

For simplification, the deoxyribose ring of deoxyribose 5-methylcytosine (5mdC) is not included in the calculations as the ring is far away from the site of reaction. All 4-cyanopyridine derivatives in this computational investigation are 3-subsituted.

Section 5.2: The initial and refined mechanism

A. Initial mechanism

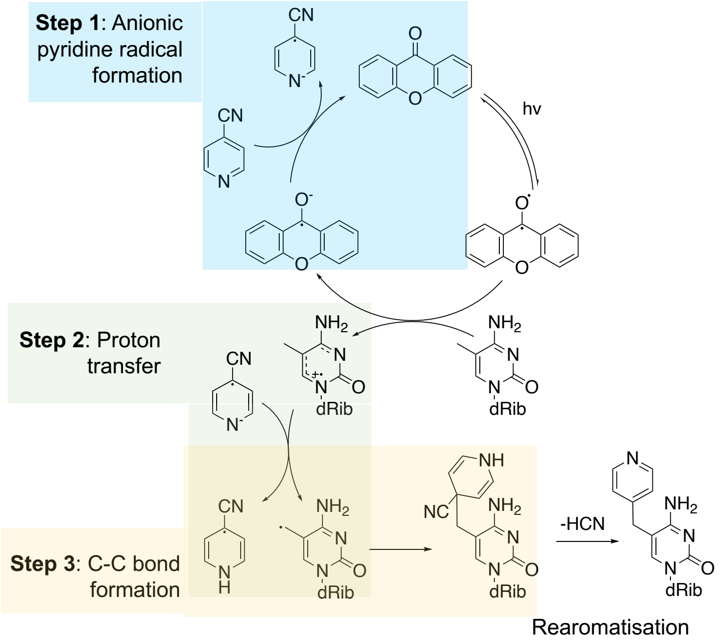

B. Refined mechanism

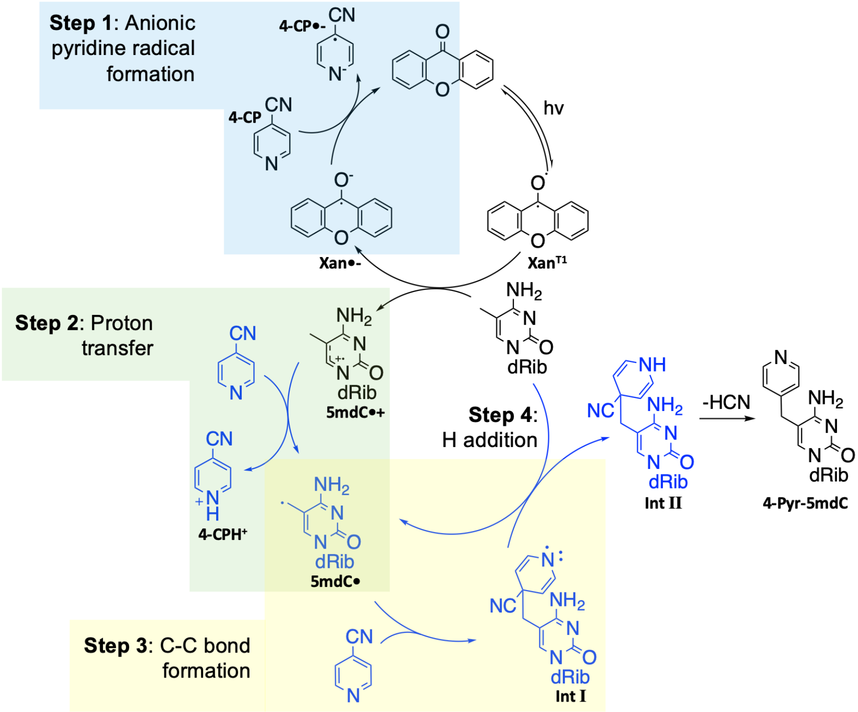

Figure S36: The initial and refined mechanism. A) The initial mechanism is based on previous literatures.[17–19] B) The refined mechanism upon computational studies. The changes from the initial proposal are coloured in blue. Here, the anionic cyanopyridine radical from step 1 is no longer essential for the sequential steps.

Section 5.3: Mechanistic revisions

Section 5.3.1: Step 2: Deprotonation of the cationic 5mdC radical

Various potential pathways have been considered for step 2 of the overall process. The neutral 5mdC radical may be generated via proton transfer from cationic 5mdC radicals to anionic cyanopyridine radicals, neutral cyanopyridine substrates or anionic xanthone radicals (Figure S37A, B and C). The solvent, water molecules, can also be involved in the proton transfer process (Figure S37D). Thorough thermodynamic and kinetic studies are conducted on these potential pathways.

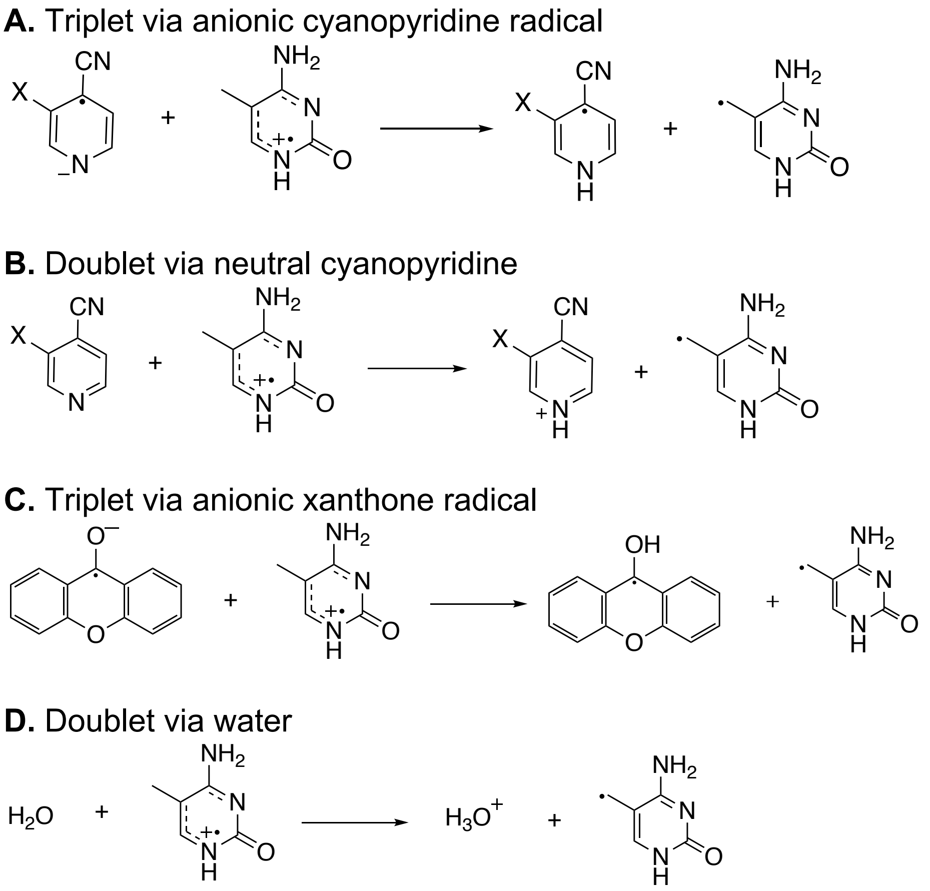

Figure S37: Possible pathways for the deprotonation of cationic 5mdC radical (step 2). A) the triplet pathway via anionic cyanopyridine radicals. B) the doublet pathway via neutral cyanopyridine substrates. C) the triplet pathway via anionic xanthone radicals. D) the doublet pathway via water, the solvent.

Thermodynamically, the triplet-based deprotonation pathways via anionic cyanopyridine derivatives (Figure S37A: ΔG ranges from -133.2 to -111.2 kcal mol^{-1}) and anionic xanthone radical (Figure S37C: ΔG = -80.4 kcal mol^{-1}) are spontaneous. These values are large because the starting material are charged, and they come together to form neutral products. The doublet pathway via neutral cyanopyridine is spontaneous for most cyanopyridine substrates (Figure S37B: ΔG = -7.5 to -0.7 kcal mol^{-1}), except for those with an electron-withdrawing group at position 3 (ΔG = 1.0 – 12.2 kcal mol^{-1}; Figure S45). The deprotonation of cationic 5mdC radicals via water is thermodynamically unfavourable (Figure S37D: ΔG = 44.3 kcal mol^{-1}).

In the kinetic studies, two different mechanisms have been considered. The cationic 5mdC radical deprotonation can occur directly at the methyl group (mechanism C) or at the amine group (mechanism A) followed by a sequential intramolecular H transfer. Kinetically, triplet-based proton transfer via an anionic xanthone radical ($\Delta G^‡(C)$ = 1.9 kcal mol^{-1}, $\Delta G^‡(A)$ = 6.9 kcal mol^{-1})[1] is the most favourable, followed by the doublet pathway via neutral cyanopyridine substrates ($\Delta G^‡(C)$ = 14.1 – 36.7 kcal mol^{-1}, $\Delta G^‡(A)$ = 5.6 – 49.3 kcal mol^{-1}; Figure S38) and the triplet pathway via anionic cyanopyridine radicals ($\Delta G^‡(C)$ = 12.9 – 28.5 kcal mol^{-1}, $\Delta G^‡(A)$ = 8.6 – 28.2 kcal mol^{-1}; Figure S39).

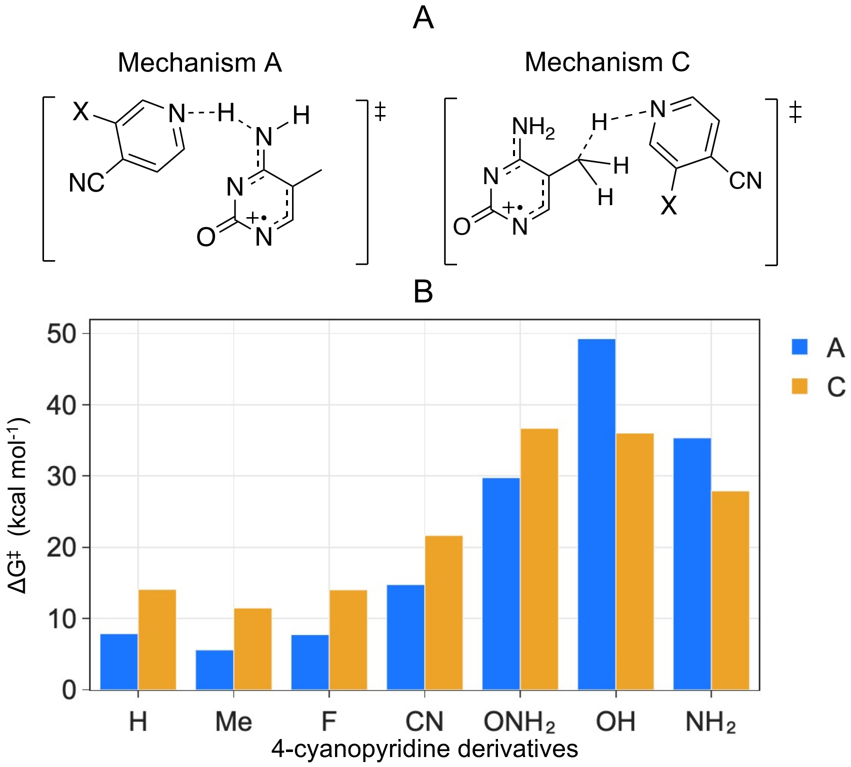

Figure S38: Kinetic studies of the doublet pathway via neutral cyanopyridine substrates. A) The two possible TSs for the proton transfer between neutral cyanopyridine substrates and cationic 5mdC radicals are given. B) The bar chart compares the $\Delta G^‡$ of the two possible TSs for each cyanopyridine derivative.

[1] The $\Delta G^‡$ in the text are from calculations at the ωB97X-D/6-311++G(d,p)//ωB97X-D/6-31G(d) level of theory. At the ωB97X-D/6-311++G(d,p)//B3LYP-D3/6-31G(d) level of theory, $\Delta G^‡$ = -1.13 kcal mol^{-1}.

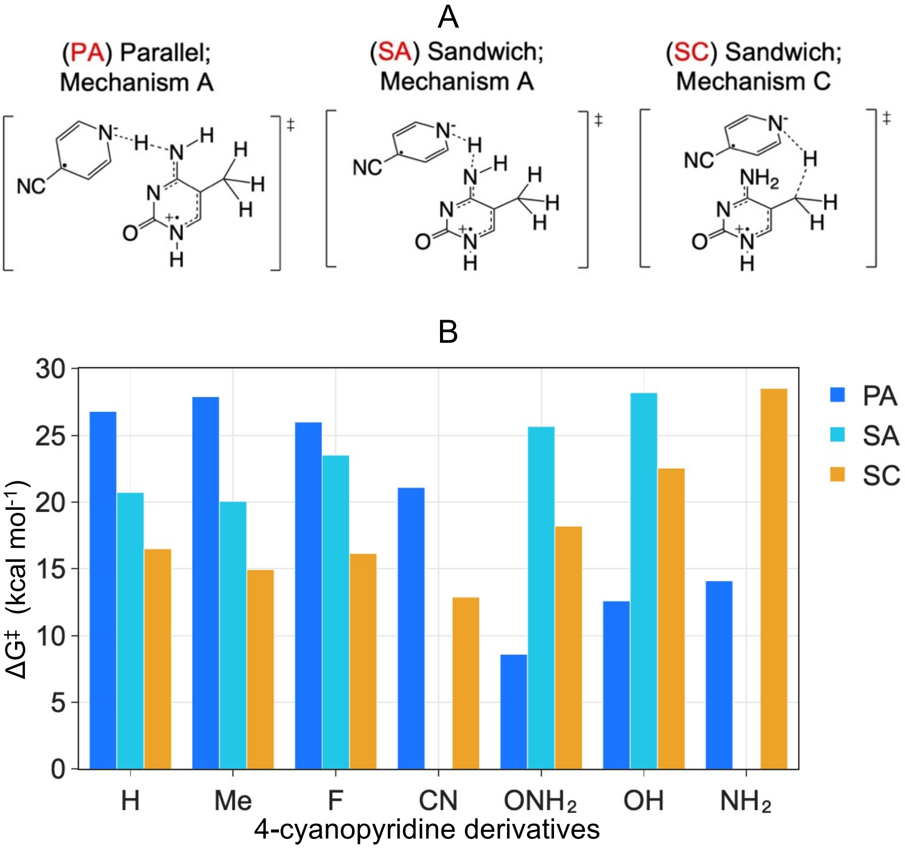

Figure S39: Kinetic studies of the triplet pathway via anionic cyanopyridine radical. A) The three possible TSs for the proton transfer between anionic 4-cyanopyridine radicals and cationic 5mdC radicals are given. The ring systems can take up either a sandwich or parallel conformation. B) The bar chart compares the $\Delta G^{\ddagger}$ of the three possible TSs for each cyanopyridine derivative. A few of the SA TSs have not been localized despite extensive searching. In these examples a conformation change from the sandwich conformation to the parallel conformation always occurred during the bond scan calculations.

With mechanism A, a sequential intramolecular H transfer is required to generate **5mdC•**. The H transfer can occur directly or with the assistance of a water molecule as a catalyst. Calculations show that the 5mdC intramolecular H transfer via water ($\Delta G^{\ddagger}$ = 18.4 kcal mol^{-1}) is the lower energy pathway compared to without water ($\Delta G^{\ddagger}$ = 25.7 kcal mol^{-1}) (Figure S40). For most of the bases, the $\Delta G^{\ddagger}$ value for the deprotonation processes at the amine group tend to be less than 18.4 kcal mol^{-1}. For these cases, the intramolecular H transfer step becomes rate-determining for step 2 of the overall mechanism if the deprotonation happens at the amine group.

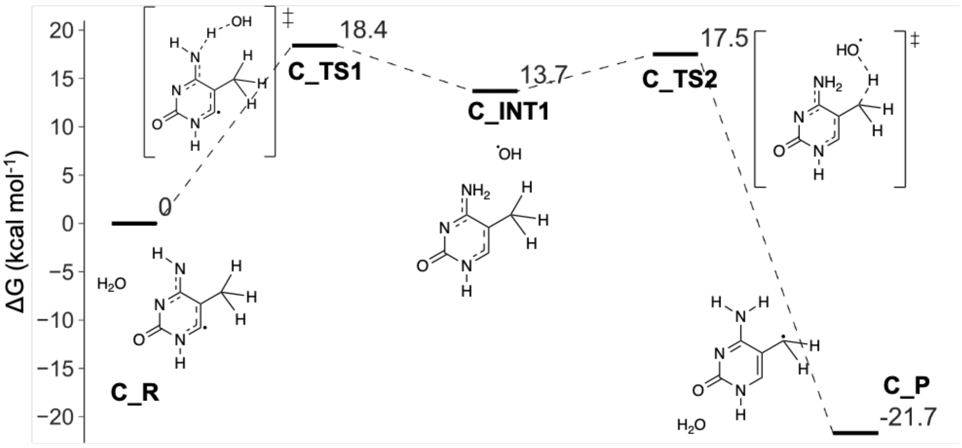

Figure S40: An energy profile for the intramolecular H transfer of 5mdC via water.

Based on the above calculations, the two triplet pathways and the doublet pathway with neutral cyanopyridine as the base (Figure S37A, B and C) are all theoretically possible in the reaction. However, triplet pathways involve the collision between two radical species. Due to the excess of cyanopyridine (300 mM) compared to xanthone (10 mM) used in the reaction, the concentration of anionic radical species in the reaction will be low compared to neutral cyanopyridine. The chance of a collision between two radicals in the triplet pathways is low. Therefore, **5mdC•+** is most likely to be deprotonated at the methyl position by neutral cyanopyridine to form **5mdC•**. The pathway via neutral cyanopyridine derivatives supports the experimental data and explains the variation in the percentage yield of the cyanopyridine substrates (see section 5.4.2 for more details). If the alternative triplet pathway via anionic xanthone radical dominates, cyanopyridine substrates are not required to accept net charges and changing the electronic nature of the cyanopyridine ring should not affect the percentage yield of the reaction to a large extent.

In the doublet pathway with cyanopyridine substrates, direct deprotonation at the methyl group (mechanism C) is more likely to occur than deprotonation at the amine (mechanism A). Mechanism A is less favourable kinetically compared to mechanism C due to the high kinetic barrier of the sequential intramolecular H transfer. Furthermore, deprotonation at the amine group is also slightly less thermodynamically favourable compared to deprotonation at the methyl group (e.g., for 4-cyanopyridine, $\Delta G(C) = -1.1$ and $\Delta G(A) = 14.6$ kcal mol^{-1}).

Section 5.3.2: Step 3: triplet vs doublet vs single pathway

Step 3 of the reaction involves a C-C bond formation between C4 in cyanopyridine and $-CH_2$ in a 5mdC radical (Figure S41). Radical-radical coupling mechanisms have been proposed for similar reactions in literature. The coupling can occur between a neutral cyanopyridine radical and a 5mdC radical via a neutral triplet TS.[17] As neutral cyanopyridine radicals can only be generated in the minor triplet pathway of step 2, the kinetics of other possible pathways were examined. An anion triplet pathway is also plausible, where the coupling happens between an anionic cyanopyridine radical and a neutral 5mdC radical.[20–22] Besides radical-radical coupling terminations, the C-C bond formation can also take place via a doublet or a singlet TS. In the doublet pathway, the C-C bond is formed between a neutral cyanopyridine and a 5mdC radical. In the singlet pathway, the radicals convert into ions and the C-C bond formation occurs between ions.

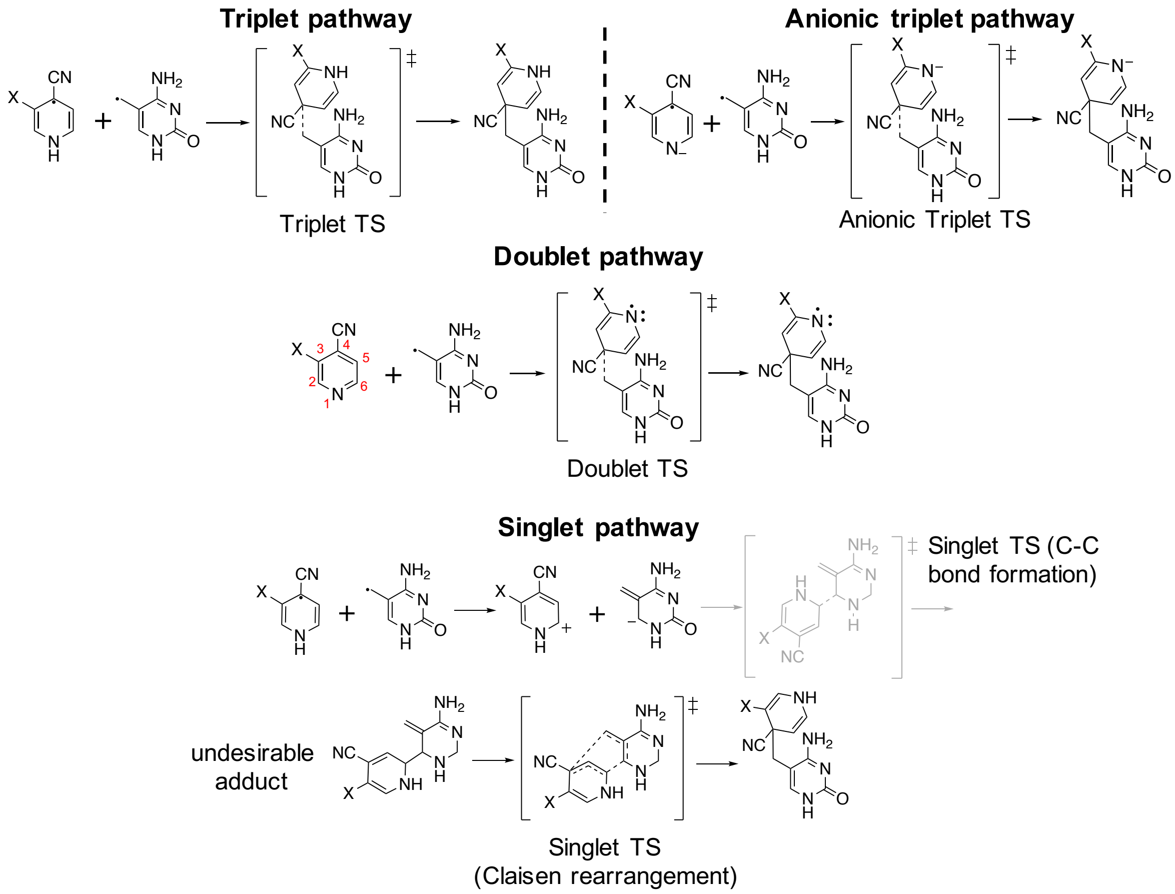

Figure S41: The four possible C-C bond formation pathways.

Transition state analyses were conducted on the four possible pathways. Neutral triplet TSs, anionic triplet TSs and doublet TSs were found. $\Delta G^{\ddagger}$ values of these TSs were calculated (Figure S42). Bond scan calculations of the adduct with a singlet state setting reveals that the singlet pathway is a two-step process. The ion-ion coupling reaction leads to an undesirable adduct, which can undergo a Claisen rearrangement reaction to give the desirable adduct. The TS of the ion-ion coupling reaction were not obtained, presumably due to the barrierless nature of the process. The Claisen rearrangement reaction TSs were found and optimised (Figure S42). $\Delta G^{\ddagger}$ values of neutral triplet TSs are significantly higher compared to the other pathways. Therefore, a radical-radical coupling mechanism via a neutral triplet TS is not the favourable

pathway for the C-C bond formation. The difference in ΔG‡ between the three other possible TSs are often less than 2 kcal mol^{-1}. In theory, the three possible pathways are all likely to occur. As explained in section 5.3.1, the concentration of radical species is likely to be low relative to the concentration of neutral cyanopyridine. Therefore, C-C bond formation is most likely to occur via the doublet pathway.

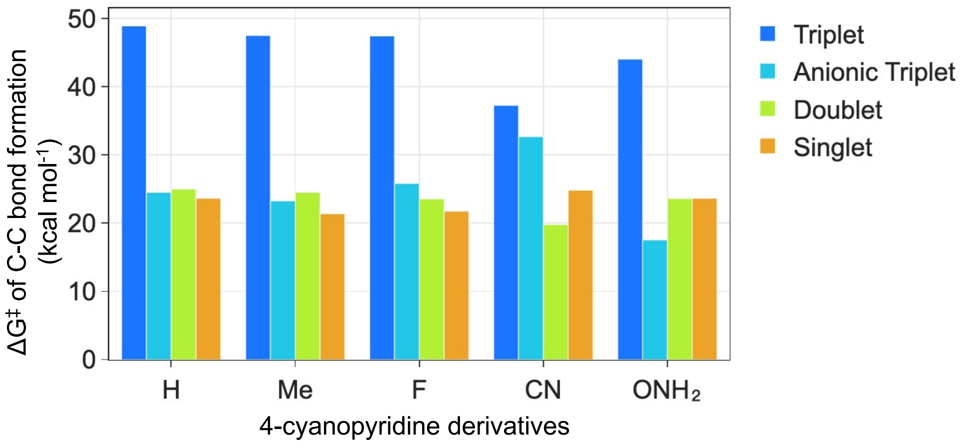

Figure S42: The kinetic study of the four possible C-C bond formation pathways: The bar chart compares the ΔG‡ of the TSs from the four possible pathways for each cyanopyridine derivative.

Section 5.3.3: Step 4: H addition

After establishing the mechanism for the C-C bond formation between cyanopyridine and 5mdC, we deduced that the sequential step should be H addition of the doublet adduct. H addition can occur either via a neutral 5mdC molecule or via water or acetonitrile, the solvent of the reaction.

A simplified system was used for investigating the H addition process (Scheme S3). The H transfer from 5mdC to cyanopyridine can occur with a parallel or a sandwich conformation via direct H transfer from the methyl group (Mechanism C) or H transfer from the amine group followed by a 5mdC intramolecular H transfer (Mechanism A). There are four possible TS conformations: SA (sandwich, mechanism A), PA (parallel, mechanism A), SC (sandwich, mechanism C) and PC (parallel, mechanism C).

Scheme S3: A simplified model of the H addition reaction via neutral 5mdC

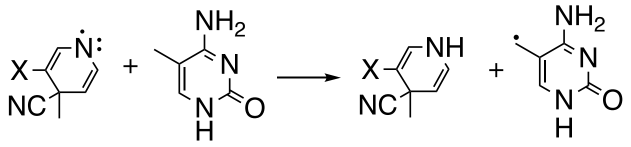

Thorough kinetic studies were conducted on pathways via 5mdC and water (Figure S43). TSs of H addition via water have a considerably higher ΔG‡ value than TSs of H addition via 5mdC. For 4-CP, H addition via another 5mdC molecule (ΔG = 2.6, ΔG‡ = 25.2) is both thermodynamically and kinetically more favourable than H addition via either water (ΔG = 30.2, ΔG‡ = 34.7) or MeCN (ΔG = 7.3, ΔG‡ = 29.9).

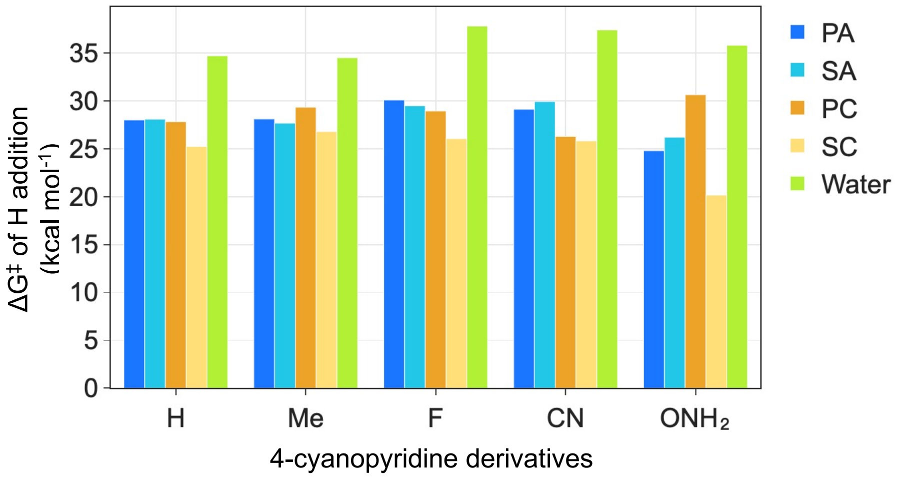

Figure S43: Kinetic studies of the H addition process: the bar chart compares $\Delta G^{\ddagger}$ values of possible H addition processes via water and via 5mdC

Among the possible TS conformations for pathways via 5mdC, TSs with a SC conformation have the lowest $\Delta G^{\ddagger}$ value across a range of cyanopyridine substrates. The $\Delta G^{\ddagger}$ difference between the four possible TSs for pathways via 5mdC is relatively small, therefore mechanism A may still be competitive. Taking 3-methyl-4-cyanopyridine as an example, the four TSs have a $\Delta G^{\ddagger}$ difference within 2.5 kcal mol^{-1}.

Section 5.4: Variation in percentage yield

Section 5.4.1: Thermodynamic study

ΔG values of step 1 and 2 (ΔG_{step1} and ΔG_{step2}) were computed for a range of 4-cyanopyridine substrates. The correlation between the thermodynamic result and the electronic nature of the cyanopyridine rings was explored through comparisons with Hirshfeld charge results. Specifically, data of the sum of Hirshfeld charges on the substituent of neutral cyanopyridine substrates were gathered and analysed. Besides the calculated data sets, Hammett sigma constants from the literature were also introduced for validation.[23] Hammett sigma constants (σ_x) are experimental values based on the ionization process of benzoic acid:

$$\sigma_x = \log K_X - \log K_H \qquad (1)$$

K_H is the ionization constant for benzoic acid in water. K_X is the ionization constant for a meta-substituted benzoic acid. σ_x is a collective measure of the substituent's ability to withdraw or donate electrons from the site of reaction. The Hammett sigma constant of an electron-withdrawing (EW) substituent, such as -CN, tends to be positive and vice versa for the Hammett sigma constant of an electron-donating (ED) substituent.

ΔG_{step1} values are plotted against Hammett sigma constants (Figure S44A). A noticeable change in trend is observed at approximately $\sigma_x = 0.2$. When $\sigma_x > 0.2$, ΔG_{step1} values and Hammett sigma constants follow a negative linear trend. Electron-withdrawing groups withdraw charges from the ring system and make the ring more positive, favouring the electron transfer process from xanthone to neutral cyanopyridine substrates. Hence, substrates with a strong electron-withdrawing substituent have a more negative ΔG_{step1} than substrates with a weak electron-withdrawing or an electron-donating substituent. When $\sigma_x < 0.2$, the ΔG_{step1} values stay the same regardless of the electron-donating strength of the substituent.

Charge analyses provide insights into the electronic nature of the cyanopyridine rings. ΔG_{step1} values are plotted against the sum of Hirshfeld charges on the substituent of neutral cyanopyridine substrates (Figure S44B). The positive linear trend (Pearson's r = 0.75) in the plot demonstrates that a more negative ΔG_{step1} value is found for substrates with a stronger electron-withdrawing substituent. Interestingly, regardless of the electron-donating strength, the charges located on the electron-donating substituents are very similar across different cyanopyridine substrates. Similarly, the variation of ΔG_{step1} values for substrates with an electron-donating group is insignificant.

For step 2, a positive linear correlation has been observed in the ΔG_{step2} vs Hammett sigma constants plot with no obvious change in trend (Figure S44C). The presence of an electron-withdrawing group leads to a relatively positive ring system, which makes the acceptance of a proton less favourable. On the other hand, having an electron-donating group makes the deprotonation process more favourable due to the donation of electron density from the substituent to the ring.

The negative linear trend on the plot of ΔG_{step2} values against the sum of Hirshfeld charges demonstrates that the thermodynamics of step 2 is also directly related to the electronic nature of the cyanopyridine rings (Pearson's r = -0.88) (Figure S44D). Substrates with a stronger electron-withdrawing group have less negative ΔG_{step2} and more negative charges located on the substituent. Substrates with an electron-donating group tend to have less negative charge located at their substituents and more electron density concentrated within the ring, making the process more thermodynamically favourable.

In summary, substrates with an electron-withdrawing group are more favourable in step 1 but not in step 2. An increase in the strength of the substituents' electron-withdrawing ability leads to a lower percentage yield. In step 2, substrates with an electron-donating group gives a spontaneous process. In step 1, regardless of the substituent's electron-donating strength, similar ΔG and charge analysis results are obtained. Hence, thermodynamics parameters alone are not enough to explain the variations in percentage yield across different substrates with an electron-donating group. Therefore, other factors, such as reaction kinetics, must be considered.

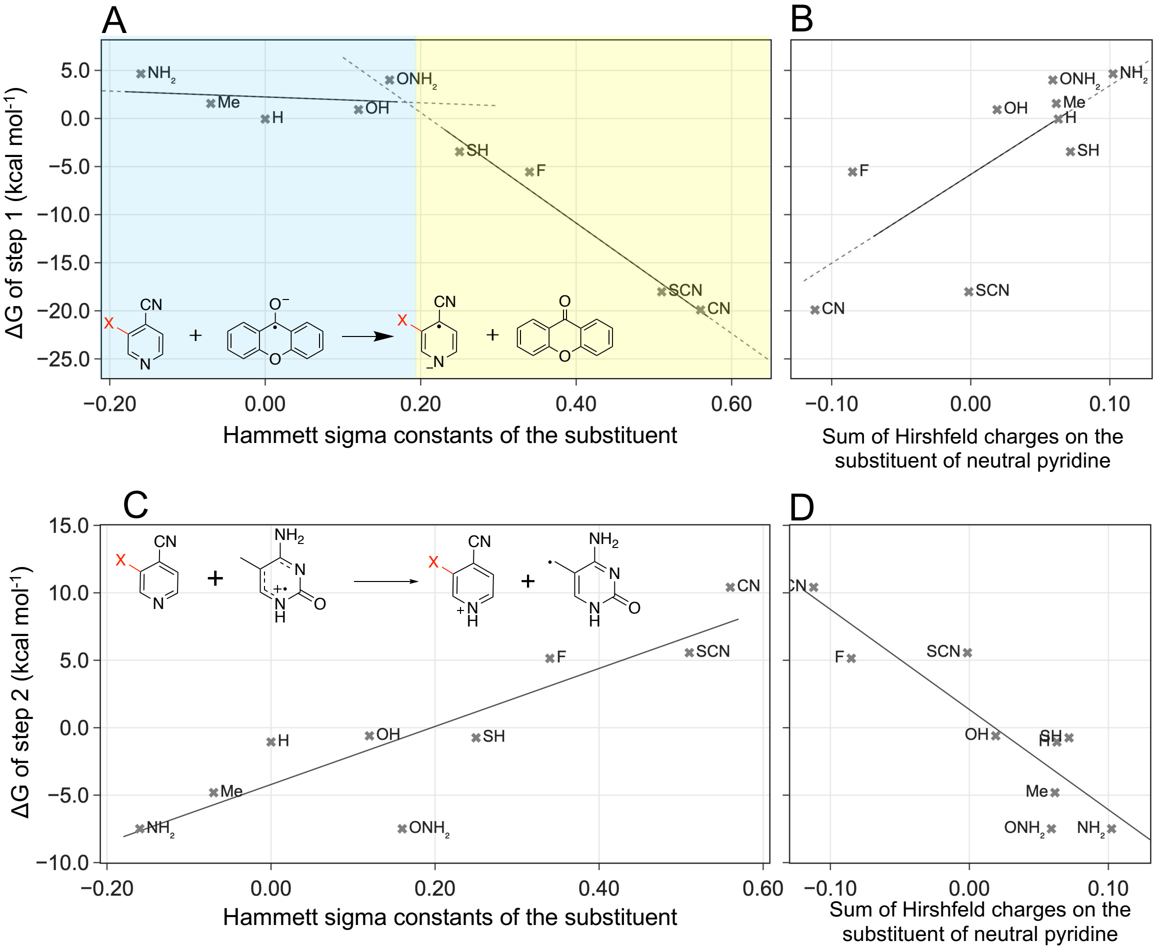

Figure S44: An overview of the thermodynamic and charge analysis results for step 1 and 2: A) ΔG_{step1} vs Hammett sigma constants of the substituent plot (Gradient of the best-fit lines: blue region: -3.0; yellow region: -57.4). The change in trend occurs at approximately $\sigma_x = 0.2$, which is indicated by the background colouring in plot A and C. B) ΔG_{step1} vs the sum of Hirshfeld charges on the substituent of the neutral cyanopyridine substrate plot. C) ΔG_{step2} vs Hammett sigma constants of the substituent plot (Gradient of the best-fit lines: 21.5). D) ΔG_{step2} vs the sum of Hirshfeld charges on the substituent of neutral cyanopyridine substrates plot. Each point on the plots represents a cyanopyridine derivative. The substituent labels are next to the data points. The chemical equations of step 1 and 2 for pyridination with 4-cyanopyridine are drawn and placed inside plot A and C, respectively.

Section 5.4.2: Kinetic study

A. Step 1: Anionic Cyanopyridine Formations

Step 1 of the reaction involves the formation of a dimer between cyanopyridine and anionic xanthone radical. Conformational searches and DFT optimisations have been conducted to explore the possible dimer conformations for cyanopyridine substrates. Regardless of the substituent, stable conformers always have a sandwich conformation due to favourable π–π stacking interactions.

Thorough examinations of the structure and relative energy compared to the most stable conformer (ΔG) provide insights into the potential surface of the anionic xanthone-pyridine radical dimer. For most of the cyanopyridine substrates (X = -H, -F, -CN), the dimer with xanthone has a flat potential surface. ΔG of the conformers varies by less than 0.5 kcal mol^{-1}, but the relative orientations between xanthone and cyanopyridine are different (Figure S45). The dimer potential surface for substrates with an electron-donating group (X = -Me, $-ONH_2$) has slightly different characteristics. For example, conformer 4ONH2_1_a and 4ONH2_1_b in Figure S37 both have a sandwich orientation, but the ΔG between the two geometries is 7.91 kcal mol^{-1}. Electron-donating groups also tend to be excellent hydrogen bond donors. H bonds between the electron-donating group and xanthone become one of the dominant factors that impact conformers' energy. Strong H bonding interactions between cyanopyridine and xanthone lead to a noticeable stabilisation in energy. This specificity in bonding within the dimer of xanthone and substrates with a good H bond donor is likely to contribute to barriers in dimer dissociation after the electron transfer.

The difficulties in dissociation are also reflected thermodynamically on the ΔG of dissociation (ΔG_d). Substrates with a -Me (ΔG_d = 7.7 kcal mol^{-1}) or $-ONH_2$ group (ΔG_d = 15.6 kcal mol^{-1}) have a noticeably more positive ΔG of dimer dissociation compared to other substrates, such as substrates with a -F (ΔG_d = 2.6 kcal mol^{-1}) or a -CN group (ΔG_d = 0.8 kcal mol^{-1}). Nevertheless, the disfavours in dimer dissociation are probably not the main factors that lead to the poor reactivity and the low percentage yield for these substrates. Firstly, the dissociation of the dimer might not be essential for the sequential step to take place. Secondly, the ΔG_d contributes to the overall ΔG of step 1. When the Hammett sigma constant is less than 0.2, the variation in ΔG_{step1} values is insignificant.

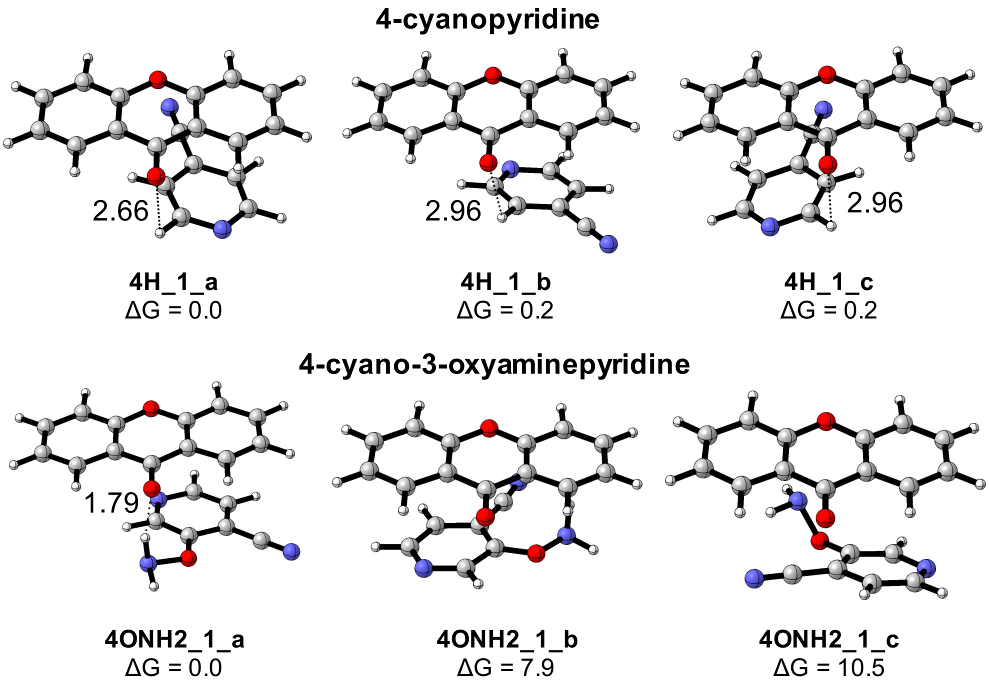

Figure S45: Conformers of anionic xanthone-pyridine radicals: The result for 4-cyanopyridine and 3-hydroxylamine-4-cyanopyridine are presented. The ΔG value refers to the relative energy compared to the most stable conformer in the series. (Unit: kcal mol^{-1})

B. Step 2: The Proton Transfer

The major pathway for the deprotonation of cationic 5mdC radical is via a double pathway with neutral cyanopyridine substrates. The preferred mechanism is mechanism C with deprotonation at the methyl group.

Scheme S4: Step 2 – deprotonation of cationic 5mdC radical

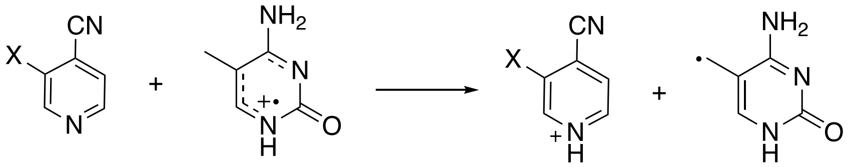

The $\Delta G^{\ddagger}$ values for deprotonation are much higher for cyanopyridine substrates with -OH, -NH_2 and -ONH_2 groups ($\Delta G^{\ddagger}$ = 27.9 – 36.7 kcal mol^{-1}) compared to other cyanopyridine substrates ($\Delta G^{\ddagger}$ = 11.0 – 22.9 kcal mol^{-1}) (Figure S46). Mechanism C TSs for the doublet pathway with different cyanopyridine substrates have highly similar structures (Figure S46A). Therefore, the high kinetic barrier is due to the initial reactant dimer conformation between neutral cyanopyridine and 5mdC•+. The most stable reactant dimer conformation with the -NH_2 substituted cyanopyridine is stabilized by H-bonding (Figure S46B). More stabilised reactant dimers have a greater energy difference between the reactant dimer and the TS.

We want to highlight an outlier of the trend, 3-hydroxy-4-cyanopyridine. Compared to other cyanopyridine substrates with an electron-donating group, the reaction with the -OH substituted substrate gives a slightly higher percentage yield. However, the results of the calculations, including thermodynamic and kinetic data, are not particularly different compared to other substrates with an electron-donating group. However, we have unintendedly obtained

strange TS structures during the investigation for 3-hydroxy-4-cyanopyridine (Figure S47). This hints that 3-hydroxy-4-cyanopyridine may adopt a slightly different pathway than other substrates.

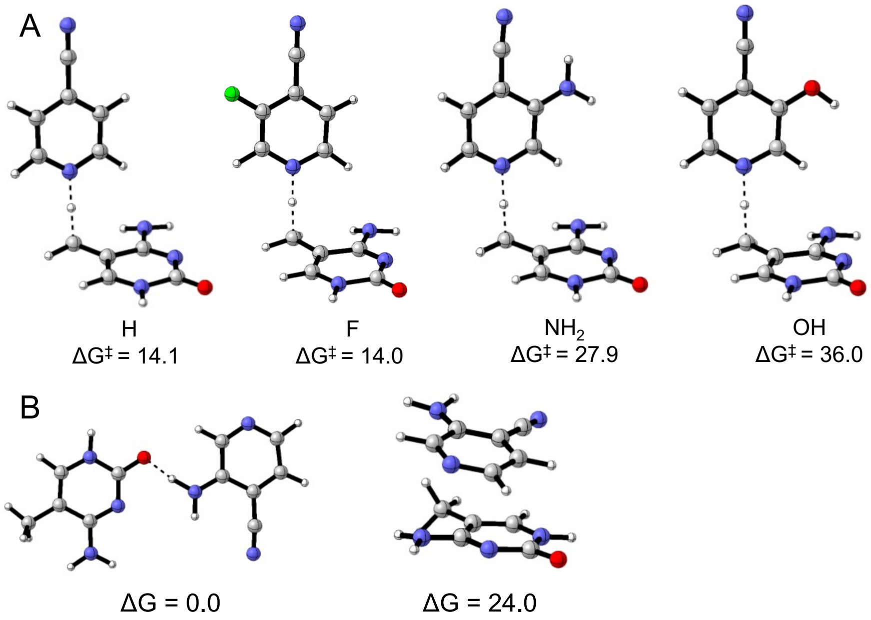

Figure S46: Explanations of the high kinetic barriers for substrates with a H-bond donor group in step 2. A) Mechanism C TS structures with different cyanopyridine substrates. B) Conformers of the cationic 5mdC radical and 3-amino-4cyanopyridine substrate

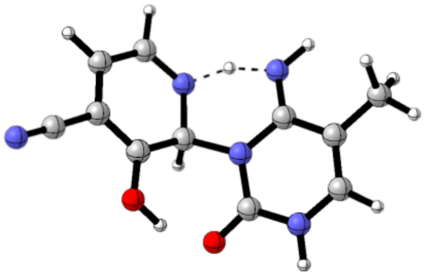

Figure S47: A TS structure unintentionally obtained during the kinetic study for step 2 with 3-hydroxy-4-cyanopyridine

Overall, cyanopyridine substrates with H-bond donor groups are kinetically unfavourable in step 2 of the overall reaction. The kinetic barriers come from the reactant dimer, which either undergoes spontaneous undesirable competitive pathway or is stabilized by additional H bonds.

Section 5.5.: Key structures

The structural information is available in the Cambridge Apollo Repository.

The key structures are included in the 'Key_conformation_SI' folder as opt+freq Gaussian output files. All the structures are the most stable conformer in their corresponding group. The optimizations were conducted at the B3LYP-D3/6-31G(d) level of theory. The filename should be self-explanatory. For example, '4pyCN_xanthone_all_25_radical_2.out' under the step 1/ directory refers to the anionic radical dimer structure of 3,4-dicyanopyridine and xanthone in step 1 of the reaction mechanism (Figure 2 in the main text).

The directory tree of 'Key_conformation_SI' is presented below:

```
Key_conformation_SI_v10122022/
├─ step1/
│   ├─ 4pyCN_xanthone_all_25_radical.out
│   ├─ 4pyF_xanthone_all_9_radical.out
│   ├─ 4pyH_xanthone_all_1_radical.out
│   ├─ 4pyMe_xanthone_all_1_radical.out
│   └─ 4pyONH2_xanthone_all_5_radical.out
├─ step2/
│   ├─ doublet_reactant_dimer_via_neutral_py/
│   │   ├─ 4pyCN_d5mC_12_26.out
│   │   ├─ 4pyF_d5mC_12_101.out
│   │   ├─ 4pyH_d5mc_12_13_2.out
│   │   ├─ 4pyMe_d5mC_12_36.out
│   │   ├─ 4pyNH2_d5mC_12_56.out
│   │   ├─ 4pyOH_d5mc_12_32_2.out
│   │   └─ 4pyONH2_d5mC_12_127.out
│   ├─ doublet_TS_via_neutral_py/
│   │   ├─ A/
│   │   │   ├─ .DS_Store
│   │   │   ├─ 4pyCN_d5mC_12_PA_TS.out
│   │   │   ├─ 4pyF_d5mC_12_PA_TS.out
│   │   │   ├─ 4pyH_d5mc_PA_12_TS.out
│   │   │   ├─ 4pyMe_d5mC_12_PA_TS.out
│   │   │   ├─ 4pyNH2_d5mc_12_PA_TS.out
│   │   │   ├─ 4pyOH_d5mc_12_SA_TS.out
│   │   │   └─ 4pyONH2_d5mC_12_PA_TS.out
│   │   └─ C/
│   │       ├─ 4pyF_d5mC_12_SC_TS.out
│   │       ├─ 4pyH_d5mc_SC_12_TS.out
│   │       ├─ 4pyMe_d5mC_12_SC_TS.out
│   │       ├─ 4pyNH2_d5mc_12_SC2_TS.out
│   │       └─ 4pyOH_d5mc_12_SC_TS.out
│   ├─ Mechanism_A_H_transfer_via_water/
│   │   ├─ C_INT.out
│   │   ├─ C_P.out
│   │   ├─ C_R.out
│   │   ├─ C_TS1.out
│   │   └─ C_TS2.out
│   ├─ triplet_TS_via_anionic_py/
│   │   ├─ PA/
│   │   │   ├─ 4pyCN_d5mC_all_46_TS_2.out
│   │   │   ├─ 4pyF_d5mC_all_26_TS.out
```

```
│    │    │    ├─ 4pyH_d5mc_all_17_TS.out
│    │    │    ├─ 4pyMe_d5mC_all_31_TS.out
│    │    │    ├─ 4pyNH2_d5mc_PA_TS.out
│    │    │    ├─ 4pyOH_d5mc_PA_TS.out
│    │    │    └─ 4pyONH2_d5mC_all_46_TS.out
│    │    ├─ SA/
│    │    │    ├─ 4pyF_d5mC_SA_SA_TS_3.out
│    │    │    ├─ 4pyH_d5mC_SA_SA_TS.out
│    │    │    ├─ 4pyMe_d5mC_all_41_SA_TS.out
│    │    │    ├─ 4pyOH_d5mc_SA_TS.out
│    │    │    └─ 4pyONH2_d5mC_all_73_SA_TS.out
│    │    └─ SC/
│    │         ├─ 4pyCN_d5mC_SC_TS.out
│    │         ├─ 4pyF_d5mC_SC_TS.out
│    │         ├─ 4pyH_d5mc_all_49_SC_TS.out
│    │         ├─ 4pyMe_d5mC_all_96_SC_TS.out
│    │         ├─ 4pyNH2_d5mc_SC_TS.out
│    │         └─ 4pyOH_d5mc_SC_TS_3.out
│    └─ triplet_TS_via_xanthone/
│         ├─ xanthone_d5mC_17_2_SA_TS2.out
│         └─ xanthone_d5mC_17_2_SC_TS.out
├─ step3/
│    ├─ doublet_adduct/
│    │    ├─ 4pyCN_adduct_all_5_doublet.out
│    │    ├─ 4pyF_adduct_all_2_doublet.out
│    │    ├─ 4pyH_adduct_all_6_doublet.out
│    │    ├─ 4pyMe_adduct_all_3_doublet.out
│    │    └─ 4pyONH2_adduct_all_6_doublet.out
│    ├─ doublet_TS/
│    │    ├─ 4pyCN_adduct_all_5_doublet_TS.out
│    │    ├─ 4pyF_adduct_all_2_doublet_TS.out
│    │    ├─ 4pyH_adduct_all_6_doublet_TS.out
│    │    ├─ 4pyMe_adduct_all_3_doublet_TS.out
│    │    └─ 4pyONH2_adduct_all_6_doublet_TS.out
│    ├─ singlet_TS_claisen/
│    │    ├─ 4pyCN_adduct_TS_singlet.out
│    │    ├─ 4pyF_adduct_singlet_unde_TS_2.out
│    │    ├─ 4pyH_adduct_TS_singlet.out
│    │    ├─ 4pyMe_adduct_singlet_unde_TS_2.out
│    │    └─ 4pyONH2_adduct_singlet_unde_TS_3.out
│    └─ triplet_TS/
│         ├─ 4pyCN_adduct_TS.out
│         ├─ 4pyF_adduct_TS.out
│         ├─ 4pyH_adduct_TS.out
│         ├─ 4pyMe_adduct_TS.out
│         └─ 4pyONH2_adduct_all_2_TS.out
└─ step4/
     ├─ d5mC_H_addition/
     │    ├─ H-addition_d5mC(PA)/
     │    │    ├─ .DS_Store
     │    │    ├─ 4pyCN_d5mC_alter_11_PA_TS.out
     │    │    ├─ 4pyF_d5mC_alter_11_PA_TS.out
     │    │    ├─ 4pyH_d5mC_alter_11_PA_TS.out
     │    │    ├─ 4pyMe_d5mC_alter_11_PA_TS.out
     │    │    └─ 4pyONH2_d5mC_alter_11_PA_TS.out
     │    ├─ H-addition_d5mC(PC)/
     │    │    ├─ 4pyCN_d5mC_alter_11_PC_TS.out
     │    │    ├─ 4pyF_d5mC_alter_11_PC_TS.out
```

```
│    │    ├─ 4pyH_d5mC_alter_11_PC_TS.out
│    │    ├─ 4pyMe_d5mC_alter_11_PC_TS.out
│    │    └─ 4pyONH2_d5mC_alter_11_PC_TS.out
│    ├─ H-addition_d5mC(SA)/
│    │    ├─ 4pyCN_d5mC_alter_8_SA_TS.out
│    │    ├─ 4pyF_d5mC_alter_8_SA_TS.out
│    │    ├─ 4pyH_d5mC_alter_8_SA_TS.out
│    │    ├─ 4pyMe_d5mC_alter_8_SA_TS.out
│    │    └─ 4pyONH2_d5mC_alter_32_SA_TS.out
│    └─ H-addition_d5mC(SC)/
│         ├─ 4pyCN_d5mC_alter_8_SC_TS.out
│         ├─ 4pyF_d5mC_alter_1_SC_TS.out
│         ├─ 4pyH_d5mC_alter_1_SC_TS.out
│         ├─ 4pyMe_d5mC_alter_1_SC_TS.out
│         └─ 4pyONH2_d5mC_alter_32_SC_TS.out
└─ water_H_addition/
     ├─ 4pyCN_adduct_water_TS.out
     ├─ 4pyF_adduct_water_TS.out
     ├─ 4pyH_adduct_water_4.out
     ├─ 4pyH_adduct_water_4_TS_2.out
     ├─ 4pyMe_adduct_water_TS.out
     └─ 4pyONH2_adduct_water_TS.out
```

References

[1] D. M. Close, *J. Phys. Chem. B* **2003**, *107*, 864–867.
[2] C. J. Burrows, J. G. Muller, *Chem. Rev.* **1998**, *98*, 1109–1151.
[3] M. Dizdaroglu, P. Jaruga, *Free Radic. Res.* **2012**, *46*, 382–419.
[4] K. J. Koßmann, C. Ziegler, A. Angelin, R. Meyer, M. Skoupi, K. S. Rabe, C. M. Niemeyer, *ChemBioChem* **2016**, 1102–1106.
[5] M. J. Frisch, G. W. Trucks, H. B. Schlegel, G. E. Scuseria, M. A. Robb, J. R. Cheeseman, G. Scalmani, V. Barone, G. A. Petersson, H. Nakatsuji, X. Li, M. Caricato, A. V. Marenich, J. Bloino, B. G. Janesko, R. Gomperts, B. Mennucci, H. P. Hratchian, J. V. Ortiz, A. F. Izmaylov, J. L. Sonnenberg, D. Williams-Young, F. Ding, F. Lipparini, F. Egidi, J. Goings, Peng, A. Petrone, T. Henderson, D. Ranasinghe, V. G. Zakrzewski, J. Gao, N. Rega, G. Zheng, W. Liang, M. Hada, M. Ehara, K. Toyota, R. Fukuda, J. Hasegawa, M. Ishida, T. Nakajima, Y. Honda, O. Kitao, H. Nakai, T. Vreven, K. Throssell, J. A. Montgomery, Jr., J. E. Peralta, F. Ogliaro, M. J. Bearpark, J. J. Heyd, E. N. Brothers, K. N. Kudin, V. N. Staroverov, T. A. Keith, R. Kobayashi, J. Normand, K. Raghavachari, A. P. Rendell, J. C. Burant, S. S. Iyengar, J. Tomasi, M. Cossi, J. M. Millam, M. Klene, C. Adamo, R. Cammi, J. W. Ochterski, R. L. Martin, K. Morokuma, O. Farkas, J. B. Foresman, and D. J. Fox, *Gaussian 16 (Revision B.01)*, Gaussian, Inc., Wallingford CT, 2016.
[6] A. D. Becke, *Phys. Rev. A* **1988**, *38*, 3098–3100.
[7] C. Lee, W. Yang, R. G. Parr, *Phys. Rev. B* **1988**, *37*, 785–789.
[8] A. D. Becke, *J. Chem. Phys.* **1993**, *98*, 5648–5652.
[9] S. Grimme, S. Ehrlich, L. Goerigk, *J. Comput. Chem.* **2011**, *32*, 1456–1465.
[10] J.-D. Chai, M. Head-Gordon, *Phys. Chem. Chem. Phys.* **2008**, *10*, 6615–6620.
[11] *CYLview20.* Université de Sherbrooke. 2020 http://www.cylview.org (accessed November 02, 2020)
[12] *Collaborative Data Science.* Plotly Technologies Inc. 2015. https://plot.ly (accessed February 18, 2021)
[13] G. Luchini, J. V Alegre-Requena, I. Funes-Ardoiz, R. S. Paton, *F1000Research* **2020**, *9*, 291
[14] *Schrödinger Release 2019-01: Maestro*, Schrödinger, LLC, New York, NY, 2019.
[15] T. A. Halgren, *J. Comput. Chem.* **1996**, *17*, 490–519.
[16] *RDKit: Open-Source Cheminformatics.* https://www.rdkit.org/ (accessed December 18, 2020)
[17] T. Hoshikawa, M. Inoue, *Chem. Sci.* **2013**, *4*, 3118–3123.
[18] A. McNally, C. K. Prier, D. W. C. MacMillan, M. Andrew, *Science* **2011**, *334*, 1114–1117.
[19] K. Tanabe, H. Yamada, S.-I. I. Nishimoto, *J. Am. Chem. Soc.* **2007**, *129*, 8034–8040.
[20] J. D. Cuthbertson, D. W. C. MacMillan, *Nature* **2015**, *519*, 74–77.
[21] B. Lipp, A. M. Nauth, T. Opatz, *J. Org. Chem.* **2016**, *81*, 6875–6882.
[22] S. Zhu, J. Qin, F. Wang, H. Li, L. Chu, *Nat. Commun.* **2019**, *10*, 1–7.
[23] C. Hansch, A. Leo, R. W. Taft, *Chem. Rev.* **1991**, *91*, 165–195.
